# Maternal body mass index, gestational weight gain, and the risk of overweight and obesity across childhood: An individual participant data meta-analysis

**DOI:** 10.1371/journal.pmed.1002744

**Published:** 2019-02-11

**Authors:** Ellis Voerman, Susana Santos, Bernadeta Patro Golab, Pilar Amiano, Ferran Ballester, Henrique Barros, Anna Bergström, Marie-Aline Charles, Leda Chatzi, Cécile Chevrier, George P. Chrousos, Eva Corpeleijn, Nathalie Costet, Sarah Crozier, Graham Devereux, Merete Eggesbø, Sandra Ekström, Maria Pia Fantini, Sara Farchi, Francesco Forastiere, Vagelis Georgiu, Keith M. Godfrey, Davide Gori, Veit Grote, Wojciech Hanke, Irva Hertz-Picciotto, Barbara Heude, Daniel Hryhorczuk, Rae-Chi Huang, Hazel Inskip, Nina Iszatt, Anne M. Karvonen, Louise C. Kenny, Berthold Koletzko, Leanne K. Küpers, Hanna Lagström, Irina Lehmann, Per Magnus, Renata Majewska, Johanna Mäkelä, Yannis Manios, Fionnuala M. McAuliffe, Sheila W. McDonald, John Mehegan, Monique Mommers, Camilla S. Morgen, Trevor A. Mori, George Moschonis, Deirdre Murray, Carol Ní Chaoimh, Ellen A. Nohr, Anne-Marie Nybo Andersen, Emily Oken, Adriëtte J. J. M. Oostvogels, Agnieszka Pac, Eleni Papadopoulou, Juha Pekkanen, Costanza Pizzi, Kinga Polanska, Daniela Porta, Lorenzo Richiardi, Sheryl L. Rifas-Shiman, Luca Ronfani, Ana C. Santos, Marie Standl, Camilla Stoltenberg, Elisabeth Thiering, Carel Thijs, Maties Torrent, Suzanne C. Tough, Tomas Trnovec, Steve Turner, Lenie van Rossem, Andrea von Berg, Martine Vrijheid, Tanja G. M. Vrijkotte, Jane West, Alet Wijga, John Wright, Oleksandr Zvinchuk, Thorkild I. A. Sørensen, Debbie A. Lawlor, Romy Gaillard, Vincent W. V. Jaddoe

**Affiliations:** 1 The Generation R Study Group, Erasmus University Medical Center, Rotterdam, the Netherlands; 2 Department of Pediatrics, Erasmus University Medical Center, Rotterdam, the Netherlands; 3 Department of Pediatrics, Medical University of Warsaw, Warsaw, Poland; 4 Public Health Division of Gipuzkoa, San Sebastián, Spain; 5 BioDonostia Research Institute, San Sebastián, Spain; 6 CIBER Epidemiología y Salud Pública (CIBERESP), Madrid, Spain; 7 Epidemiology and Environmental Health Joint Research Unit, FISABIO–Universitat Jaume I–Universitat de València, Valencia, Spain; 8 EPIUnit, Instituto de Saúde Pública, Universidade do Porto, Porto, Portugal; 9 Department of Public Health and Forensic Sciences and Medical Education, Unit of Clinical Epidemiology, Predictive Medicine and Public Health, University of Porto Medical School, Porto, Portugal; 10 Institute of Environmental Medicine, Karolinska Institutet, Stockholm, Sweden; 11 Centre for Occupational and Environmental Medicine, Stockholm County Council, Stockholm, Sweden; 12 INSERM, UMR1153 Epidemiology and Biostatistics Sorbonne Paris Cité Center (CRESS), ORCHAD Team, Villejuif, France; 13 Paris Descartes University, Villejuif, France; 14 Department of Preventive Medicine, Keck School of Medicine, University of Southern California, Los Angeles, California, United States of America; 15 Department of Social Medicine, Faculty of Medicine, University of Crete, Heraklion, Greece; 16 Department of Genetics and Cell Biology, Maastricht University, Maastricht, the Netherlands; 17 INSERM, UMR1085, Irset–Research Institute for Environmental and Occupational Health, Rennes, France; 18 First Department of Pediatrics, National and Kapodistrian University of Athens Medical School, “Aghia Sophia” Children’s Hospital, Athens, Greece; 19 Department of Epidemiology, University of Groningen, University Medical Center Groningen, Groningen, the Netherlands; 20 MRC Lifecourse Epidemiology Unit, University of Southampton, Southampton, United Kingdom; 21 Liverpool School of Tropical Medicine, Liverpool, United Kingdom; 22 Department of Exposure and Environmental Epidemiology, Norwegian Institute of Public Health, Oslo, Norway; 23 The Department of Biomedical and Neuromotor Sciences, University of Bologna, Bologna, Italy; 24 Department of Epidemiology, Lazio Regional Health Service, Rome, Italy; 25 NIHR Southampton Biomedical Research Centre, University of Southampton and University Hospital Southampton NHS Foundation Trust, Southampton, United Kingdom; 26 Division of Metabolic and Nutritional Medicine, Dr. von Hauner Children’s Hospital, Ludwig-Maximilians-Universität München, Munich, Germany; 27 Department of Environmental Epidemiology, Nofer Institute of Occupational Medicine, Lodz, Poland; 28 Department of Public Health Sciences, School of Medicine, University of California, Davis, Davis, California, United States of America; 29 Center for Global Health, University of Illinois College of Medicine, Chicago, Illinois, United States of America; 30 Telethon Kids Institute, The University of Western Australia, Perth, Western Australia, Australia; 31 Department of Health Security, National Institute for Health and Welfare, Kuopio, Finland; 32 Irish Centre for Fetal and Neonatal Translational Research, Cork University Maternity Hospital, University College Cork, Cork, Ireland; 33 Department of Obstetrics and Gynaecology, Cork University Maternity Hospital, Cork, Ireland; 34 Division of Human Nutrition and Health, Wageningen University & Research, Wageningen, the Netherlands; 35 MRC Integrative Epidemiology Unit at the University of Bristol, Bristol, United Kingdom; 36 Population Health Science, Bristol Medical School, University of Bristol, Bristol, United Kingdom; 37 Department of Public Health, University of Turku, Turku, Finland; 38 Department of Environmental Immunology/Core Facility Studies, Helmholtz Centre for Environmental Research–UFZ, Leipzig, Germany; 39 Division of Health Data and Digitalization, Norwegian Institute of Public Health, Oslo, Norway; 40 Department of Epidemiology, Chair of Epidemiology and Preventive Medicine, Jagiellonian University Medical College, Krakow, Poland; 41 Turku Centre for Biotechnology, University of Turku and Abo Akademi University, Turku, Finland; 42 Department of Nutrition and Dietetics, School of Health Science and Education, Harokopio University, Athens, Greece; 43 UCD Perinatal Research Centre, Obstetrics & Gynaecology, School of Medicine, University College Dublin, National Maternity Hospital, Dublin, Ireland; 44 Department of Pediatrics, Cumming School of Medicine, University of Calgary, Calgary, Alberta, Canada; 45 UCD Perinatal Research Centre, School of Public Health and Physiotherapy and Sports Science, University College Dublin, Dublin, Ireland; 46 Department of Epidemiology, Care and Public Health Research Institute, Maastricht University, Maastricht, the Netherlands; 47 National Institute of Public Health, University of Southern Denmark, Copenhagen, Denmark; 48 Department of Public Health, Section of Epidemiology, University of Copenhagen, Copenhagen, Denmark; 49 Medical School, The University of Western Australia, Perth, Western Australia, Australia; 50 Department of Rehabilitation, Nutrition and Sport, La Trobe University, Melbourne, Victoria, Australia; 51 Paediatrics & Child Health, University College Cork, Cork, Ireland; 52 Cork Centre for Vitamin D and Nutrition Research, School of Food and Nutritional Sciences, University College Cork, Cork, Ireland; 53 Research Unit for Gynaecology and Obstetrics, Institute for Clinical Research, University of Southern Denmark, Odense, Denmark; 54 Department of Population Medicine, Harvard Medical School, Harvard Pilgrim Health Care Institute, Boston, Massachusetts, United States of America; 55 Department of Public Health, Amsterdam Public Health Research Institute, Academic Medical Center, Amsterdam, the Netherlands; 56 Department of Environmental Exposures and Epidemiology, Domain of Infection Control and Environmental Health, Norwegian Institute of Public Health, Oslo, Norway; 57 Department of Public Health, University of Helsinki, Helsinki, Finland; 58 Department of Medical Sciences, University of Turin, Turin, Italy; 59 Institute for Maternal and Child Health–IRCCS “Burlo Garofolo”, Trieste, Italy; 60 Institute of Epidemiology, Helmholtz Zentrum München–German Research Center for Environmental Health, Neuherberg, Germany; 61 Norwegian Institute of Public Health, Oslo, Norway; 62 Department of Global Public Health and Primary Care, University of Bergen, Bergen, Norway; 63 Dr. von Hauner Children’s Hospital, Ludwig-Maximilians-Universität München, Munich, Germany; 64 IB-Salut, Area de Salut de Menorca, Palma, Spain; 65 Department of Community Health Sciences, Cumming School of Medicine, University of Calgary, Calgary, Alberta, Canada; 66 Department of Environmental Medicine, Slovak Medical University, Bratislava, Slovak Republic; 67 Child Health, Royal Aberdeen Children’s Hospital, Aberdeen, United Kingdom; 68 Julius Center for Health Sciences and Primary Care, University Medical Center Utrecht, Utrecht University, Utrecht, the Netherlands; 69 Research Institute, Department of Pediatrics, Marien-Hospital Wesel, Wesel, Germany; 70 ISGlobal, Barcelona, Spain; 71 Universitat Pompeu Fabra (UPF), Barcelona, Spain; 72 Bradford Institute for Health Research, Bradford Royal Infirmary, Bradford, United Kingdom; 73 National Institute for Public Health and the Environment, Bilthoven, the Netherlands; 74 Department of Medical and Social Problems of Family Health, Institute of Pediatrics, Obstetrics and Gynecology, Kyiv, Ukraine; 75 The Novo Nordisk Foundation Center for Basic Metabolic Research, Section of Metabolic Genetics, Faculty of Health and Medical Sciences, University of Copenhagen, Copenhagen, Denmark; 76 Department of Epidemiology, Erasmus University Medical Center, Rotterdam, the Netherlands; Chinese University of Hong Kong, CHINA

## Abstract

**Background:**

Maternal obesity and excessive gestational weight gain may have persistent effects on offspring fat development. However, it remains unclear whether these effects differ by severity of obesity, and whether these effects are restricted to the extremes of maternal body mass index (BMI) and gestational weight gain. We aimed to assess the separate and combined associations of maternal BMI and gestational weight gain with the risk of overweight/obesity throughout childhood, and their population impact.

**Methods and findings:**

We conducted an individual participant data meta-analysis of data from 162,129 mothers and their children from 37 pregnancy and birth cohort studies from Europe, North America, and Australia. We assessed the individual and combined associations of maternal pre-pregnancy BMI and gestational weight gain, both in clinical categories and across their full ranges, with the risks of overweight/obesity in early (2.0–5.0 years), mid (5.0–10.0 years) and late childhood (10.0–18.0 years), using multilevel binary logistic regression models with a random intercept at cohort level adjusted for maternal sociodemographic and lifestyle-related characteristics. We observed that higher maternal pre-pregnancy BMI and gestational weight gain both in clinical categories and across their full ranges were associated with higher risks of childhood overweight/obesity, with the strongest effects in late childhood (odds ratios [ORs] for overweight/obesity in early, mid, and late childhood, respectively: OR 1.66 [95% CI: 1.56, 1.78], OR 1.91 [95% CI: 1.85, 1.98], and OR 2.28 [95% CI: 2.08, 2.50] for maternal overweight; OR 2.43 [95% CI: 2.24, 2.64], OR 3.12 [95% CI: 2.98, 3.27], and OR 4.47 [95% CI: 3.99, 5.23] for maternal obesity; and OR 1.39 [95% CI: 1.30, 1.49], OR 1.55 [95% CI: 1.49, 1.60], and OR 1.72 [95% CI: 1.56, 1.91] for excessive gestational weight gain). The proportions of childhood overweight/obesity prevalence attributable to maternal overweight, maternal obesity, and excessive gestational weight gain ranged from 10.2% to 21.6%. Relative to the effect of maternal BMI, excessive gestational weight gain only slightly increased the risk of childhood overweight/obesity within each clinical BMI category (*p*-values for interactions of maternal BMI with gestational weight gain: *p =* 0.038, *p <* 0.001, and *p =* 0.637 in early, mid, and late childhood, respectively). Limitations of this study include the self-report of maternal BMI and gestational weight gain for some of the cohorts, and the potential of residual confounding. Also, as this study only included participants from Europe, North America, and Australia, results need to be interpreted with caution with respect to other populations.

**Conclusions:**

In this study, higher maternal pre-pregnancy BMI and gestational weight gain were associated with an increased risk of childhood overweight/obesity, with the strongest effects at later ages. The additional effect of gestational weight gain in women who are overweight or obese before pregnancy is small. Given the large population impact, future intervention trials aiming to reduce the prevalence of childhood overweight and obesity should focus on maternal weight status before pregnancy, in addition to weight gain during pregnancy.

## Abstract 2

Vincent Jaddoe and colleagues report that a high BMI before pregnancy, rather than excessive gestational weight gain, is more likely to contribute to childhood obesity.

## Author summary

**Why was this study done?:**

- Maternal pre-pregnancy obesity and excessive gestational weight gain are important risk factors of various pregnancy and birth complications.
- An accumulating body of evidence suggests that maternal obesity and excessive gestational weight gain also have persistent effects on offspring fat development.
- It is not clear whether these effects exist across the full ranges of maternal pre-pregnancy body mass index and gestational weight gain, what their combined effects are, and what the population impact of these effects is.

**What did the researchers do and find?:**

- We conducted an individual participant data meta-analysis using data from 162,129 mothers and their children from 37 pregnancy and birth cohorts from Europe, North America, and Australia to assess the separate and combined associations of maternal pre-pregnancy body mass index and gestational weight gain with the risk of overweight/obesity throughout childhood, and their population impact.
- We observed not only that maternal pre-pregnancy overweight and obesity were associated with an increased risk of childhood overweight/obesity, but that this risk increased gradually over the full range of maternal BMI. Similarly, the risk of childhood overweight/obesity increased across the full range of gestational weight gain.
- We estimated that 21.7% to 41.7% of childhood overweight/obesity prevalence could be attributed to maternal overweight and obesity together, whereas 11.4% to 19.2% could be attributed to excessive gestational weight gain.
- The additional effect of excessive gestational weight gain on the risk of childhood overweight/obesity was small among women who are already overweight or obese before pregnancy.

**What do these findings mean?:**

- Maternal pre-pregnancy BMI and, to a smaller extent, gestational weight gain are important modifiable risk factors of childhood overweight/obesity with considerable population impact.
- Future intervention trials aiming to reduce the prevalence of childhood overweight and obesity should focus on maternal weight status before pregnancy, in addition to weight status during pregnancy.

## Introduction

Maternal pre-pregnancy obesity and excessive gestational weight gain are major public health problems. Maternal obesity is an important risk factor of gestational hypertensive and diabetic disorders, fetal death, pre-term birth, and macrosomia [1,2]. An accumulating body of evidence suggests that maternal obesity also has persistent effects on long-term health in offspring [3]. A meta-analysis of published studies showed a 3-fold increased risk of overweight in children of mothers with pre-pregnancy obesity, as compared to those of mothers with a normal pre-pregnancy weight [4]. It remains unclear whether these risks differ by severity of obesity, and whether these effects are restricted to the extremes of maternal BMI or are present across the full range. In addition to maternal pre-pregnancy obesity, excessive gestational weight gain also seems to be associated with increased risks of childhood overweight and obesity [2]. Previous meta-analyses of published studies showed a 30%–40% increased risk of childhood overweight in children of mothers with excessive gestational weight gain [5–7]. From a prevention perspective, insight into the combined effects of maternal BMI and gestational weight gain on offspring obesity risk and their population impact in different geographical regions is needed.

We conducted an individual participant data (IPD) meta-analysis among 162,129 mothers and their children from 37 pregnancy and birth cohorts from Europe, North America, and Australia, to assess the separate and combined associations of maternal pre-pregnancy body mass index (BMI) and gestational weight gain with the risk of overweight/obesity throughout childhood, and their population impact.

## Methods

### Inclusion criteria and participating cohorts

**Fig 1:**
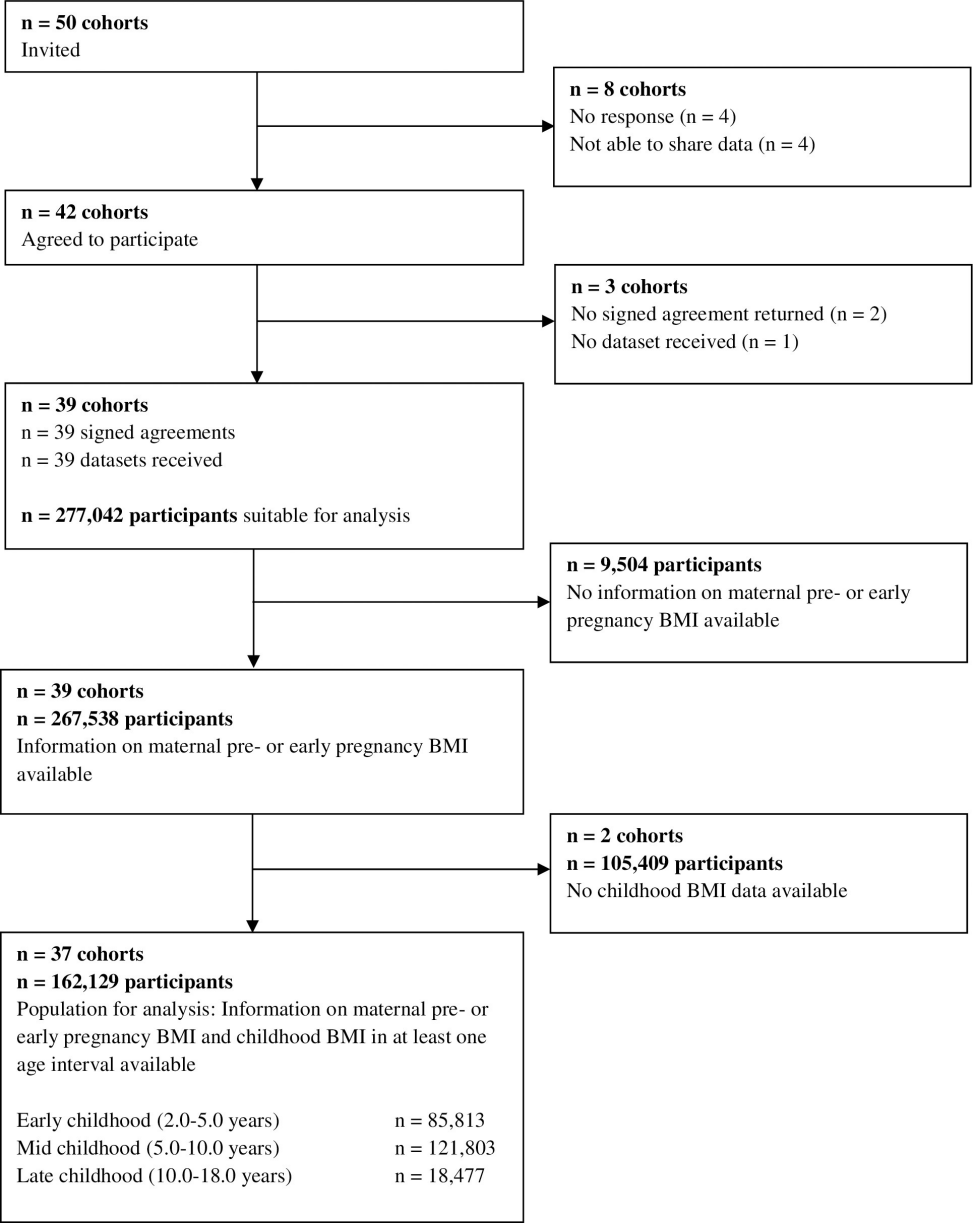
Flow chart of the cohorts and participants.

This study was embedded in the international Maternal Obesity and Childhood Outcomes (MOCO) collaboration [8,9]. Pregnancy and birth cohort studies were eligible to participate if they included mothers with singleton live-born children born from January 1, 1989, onwards, had information available on maternal pre- or early pregnancy BMI and at least 1 offspring measurement (birth weight or childhood BMI), and were approved by their local institutional review boards. We invited 50 Western cohorts from Europe, North America, and Australia, selected from existing collaborations on childhood health (the EarlyNutrition project, the CHICOS project, and Birthcohorts.net assessed until July 2014), of which 39 agreed to participate. In total, 37 cohorts had data available on childhood BMI, corresponding to 162,129 mothers and their children eligible for analyses (Fig 1). All cohorts included were approved by their local institutional review boards, and all participants gave written informed consent. The plan for analyses given to the cohorts when inviting them to participate in the MOCO collaboration is provided in S1 Text. Based on data availability and additional research questions, it was decided among the collaborators to refine the existing questions and to extend the project with additional questions to be addressed. Statistical analyses were adapted to these questions. Anonymized datasets were stored on a single central secured data server with access for the main analysts (EV, SS).

### Maternal pre-pregnancy BMI and gestational weight gain

Maternal BMI (kg/m^2^) was measured, derived from clinical records, or self-reported (cohort-specific information in S1 Table). If available, we used information on maternal pre-pregnancy BMI for analyses. For participants without information on pre-pregnancy BMI (4.8% of the study population), we used BMI measured before 20 weeks of gestation. Maternal pre-pregnancy BMI was categorized into clinical categories according to World Health Organization (WHO) cutoffs (underweight [<18.5 kg/m^2^], normal weight [18.5–24.9 kg/m^2^], overweight [25.0–29.9 kg/m^2^], and obesity [≥30.0 kg/m^2^]). The obesity category was further stratified into obesity class I (30.0–34.9 kg/m^2^), class II (35.0–39.9 kg/m^2^), and class III (≥40.0 kg/m^2^) [10]. Maternal pre-pregnancy BMI was also categorized into 11 groups with a range of 2.5 kg/m^2^ each. Data on gestational weight gain (kg), defined as the difference between the latest weight before delivery and pre-pregnancy weight, was provided by the cohorts, and was categorized as inadequate, adequate, or excessive weight gain in relation to maternal pre-pregnancy BMI according to the guidelines of the US Institute of Medicine (IOM) [11]. We calculated *z*-scores of gestational weight gain based on maternal pre-pregnancy BMI-category-specific reference charts for gestational weight gain by gestational age created using the data of the cohorts participating in this collaboration [8], and categorized them into 6 categories (<−2.0 SD, −2.0 to −1.0 SD, −1.0 to −0.0 SD, 0.0 to 1.0 SD, 1.0 to 2.0 SD, and ≥2.0 SD).

### Childhood overweight/obesity

Childhood BMI (kg/m^2^) was measured, derived from clinical records, or reported by parents/caregivers or the child itself (cohort-specific information in S1 Table). BMI measurements were available in 3 age intervals: early childhood (≥2 to <5 years), mid childhood (≥5 to <10 years), and late childhood (≥10 to <18 years), hereafter referred to as 2.0–5.0, 5.0–10.0, and 10.0–18.0 years, respectively. If there were multiple measurements of a child available within the same age interval, we used the measurement at the highest age for our analyses. We calculated the sex- and age-adjusted standard deviation score (SDS) of childhood BMI based on WHO reference growth charts (Growth Analyser 4.0, Dutch Growth Research Foundation) [12,13]. We categorized childhood BMI into underweight, normal weight, overweight, and obesity, using WHO cutoffs [12,13]. For models focused on the risk of overweight/obesity, children with underweight were excluded, and overweight and obesity were combined. For models focused on the risk of underweight, children with overweight and obesity were excluded.

### Covariates

Information on covariates was mostly assessed by questionnaires. We included as confounders the following: maternal age (<25.0 years, 25.0–30.0 years, 30–35.0 years, ≥35.0 years), maternal educational level (low, medium, high), maternal ethnicity (European/white, non-European/non-white), parity (nulliparous, multiparous), and maternal smoking during pregnancy (yes, no).

### Statistical analysis

We conducted 1-stage meta-analyses, analyzing IPD from all cohorts simultaneously in multilevel linear or binary logistic regression models, accounting for clustering of participants within cohorts [14]. In these models, we included a random intercept at the cohort level, allowing intercepts to differ between cohorts. First, we examined the separate associations of maternal pre-pregnancy BMI and gestational weight gain, across their full ranges and in clinical categories, with BMI SDS, the risk of childhood underweight, and the risk of childhood overweight/obesity in early, mid, and late childhood. Second, we calculated, both for the total study population and per country, the population attributable risk fraction (PAR), indicating the proportion of childhood underweight and childhood overweight/obesity attributable to each maternal BMI or gestational weight gain category. For these analyses, we used the adjusted odds ratio (OR) and the prevalence of the exposure in the study population [15]. Country-specific analyses were performed for mid childhood only, based on available data. Third, we assessed the associations of the combinations of maternal pre-pregnancy BMI and gestational weight gain clinical categories with the outcomes. To assess whether the combined effects of maternal BMI and gestational weight gain on the outcomes were different from the separate effects, we tested for interaction between these 2 exposures.

Models were adjusted for maternal age, educational level, ethnicity, parity, and smoking during pregnancy. Models concerning gestational weight gain *z-*scores were additionally adjusted for maternal pre-pregnancy BMI. To examine whether any associations of maternal pre-pregnancy BMI with the risk of childhood overweight/obesity were explained by gestational diabetes, gestational hypertensive disorders, or gestational-age-adjusted birth weight, we added this information to the models. Covariates in the analyses were selected based on existing literature and data availability in participating cohorts. Findings from the unadjusted models were similar to the findings from the adjusted models and therefore are not presented separately. We did not observe consistent significant interactions of maternal pre-pregnancy BMI and gestational weight gain with child’s sex. As sensitivity analyses, we conducted 2-stage random effects meta-analyses to study the associations of maternal pre-pregnancy BMI and gestational weight gain with the risk of childhood overweight/obesity in each cohort and to test for heterogeneity between estimates [14]. All covariates were categorized. To deal with missing values of covariates, we used the missing values as an additional category, to prevent exclusion of non-complete cases. Exposures and outcomes were not imputed. If information on a covariate was available for less than 50% of the cohort sample used for each analysis, available information was not used and the corresponding data for that full cohort sample was assigned to the missing category. We also conducted a sensitivity analysis with complete cases only.

The statistical analyses were performed using the IBM SPSS Statistics version 21.0 for Windows (IBM, Armonk, NY, US), RevMan version 5.3 (Nordic Cochrane Centre, Copenhagen, Denmark), and R statistical software version 3.3.3.

## Results

### Participants’ characteristics

Table 1 shows that the median maternal pre-pregnancy BMI was 22.7 kg/m^2^ (95% range: 18.1, 34.3) and the median gestational weight gain was 14.0 kg (95% range: 4.0, 26.0). Of all children, 6.5%, 20.1%, and 22.2% were overweight/obese in early, mid, and late childhood, respectively. The country-specific prevalences of maternal overweight and obesity, excessive gestational weight gain, and mid childhood overweight/obesity ranged 12.9%–53.1%, 22.2%–57.0%, and 10.6%–43.1%, respectively (S1 Fig). S2 Table shows cohort-specific information on covariates.

**Table 1:** Cohort-specific description of exposures and outcomes.

Cohort name, number of participants, birth years (country)	Maternal characteristics		Early childhood characteristics (2.0–5.0 years)			Mid childhood characteristics (5.0–10.0 years)			Late childhood characteristics (10.0–18.0 years)		
	Pre-/early pregnancy BMI (kg/m^2^)	Gestational weight gain (kg)	Age (months)	BMI (SDS)	Overweight/obesity, *n* (%)	Age (months)	BMI (SDS)	Overweight/obesity, *n* (%)	Age (months)	BMI (SDS)	Overweight/obesity, *n* (%)
ABCD, *n =* 5,494, 2003–2004 (the Netherlands)	22.2 (17.9, 33.9)	NA	47.2 (25.5, 54.3)	0.28 (−1.52, 2.37)	212 (4.4)	68.2 (61.6, 82.2)	0.10 (−1.69, 2.38)	766 (17.1)	NA	NA	NA
ALSPAC, *n =* 8,435, 1991–1992 (UK)	22.3 (18.0, 33.6)	12.5 (4.0, 22.0)	48.7 (30.8, 49.7)	0.61 (−1.00, 2.45)	71 (6.4)	115.0 (88.0, 119.0)	0.24 (−1.60, 2.68)	2,001 (26.5)	165.0 (126.0, 171.0)	0.23 (−1.85, 2.53)	1,908 (26.0)
AOB/F, *n =* 1,653, 2008–2010 (Canada)	23.0 (18.0, 38.2)	NA	36.0 (35.0, 42.0)	0.23 (−2.27, 2.65)	94 (5.7)	NA	NA	NA	NA	NA	NA
BAMSE, *n =* 2,930, 1994–1996 (Sweden)	22.3 (18.2, 31.6)^a^	13.0 (5.6, 25.0)	51.3 (48.2, 57.6)	0.57 (−0.94, 2.47)	152 (6.0)	100.0 (89.0, 109.0)	0.51 (−1.19, 2.63)	695 (31.0)	201.2 (191.7, 210.2)	0.09 (−1.73, 2.03)	373 (16.9)
BIB, *n =* 887, 2007–2010 (UK)	24.8 (17.6, 39.4)^a^	10.0 (0.0, 20.5)	36.8 (35.8, 39.3)	0.49 (−1.40, 2.54)	64 (7.2)	NA	NA	NA	NA	NA	NA
CHOP, *n =* 905, 2002–2004 (multiple)	22.4 (17.6, 33.7)	NA	48.6 (24.0, 60.0)	0.26 (−1.58, 2.51)	24 (5.8)	96.1 (65.9, 99.2)	0.32 (−1.72, 2.92)	201 (27.8)	NA	NA	NA
Co.N.ER, *n =* 522, 2004–2005 (Italy)	21.2 (17.7, 30.4)	13.0 (6.0, 22.1)	43.9 (34.8, 54.8)	0.27 (−2.28, 2.92)	47 (9.8)	94.9 (86.6, 111.2)	0.69 (−1.29, 2.82)	100 (35.3)	NA	NA	NA
DNBC, *n =* 39,637, 1996–2002 (Denmark)	22.5 (18.1, 33.6)	15.0 (5.0, 28.0)	NA	NA	NA	85.0 (75,6, 89.5)	0.01 (−1.95, 2.08)	6,138 (15.5)	NA	NA	NA
EDEN, *n =* 1,331, 2003–2005 (France)	22.1 (17.4, 35.0)	13.0 (4.0, 23.0)	38.0 (36.9, 40.0)	0.29 (−1.46, 1.96)	26 (2.2)	67.6 (65.0, 72.4)	−0.02 (−1.52, 1.92)	140 (12.5)	NA	NA	NA
FCOU, *n =* 2,107, 1993–1996 (Ukraine)	21.8 (17.3, 32.1)	12.0 (3.5, 21.0)	35.0 (24.0, 40.0)	0.55 (−1.93, 3.14)	134 (11.1)	84.0 (75.0, 93.4)	−0.02 (−2.01, 2.06)	111 (12.6)	194.0 (183.0, 209.0)	−0.09 (−2.06, 1.82)	70 (9.1)
GASPII, *n =* 568, 2003–2004 (Italy)	21.3 (17.6, 31.1)	13.0 (6.0, 24.0)	50.0 (43.0, 53.0)	0.71 (−1.06, 2.94)	52 (9.7)	104.0 (98.0, 113.0)	0.70 (−1.37, 2.66)	171 (37.1)	NA	NA	NA
GECKO Drenthe, *n =* 1,963, 2006–2007 (the Netherlands)	23.7 (18.6, 36.8)	13.0 (4.0, 25.0)	NA	NA	NA	70.4 (62.6, 78.6)	0.39 (−1.16, 2.43)	426 (21.7)	NA	NA	NA
GENERATION R, *n =* 6,716, 2002–2006 (the Netherlands)	22.8 (18.1, 34.9)	13.0 (1.0, 25.0)	45.8 (44.4, 48.6)	0.30 (−1.44, 2.48)	186 (4.9)	115.3 (69.4, 119.4)	0.36 (−1.52, 2.70)	1,661 (27.5)	122.1 (120.1, 137.8)	0.36 (−1.51, 2.62)	144 (29.8)
GENERATION XXI, *n =* 5,940, 2005–2006 (Portugal)	23.0 (18.2, 34.7)	13.0 (3.0, 26.0)	50.0 (46.0, 58.0)	0.52 (−1.27, 3.07)	483 (10.3)	85.0 (83.0, 95.0)	0.63 (−1.38, 3.23)	2,015 (37.9)	NA	NA	NA
GENESIS, *n =* 1,898, 2003–2004 (Greece)	21.8 (17.6, 30.9)	13.0 (3.0, 28.6)	43.6 (26.1, 57.8)	0.83 (−1.17, 3.57)	257 (14.2)	62.0 (60.1. 71.9)	0.93 (−1.44, 4.13)	38 (43.2)	NA	NA	NA
GINIplus, *n =* 2,326, 1995–1998 (Germany)	22.1 (18.0, 31.4)	13.0 (5.0, 25.0)	48.0 (44.0, 52.0)	0.08 (−1.72, 2.00)	54 (2.5)	62.9 (60.2, 75.0)	0.01 (−1.77, 1.94)	231 (10.7)	182.0 (177.0, 191.0)	0.01 (−1.88, 2.08)	366 (15.9)
HUMIS, *n =* 945, 2003–2008 (Norway)	23.3 (18.4, 35.0)	14.0 (5.0, 27.0)	25.7 (24.0, 37.4)	0.33 (−1.83, 2.39)	52 (6.1)	84.0 (60.0, 92.0)	0.04 (−2.02, 2.35)	62 (17.6)	NA	NA	NA
INMA, *n =* 1,916, 1997–2008 (Spain)	22.5 (18.0, 34.6)	13.5 (4.2, 24.4)	52.9 (49.0, 56.5)	0.50 (−1.21, 2.82)	142 (8.2)	83.8 (74.8, 94.5)	0.58 (−1.34, 3.32)	498 (37.7)	174.5 (172.0, 181.5)	0.32 (−1.59, 2.47)	76 (25.3)
KOALA, *n =* 2,051, 2000–2002 (the Netherlands)	22.8 (18.5, 33.5)	14.0 (4.0, 25.0)	55.5 (48.1, 59.7)	−0.07 (−2.00, 1.83)	16 (1.6)	106.0 (61.5, 119.3)	−0.17 (−2.16, 1.77)	198 (11.3)	121.4 (120.0, 126.7)	−0.16 (−2.06, 2.22)	19 (18.1)
Krakow Cohort, *n =* 422, 2000–2003 (Poland)	21.1 (17.3, 28.0)	15.0 (7.0, 28.0)	48.0 (36.0, 51.3)	−0.06 (−2.24, 2.28)	11 (4.1)	108.0 (60.0, 111.0)	0.18 (−1.86, 2.56)	90 (26.5)	NA	NA	NA
LISAplus, *n =* 2,334, 1997–1999 (Germany)	21.7 (17.9, 32.9)	14.0 (6.0, 24.5)	48.0 (44.0, 52.0)	0.07 (−1.83, 1.98)	53 (2.5)	62.7 (60.2, 74.0)	−0.09 (−1.92, 1.91)	207 (10.4)	181.0 (121.0, 191.0)	−0.02 (−1.88, 2.08)	293 (15.9)
LUKAS, *n =* 379, 2002–2005 (Finland)	24.0 (18.5, 36.6)	13.9 (3.9, 25.1)	48.2 (46.2, 50.0)	0.52 (−1.28, 2.81)	30 (7.9)	73.2 (68.6, 76.0)	0.52 (−1.08, 3.34)	112 (31.0)	NA	NA	NA
MoBa, *n =* 54,910, 1999–2009 (Norway)	23.1 (18.4, 34.7)	14.5 (4.0, 27.0)	36.4 (25.5, 60.0)	0.37 (−1.84, 2.46)	2,447 (6.1)	86.9 (61.0, 100.9)	0.14 (−2.06, 2.30)	6,774 (19.4)	NA	NA	NA
NINFEA, *n =* 1,753, 2005–2010 (Italy)^b^	21.4 (17.4, 31.9)	12.0 (3.0, 22.0)	49.7 (48.2, 57.1)	0.09 (−2.33, 2.52)	88 (5.1)	86.1 (84.8, 93.0)	−0.03 (−2.17, 2.43)	91 (21.0)	NA	NA	NA
PÉLAGIE, *n =* 738, 2002–2005 (France)	21.7 (17.5, 32.4)	NA	24.4 (24.0, 26.5)	0.12 (−1.84, 1.95)	16 (2.2)	NA	NA	NA	NA	NA	NA
PIAMA, *n =* 2,324, 1996–1997 (the Netherlands)	22.2 (18.4, 31.5)	13.0 (5.0, 25.0)	49.3 (44.2, 54.5)	0.69 (−1.20, 2.58)	105 (9.3)	97.5 (90.9, 110.6)	0.15 (−1.68, 2.35)	417 (20.5)	195.9 (192.5, 203.4)	−0.16 (−1.74, 1.80)	72 (9.5)
Piccolipiù, *n =* 687, 2011–2015 (Italy)	21.6 (17.6, 31.8)	13.0 (6.0, 21.2)	24.0 (24.0, 28.0)	0.36 (−2.16, 2.55)	40 (5.8)	NA	NA	NA	NA	NA	NA
Project Viva, *n =* 1,382, 1999–2002 (US)	23.5 (18.3, 38.2)	15.5 (5.0, 27.3)	37.9 (36.1, 50.2)	0.66 (−1.01, 2.69)	86 (7.0)	92.2 (82.5, 116.6)	0.44 (−1.43, 3.05)	326 (30.7)	123.8 (120.6, 131.1)	0.38 (−1.50, 3.76)	8 (25.8)
Raine Study, *n =* 2,092, 1989–1992 (Australia)	21.3 (17.1, 34.0)	NA	NA	NA	NA	71.0 (66.8, 77.1)	0.15 (−1.57, 2.75)	384 (20.0)	126.9 (125.0, 133.3)	0.45 (−1.62, 2.84)	566 (33.3)
REPRO_PL, *n =* 283, 2007–2011 (Poland)	21.6 (17.2, 32.8)	12.5 (2.3, 23.0)	25.0 (24.0, 31.0)	0.31 (−2.13, 2.51)	19 (7.1)	88.0 (84.3, 94.0)	0.64 (−1.55, 3.64)	19 (38.8)	NA	NA	NA
RHEA, *n =* 748, 2007–2008 (Greece)	23.4 (18.1, 36.4)	13.0 (4.0, 26.0)	49.8 (48.0, 57.5)	0.60 (−1.13, 3.58)	92 (12.3)	NA	NA	NA	NA	NA	NA
ROLO, *n =* 290, 2007–2011 (Ireland)	25.3 (20.1, 38.7)^a^	12.1 (2.1, 22.7)	24.7 (24.0, 34.0)	0.20 (−1.75, 2.62)	19 (6.6)	NA	NA	NA	NA	NA	NA
SCOPE BASELINE, *n =* 1,045, 2008–2011 (Ireland)	24.0 (19.3, 34.8)^a^	14.3 (7.3, 23.3)	25.5 (24.5, 28.9)	0.65 (−1.02, 2.32)	62 (5.9)	NA	NA	NA	NA	NA	NA
SEATON, *n =* 933, 1998–1999 (UK)	24.0 (18.8, 37.9)^a^	NA	58.6 (55.9, 59.9)	0.65 (−0.89, 2.68)	37 (7.8)	61.2 (60.0, 119.7)	0.59 (−1.10, 2.73)	58 (19.8)	180.1 (121.5, 186.0)	0.43 (−1.61, 2.60)	199 (31.6)
Slovak PCB study, *n =* 480, 2002–2004 (Slovak Republic)	21.2 (16.7, 31.6)	14.0 (4.1, 24.8)	45.4 (44.8, 49.9)	1.95 (−2.46, 5.29)	212 (48.7)	93.0 (85.0, 100.0)	0.32 (−1.73, 3.22)	117 (32.1)	NA	NA	NA
STEPS, *n =* 484, 2008–2010 (Finland)	22.8 (18.3, 36.9)	14.1 (1.6, 25.5)	36.8 (35.6, 38.4)	0.56 (−1.09, 2.18)	20 (4.1)	NA	NA	NA	NA	NA	NA
SWS, *n =* 2,621, 1998–2007 (United Kingdom)	24.2 (18.9, 37.4)	11.9 (0.4, 25.2)	38.4 (35.6, 50.7)	0.49 (−1.27, 2.57)	155 (6.1)	80.4 (74.7, 87.2)	0.21 (−1.52, 2.54)	392 (22.0)	NA	NA	NA
**Total group**	22.7 (18.1, 34.3)	14.0 (4.0, 26.0)	38.2 (24.5, 60.0)	0.39 (−1.69, 2.58)	5,558 (6.5)	85.3 (61.0, 117.4)	0.14 (−1.85, 2.44)	24,439 (20.1)	168.0 (121.8, 203.7)	0.14 (−1.81, 2.41)	4,094 (22.2)

Values are expressed as median (95% range) or number of participants (valid percent).

^a^Only information available on BMI assessed in early pregnancy (<20 weeks of gestation).

^b^Subset of participants with 4 years of follow-up completed.

NA, not available; SDS, standard deviation score.

### Maternal pre-pregnancy BMI and gestational weight gain clinical categories

Table 2 shows that, as compared to maternal normal weight, maternal underweight was associated with lower risks of overweight/obesity throughout childhood (*p*-values < 0.05). As compared to maternal normal weight, maternal overweight and obesity were associated with higher risks of overweight/obesity throughout childhood, with stronger associations at later ages (ORs for overweight/obesity in late childhood: 2.28 [95% CI: 2.08, 2.50] and 4.47 [95% CI: 3.99, 5.23] for maternal overweight and obesity, respectively). Among women with obesity, the risk of offspring overweight/obesity increased further for higher classes of maternal obesity (ORs for overweight/obesity in late childhood: 4.16 [95% CI: 3.56, 4·87], 5.98 [95% CI: 4.50, 7.94], and 5.55 [95% CI: 3.25, 9.45] for obesity class I, class II, and class III, respectively, as compared to normal weight; Table 2). These associations were not explained by gestational diabetes or gestational hypertensive disorders (S3 and S4 Tables). Additional adjustment for gestational-age-adjusted birth weight attenuated the associations only slightly (S5 Table). As compared to adequate gestational weight gain, inadequate gestational weight gain was associated with a lower risk of overweight/obesity in early and mid childhood (*p*-values < 0.05), but not in late childhood. As compared to adequate gestational weight gain, excessive gestational weight gain was associated with a higher risk of childhood overweight/obesity in early, mid, and late childhood (ORs 1.39 [95% CI: 1.30, 1.49], 1.55 [95% CI: 1.49, 1.60], and 1.72 [95% CI: 1.56, 1.91], respectively). S6 Table shows that, as compared to maternal normal weight, maternal underweight was associated with a higher risk of childhood underweight, whereas maternal overweight and obesity were associated with a lower risk of childhood underweight in early, mid, and late childhood. Similarly, as compared to adequate gestational weight gain, inadequate gestational weight gain was associated with higher risks of childhood underweight, and excessive gestational weight gain with lower risks. The associations of maternal BMI and gestational weight gain clinical categories with childhood BMI SDS are presented in Table 3.

**Table 2:** Associations of maternal pre-pregnancy BMI and gestational weight gain clinical categories with the risk of childhood overweight/obesity.

Clinical category	Childhood overweight/obesity					
	Early childhood 2.0–5.0 years		Mid childhood 5.0–10.0 years		Late childhood 10.0–18.0 years	
	OR (95% CI)	PAR (%)	OR (95% CI)	PAR (%)	OR (95% CI)	PAR (%)
**Maternal pre-pregnancy BMI**						
Underweight (<18.5 kg/m^2^)	0.57 (0.47, 0.69) *n*_cases/total_ = 126/3,162	NA	0.44 (0.40, 0.49) *n*_cases/total_ = 401/4,485	NA	0.44 (0.35, 0.55) *n*_cases/total_ = 93/877	NA
Normal weight (18.5–24.9 kg/m^2^)	Reference *n*_cases/total_ = 3,092/57,293		Reference *n*_cases/total_ = 13,870/82,438		Reference *n*_cases/total_ = 2,505/13,497	
Overweight (25.0–29.9 kg/m^2^)	1.66 (1.56, 1.78) *n*_cases/total_ = 1,476/17,013	11.5	1.91 (1.85, 1.98) *n*_cases/total_ = 6,556/23,359	15.1	2.28 (2.08, 2.50) *n*_cases/total_ = 968/2,799	20.1
Obesity (≥30.0 kg/m^2^)	2.43 (2.24, 2.64) *n*_cases/total_ = 864/7,058	10.2	3.12 (2.98, 3,27) *n*_cases/total_ = 3,612/9,248	14.4	4.47 (3.99, 5.23) *n*_cases/total_ = 528/1,000	21.6
Obesity class I (30.0–34.9 kg/m^2^)	2.35 (2.14, 2.59) *n*_cases/total_ = 613/5,142	7.3	2.89 (2.74, 3.05) *n*_cases/total_ = 2,552/6,874	10.0	4.16 (3.56, 4.87) *n*_cases/total_ = 363/726	15.6
Obesity class II (35.0–39.9 kg/m^2^)	2.57 (2.20, 3.02) *n*_cases/total_ = 190/1,489	2.5	3.57 (3.24, 3.93) *n*_cases/total_ = 782/1,836	3.9	5.98 (4.50, 7.94) *n*_cases/total_ = 129/215	7.4
Obesity class III (≥40.0 kg/m^2^)	2.93 (2.22, 3.87) *n*_cases/total_ = 61/427	0.9	5.17 (4.35, 6.15) *n*_cases/total_ = 278/538	2.0	5.55 (3.25, 9.45) *n*_cases/total_ = 36/59	2.1
**Gestational weight gain**						
Inadequate weight gain	0.86 (0.78, 0.93) *n*_cases/total_ = 853/15,484	NA	0.90 (0.84, 0.92) *n*_cases/total_ = 3,515/20,596	NA	0.91 (0.82, 1.02) *n*_cases/total_ = 705/3,897	NA
Adequate weight gain	Reference *n*_cases/total_ = 1,542/25,418		Reference *n*_cases/total_ = 6,404/36,391		Reference *n*_cases/total_ = 944/5,152	
Excessive weight gain	1.39 (1.30, 1.49) *n*_cases/total_ = 2,265/27,590	11.4	1.55 (1.49, 1.60) *n*_cases/total_ = 9,884/40,201	15.4	1.72 (1.56, 1.91) *n*_cases/total_ = 1,061/3,703	19.2

Values are odds ratios (ORs) (95% confidence intervals) from multilevel binary logistic regression models that reflect the risk of childhood overweight/obesity in early childhood (2.0–5.0 years), mid childhood (5.0–10.0 years), and late childhood (10.0–18.0 years) in children of mothers in the different pre-pregnancy BMI groups or gestational weight gain groups, as compared with the reference group (normal weight for pre-pregnancy BMI and adequate weight gain for gestational weight gain), or population attributable risk fractions (PARs), indicating the proportion of childhood overweight/obesity cases attributable to each maternal BMI or gestational weight gain category. The models are adjusted for maternal age, education level, ethnicity, parity, and smoking during pregnancy.

NA, not applicable.

**Table 3:** Associations of maternal pre-pregnancy BMI and gestational weight gain clinical categories with childhood BMI standard deviation score (SDS).

Clinical category	Childhood BMI standard deviation score		
	Early childhood 2.0–5.0 years	Mid childhood 5.0–10.0 years	Late childhood 10.0–18.0 years
**Maternal pre-pregnancy BMI**			
Underweight (<18.5 kg/m^2^)	−0.29 (−0.33, −0.26)	−0.41 (−0.45, −0.38)	−0.48 (−0.54, −0.41)
	*n =* 3,240	*n =* 4,673	*n =* 917
Normal weight (18.5–24.9 kg/m^2^)	Reference	Reference	Reference
	*n =* 58,177	*n =* 84,119	*n =* 13,737
Overweight (25.0–29.9 kg/m^2^)	0.19 (0.17, 0.21)	0.33 (0.32, 0.35)	0.45 (0.41, 0.49)
	*n =* 17,258	*n =* 23,671	*n =* 2,819
Obesity (≥30.0 kg/m^2^)	0.34 (0.32, 0.37)	0.62 (0.60, 0.64)	0.92 (0.86, 0.99)
	*n =* 7,138	*n =* 9,340	*n =* 1,004
Obesity class I (30.0–34.9 kg/m^2^)	0.32 (0.29, 0.35)	0.57 (0.54, 0.59)	0.85 (0.76, 0.93)
	*n =* 5,197	*n =* 6,944	*n =* 730
Obesity class II (35.0–39.9 kg/m^2^)	0.39 (0.33, 0.44)	0.72 (0.67, 0.77)	1.09 (0.96, 1.23)
	*n =* 1,509	*n =* 1,854	*n =* 215
Obesity class III (≥40.0 kg/m^2^)	0.45 (0.35, 0.55)	0.94 (0.85, 1.03)	1.18 (0.92, 1.44)
	*n =* 343	*n =* 542	*n =* 59
**Gestational weight gain**			
Inadequate weight gain	−0.10 (−0.12, −0.08)	−0.09 (−0.11, −0.07)	−0.09 (−0.13, −0.05)
	*n =* 15,782	*n =* 21,094	*n =* 3,998
Adequate weight gain	Reference	Reference	Reference
	*n =* 25,829	*n =* 37,142	*n =* 5,247
Excessive weight gain	0.14 (0.12, 0.16)	0.22 (0.21, 0.24)	0.28 (0.24, 0.32)
	*n =* 27,965	*n =* 40,879	*n =* 3,742

Values are regression coefficients (95% confidence intervals) from multilevel linear regression models that reflect differences in BMI SDS in early childhood (2.0–5.0 years), mid childhood (5.0–10.0 years), and late childhood (10.0–18.0 years) in children of mothers in the different pre-pregnancy BMI groups or gestational weight gain groups, as compared with the reference group (normal weight for pre-pregnancy BMI and adequate weight gain for gestational weight gain). The models are adjusted for maternal age, education level, ethnicity, parity, and smoking during pregnancy.

The estimated proportions of childhood overweight/obesity attributable to maternal pre-pregnancy overweight and obesity were respectively 11.5% and 10.2% in early childhood, 15.1% and 14.4% in mid childhood, and 20.1% and 21.6% in late childhood (Table 2). The estimated proportions of childhood overweight/obesity attributable to excessive gestational weight gain were 11.4%, 15.4%, and 19.2%, in early, mid, and late childhood, respectively (Table 2). The country-specific proportions of mid childhood overweight/obesity are given in S2 Fig.

### Maternal pre-pregnancy BMI and gestational weight gain across their full ranges

**Fig 2:**
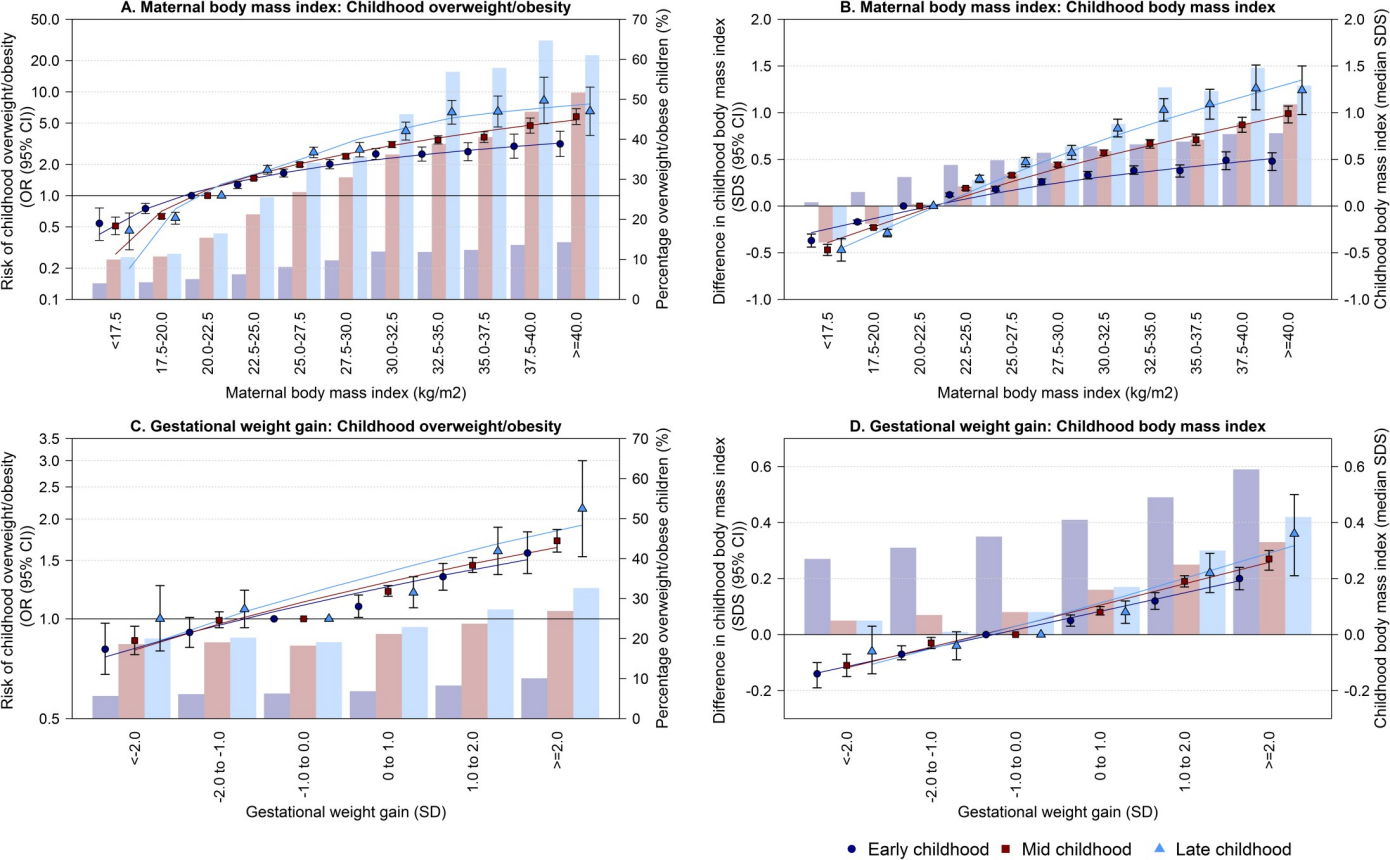
Associations of maternal pre-pregnancy BMI and gestational weight gain with the risk of overweight/obesity and childhood BMI. The circles, squares and triangles represent odds ratios (ORs) (A and C) or regression coefficients (B and D) (95% confidence intervals) obtained from multilevel binary logistic or linear regression models that reflect the risk of overweight/obesity or differences in early, mid, and late childhood BMI standard deviation score (SDS) in the different maternal pre-pregnancy BMI or gestational weight gain groups, as compared to the reference group (20.0–22.5 kg/m^2^ for maternal BMI, -1.0 to 0.0 SD for gestational weight gain [largest groups], primary *y*-axis). The lines are trendlines through the estimates. The models are adjusted for maternal age, education level, ethnicity, parity, and smoking during pregnancy. The bars represent the percentage overweight/obese children (A and C) or the median childhood BMI SDS (B and D) in early (2.0–5.0 years, violet bars), mid (5.0–10.0 years, brown bars), and late childhood (10.0–18.0 years, light blue bars) in the study population (secondary *y*-axis).

Fig 2A and 2B show that higher maternal pre-pregnancy BMI was across the full range associated with higher risk of offspring overweight/obesity and higher offspring BMI SDS throughout childhood. The ORs for childhood overweight/obesity per kg/m^2^ increase in maternal pre-pregnancy BMI were 1.08 (95% CI: 1.07, 1.09), 1.12 (95% CI: 1.11, 1.12), and 1.16 (95% CI: 1.15, 1.17), in early, mid, and late childhood, respectively. Similarly, higher maternal gestational weight gain across its full range was associated with a higher risk of overweight/obesity and higher childhood BMI in early, mid, and late childhood (Fig 2C and 2D). The ORs for childhood overweight/obesity per SD increase in gestational weight gain were 1.14 (95% CI: 1.11, 1.17), 1.16 (95% CI: 1.14, 1.18), and 1.14 (95% CI: 1.09, 1.20), in early, mid, and late childhood, respectively. Similar results were observed when performing 2-stage random effects meta-analyses, with low to moderate heterogeneity (S3 and S4 Figs).

### Combined effects of maternal pre-pregnancy BMI and gestational weight gain clinical categories

Table 4 shows the combined effect of clinical categories of maternal pre-pregnancy BMI and gestational weight gain on childhood overweight/obesity. Regardless of their mothers’ gestational weight gain, children of mothers with underweight tended to have a lower risk of overweight/obesity, whereas children of mothers with overweight or obesity had a higher risk of overweight/obesity, as compared to children whose mothers had normal weight and adequate gestational weight gain. Within each maternal BMI category, excessive gestational weight gain tended to increase the risk of overweight/obesity in early and mid childhood only slightly. The combined associations of clinical categories of maternal pre-pregnancy BMI and gestational weight gain with childhood BMI SDS are given in Table 5.

**Table 4:** Combined associations of maternal pre-pregnancy BMI and gestational weight gain clinical categories with the risk of childhood overweight/obesity.

Maternal pre-pregnancy BMI	Childhood overweight/obesity		
	Early childhood 2.0–5.0 years	Mid childhood 5.0–10.0 years	Late childhood 10.0–18.0 years
**Underweight (<18.5 kg/m**^**2**^**)**			
Inadequate weight gain	0.58 (0.42, 0.81) *n*_cases/total_ = 40/970	0.39 (0.31, 0.48) *n*_cases/total_ = 97/1,284	0.36 (0.22, 0.58) *n*_cases/total_ = 19/266
Adequate weight gain	0.58 (0.42, 0.80) *n*_cases/total_ = 44/1,087	0.46 (0.38, 0.56) *n*_cases/total_ = 126/1,538	0.37 (0.22, 0.62) *n*_cases/total_ = 16/226
Excessive weight gain	0.75 (0.49, 1.15) *n*_cases/total_ = 25/455	0.75 (0.60, 0.95) *n*_cases/total_ = 85/635	1.28 (0.67, 2.42) *n*_cases/total_ = 13/56
**Normal weight (18.5–24.9 kg/m**^**2**^**)**			
Inadequate weight gain	0.87 (0.78, 0.96) *n*_cases/total_ = 583/12,027	0.91 (0.86, 0.96) *n*_cases/total_ = 2,462/16,163	0.95 (0.84, 1.08) *n*_cases/total_ = 536/3,255
Adequate weight gain	Reference *n*_cases/total_ = 1,032/19,502	Reference *n*_cases/total_ = 4,326/28,316	Reference *n*_cases/total_ = 677/4,173
Excessive weight gain	1.28 (1.17, 1.41) *n*_cases/total_ = 1,010/14,927	1.36 (1.30, 1.43) *n*_cases/total_ = 4,381/22,400	1.30 (1.14, 1.49) *n*_cases/total_ = 446/2,162
**Overweight (25.0–29.9 kg/m**^**2**^**)**			
Inadequate weight gain	1.49 (1.21, 1.82) *n*_cases/total_ = 118/1,417	1.61 (1.44, 1.81) *n*_cases/total_ = 447/1,767	2.04 (1.52, 2.74) *n*_cases/total_ = 74/239
Adequate weight gain	1.62 (1.41, 1.86) *n*_cases/total_ = 295/3,451	1.87 (1.73, 2.01) *n*_cases/total_ = 1,224/4,656	1.94 (1.58, 2.38) *n*_cases/total_ = 163/574
Excessive weight gain	1.85 (1.68, 2.04) *n*_cases/total_ = 828/9,010	2.25 (2.13, 2.37) *n*_cases/total_ = 3,660/12,786	2.65 (2.28, 3.08) *n*_cases/total_ = 395/1,119
**Obesity (≥30.0 kg/m**^**2**^**)**			
Inadequate weight gain	2.04 (1.66, 2.52) *n*_cases/total_ = 112/1,070	2.95 (2.62, 3.31) *n*_cases/total_ = 509/1,382	5.62 (3.94, 8.02) *n*_cases/total_ = 76/137
Adequate weight gain	2.49 (2.09, 2.97) *n*_cases/total_ = 171/1,378	3.45 (3.12, 3.82) *n*_cases/total_ = 728/1,881	4.64 (3.39, 6.34) *n*_cases/total_ = 88/179
Excessive weight gain	2.63 (2.32, 2.98) *n*_cases/total_ = 402/3,198	3.70 (3.44, 3.97) *n*_cases/total_ = 1,758/4,380	6.02 (4.79, 7.56) *n*_cases/total_ = 207/366

Values are odds ratios (95% confidence intervals) from multilevel binary logistic regression models that reflect the risk of childhood overweight/obesity in early childhood (2.0–5.0 years), mid childhood (5.0–10.0 years), and late childhood (10.0–18.0 years) in children of mothers in the different BMI and gestational weight gain categories, as compared to the reference group (normal weight mothers with adequate gestational weight gain). The models are adjusted for maternal age, education level, ethnicity, parity, and smoking during pregnancy. *p*-Values for interaction between maternal BMI and gestational weight gain for the risk of childhood overweight/obesity: *p =* 0.038, *p <* 0.001, and *p =* 0.637 in early, mid, and late childhood, respectively.

**Table 5:** Combined associations of maternal pre-pregnancy BMI and gestational weight gain clinical categories with childhood BMI standard deviation score (SDS).

Maternal pre-pregnancy BMI	Childhood BMI		
	Early childhood 2.0–5.0 years	Mid childhood 5.0–10.0 years	Late childhood 10.0–18.0 years
**Underweight (<18.5 kg/m**^**2**^**)**			
Inadequate weight gain	−0.32 (−0.39, −0.25) *n =* 991	−0.52 (−0.58, −0.47) *n =* 1,351	−0.53 (−0.65, −0.41) *n =* 280
Adequate weight gain	−0.27 (−0.34, −0.21) *n =* 1,118	−0.37 (−0.42, −0.31) *n =* 1,598	−0.43 (−0.57, −0.30) *n =* 236
Excessive weight gain	−0.14 (−0.24, −0.05) *n =* 469	−0.19 (−0.26, −0.11) *n =* 666	−0.21 (−0.47, 0.04) *n =* 60
**Normal weight (18.5–24.9 kg/m**^**2**^**)**			
Inadequate weight gain	−0.10 (−0.12, −0.07) *n =* 12,264	−0.07 (−0.09, −0.05) *n =* 16,549	−0.06 (−0.11, −0.01) *n =* 3,340
Adequate weight gain	Reference *n =* 19,815	Reference *n =* 28,928	Reference *n =* 4,249
Excessive weight gain	0.10 (0.08, 0.13) *n =* 15,121	0.14 (0.13, 0.16) *n =* 22,825	0.12 (0.06, 0.17) *n =* 2,187
**Overweight (25.0–29.9 kg/m**^**2**^**)**			
Inadequate weight gain	0.06 (0.00, 0.12) *n =* 1,445	0.22 (0.17, 0.27) *n =* 1,803	0.32 (0.19, 0.45) *n =* 241
Adequate weight gain	0.13 (0.09, 0.17) *n =* 3,501	0.29 (0.26, 0.32) *n =* 4,715	0.34 (0.25, 0.43) *n =* 581
Excessive weight gain	0.23 (0.20, 0.25) *n =* 9,137	0.41 (0.39, 0.43) *n =* 12,962	0.55 (0.49, 0.62) *n =* 1,127
**Obesity (≥30.0 kg/m**^**2**^**)**			
Inadequate weight gain	0.26 (0.20, 0.26) *n =* 1,082	0.58 (0.52, 0.63) *n =* 1,391	0.94 (0.77, 1.11) *n =* 137
Adequate weight gain	0.36 (0.30, 0.41) *n =* 1,395	0.64 (0.59, 0.69) *n =* 1,901	0.88 (0.73, 1.03) *n =* 181
Excessive weight gain	0.36 (0.33, 0.40) *n =* 3,238	0.69 (0.66, 0.72) *n =* 4,426	1.01 (0.90, 1.11) *n =* 368

Values are regression coefficients (95% confidence intervals) from multilevel linear regression models that reflect differences in BMI SDS in early childhood (2.0–5.0 years), mid childhood (5.0–10.0 years), and late childhood (10.0–18.0 years) in children of mothers in the different BMI and gestational weight gain groups compared with the reference group (normal weight and adequate gestational weight gain). The models are adjusted for maternal age, education level, ethnicity, parity, and smoking during pregnancy. *p-*Values for interaction between maternal BMI and gestational weight gain for childhood BMI SDS: *p =* 0.016, *p =* 0.002, and *p =* 0.406 in early, mid, and late childhood, respectively.

## Discussion

In this IPD meta-analysis, we observed that higher maternal pre-pregnancy BMI and gestational weight gain were across their full ranges associated with higher risks of offspring overweight/obesity throughout childhood. The effects tended to be stronger at older ages. However, the effect of gestational weight gain in addition to that of pre-pregnancy BMI was small. At the population level, 21.7% to 41.7% of childhood overweight/obesity prevalence was estimated to be attributable to maternal overweight and obesity together, whereas 11.4% to 19.2% was estimated to be attributable to excessive gestational weight gain.

### Interpretation of main findings

Maternal obesity does not only affect pregnancy outcomes, but may also have persistent effects on offspring fat development. Previous studies showed consistently that maternal overweight and obesity were positively associated with offspring BMI [2–4]. In this IPD meta-analysis, we observed not only that maternal overweight and obesity were associated with higher risks of childhood overweight/obesity, but that these risks were progressively higher among children of mothers with class I, class II, and class III obesity, respectively. In addition, we observed that this association was not limited to the extremes of maternal BMI, but was present across the full range. We observed a stronger association of maternal BMI with the risk of childhood overweight/obesity at later ages. Although we used a different reference chart in early childhood and did not correct for correlations between BMI measurements due to the large sample size, the observed association is unlikely to be explained by methodological issues as the observed risk also increased between mid and late childhood, where we used the same reference charts. Also, 2 recent studies among 1,494 Australian and 3,805 Dutch participants observed stronger associations of maternal BMI with childhood growth and obesity risk with increasing age, while accounting for correlated repeated measures [16,17]. This increasing strength of the association with age might reflect an intra-uterine programming mechanism becoming more apparent when children get older, or might be explained by a stronger influence of lifestyle characteristics of the child at later ages. We estimated that about 10% to 20% of overweight/obesity cases throughout childhood are attributable to maternal pre-pregnancy overweight and obesity, with the highest proportions explained by maternal overweight. Thus, our results suggest that high maternal BMI has a considerable population impact, and can be used as a target for preventive strategies. Importantly, the risk of childhood overweight and obesity is not confined to maternal obesity, but increases gradually over the full range of maternal pre-pregnancy BMI.

In addition to pre-pregnancy BMI, the amount of weight gain during pregnancy also seems to be associated with offspring obesity [18,19]. Previous meta-analyses of published studies showed a 33% to 40% increased risk of overweight or obesity in children of mothers with excessive gestational weight gain [6,7]. In line with these studies, we observed that excessive gestational weight gain was related to a 39%–72% higher risk of overweight throughout childhood. On a population level, 11% to 19% of childhood overweight/obesity could be attributed to excessive gestational weight gain. Also, gestational weight gain *z-*scores (specific for maternal BMI and gestational age) across the full range tended to be associated with an increased risk of offspring overweight/obesity. Thus, higher gestational weight gain across the full range, rather than only lower or higher gestational weight gain than recommended by the IOM, seems to be related to offspring weight status.

For the prevention of childhood overweight and obesity, insight into the combined effects of maternal BMI and gestational weight gain is important. Only 2 previous studies assessed the combined associations of maternal pre-pregnancy BMI and gestational weight gain with childhood adiposity [20,21]. A study among 100,612 participants from China reported that, compared to normal maternal weight and adequate weight gain, normal weight or overweight/obesity and excessive gestational weight gain was associated with an increased risk of overweight at 3–6 years of age in children [20]. In a study in the US among 4,436 participants describing trajectories of maternal weight from pre-pregnancy until the postpartum period, the trajectory with the highest risk of offspring obesity at ages 6–11 and 12–19 years consisted almost entirely of women who were overweight or obese at the start of pregnancy, but only half of this group had excessive gestational weight gain [21]. We observed that, compared to children of women with normal weight and adequate gestational weight gain, children of overweight and obese mothers had a higher risk of overweight/obesity, regardless of gestational weight gain. Within the BMI categories, there was only a small effect of gestational weight gain on offspring overweight/obesity. These findings suggest that the effects of gestational weight gain add only to a limited extent to the effects of maternal pre-pregnancy BMI.

Our results strongly suggest that maternal pre-pregnancy BMI and gestational weight gain are associated with increased risk of overweight and obesity throughout childhood. It remains unclear whether these associations are causal and which mechanisms are underlying these associations. Maternal pre-pregnancy obesity and excessive gestational weight gain are complex traits, which reflect multiple components. Maternal pre-pregnancy obesity reflects maternal genetic predisposition, nutritional status, fat accumulation, and low-grade inflammation, whereas maternal weight gain during pregnancy also reflects fluid expansion and growth of the fetus, placenta, and uterus [22,23]. Both may, at least partly, be explained by intra-uterine programming mechanisms. The fetal over-nutrition hypothesis suggests that increased fetal exposure to nutrients may lead to persistent adaptations in the structure and function of adipose tissue, appetite regulation, and energy metabolism, leading to an increased susceptibility to later obesity [24,25]. Epigenetic processes may play an important role in these adaptations [26]. The associations may also reflect genetic predisposition to obesity [27,28] or sociodemographic or lifestyle factors shared by mother and child. Common pregnancy disorders, including gestational diabetes and gestational hypertensive disorders, have also been related to offspring obesity risk [29–31]. However, using data from the same studies as our current analyses, we previously reported that these associations were not independent of maternal BMI [9]. In the current analysis, the associations of maternal BMI with the risk of childhood overweight/obesity were independent of gestational diabetes and gestational hypertensive disorders. Unfortunately, no information on maternal glucose concentrations was available to assess the role of maternal glycemic status in further detail. Previous research has shown that there are sex differences in weight and body fat development in childhood [32,33]. We hypothesized that maternal BMI and gestational weight gain would influence the risk of childhood overweight/obesity differently or with a different timing in boys and girls. However, we did not observe sex differences in the observed associations, possibly due to the fact that BMI does not distinguish between fat and lean mass. Further research is needed into the causality and underlying mechanisms of these associations [22].

Maternal pre-pregnancy BMI and, to a smaller extent, gestational weight gain are important modifiable risk factors of childhood weight status with a considerable population impact. Thus far, intervention trials have been focused on maternal weight during mid or late pregnancy, mostly showing reductions in gestational weight gain, but not showing any effect on birth outcomes or infant body composition [34–36]. Only 1 small Swedish study including a lifestyle intervention reported results on childhood adiposity and showed no difference in BMI at age 5 years [37]. We observed that the effect of excessive gestational weight gain was small in women with pre-pregnancy overweight and obesity. We also observed that the highest proportion of childhood overweight/obesity on a population level could be attributed to maternal pre-pregnancy overweight. Future intervention studies should shift their focus to preconceptional weight management, targeting women of reproductive age to achieve a normal weight.

### Strengths and limitations

We meta-analyzed original data of different pregnancy and birth cohorts, limiting the potential of publication bias and enabling a consistent definition of exposures and outcomes and adjustment for potential confounders. Due to the large sample size, we were able to study effects in people with relatively rare conditions, such as severely obese women. We had data available at different childhood ages, enabling us to study the effects throughout childhood. We did not assess the associations of maternal BMI and gestational weight gain with the risk of childhood overweight/obesity over time in longitudinal analyses, as the data needed for such analyses were only available in a small subgroup of children and cohorts. Further studies are needed specifically exploring the development of childhood overweight and obesity over time in response to maternal BMI and gestational weight gain. Cohorts were initiated between 1989 and 2011. Given the rise in obesity prevalence in the past decades [38], it is likely that the current obesity prevalence and consequently the proportions of childhood overweight/obesity attributable to maternal overweight and obesity are underestimated. In our study, fewer cohorts reached the age for the late childhood analyses than for the early or mid childhood analyses. Our results for the late childhood analyses may be biased by cohort effects. We consider that the bias would most likely lead to an underestimation of the age differences because of the higher prevalences of childhood obesity in more recently started cohorts with younger children. As this study only included cohorts from Europe, North America, and Australia, and demographic characteristics in other continents may be different, results can only be generalized to participants from these continents. Further studies are needed to address similar research questions in other populations. We performed sensitivity analyses based on 2-stage random effects meta-analyses, which gave similar results with low to moderate heterogeneity between the cohorts. We used a missing value category to deal with missing data for covariates. Due to data availability and the size of the dataset, we were not able to apply more advanced imputation strategies. We observed similar results when we conducted a complete case analysis (S7 and S8 Tables). Because maternal BMI was self-reported in some cohorts, some misclassification and underestimation of the effect estimates might be present. For the cohorts with no information on maternal pre-pregnancy BMI available, we used BMI measured in early pregnancy, which might have led to an overestimation of BMI as a result of gestational weight gain. For the country-specific analyses, it is important to note that countries are represented by 1 to 5 cohorts of different sample sizes per country and that not all cohorts are population-based, affecting the representativeness of the results for the full country. Although we adjusted the models for potential confounders, residual confounding by, for example, physical activity and dietary intake might still be an issue.

### Conclusions

Higher maternal pre-pregnancy BMI and gestational weight gain are across their full ranges associated with an increased risk of offspring overweight/obesity throughout childhood and have a considerable population impact. The effect of gestational weight gain in addition to the effect of maternal pre-pregnancy BMI was small. Future intervention trials aiming to reduce childhood overweight and obesity should focus on maternal weight status before pregnancy, in addition to weight gain during pregnancy.

## Supporting information

S1 Fig Country-specific description of exposures and outcomes. (PDF)

S2 Fig Country-specific PARs of maternal overweight, maternal obesity, and excessive gestational weight gain for mid childhood overweight/obesity. (PDF)

S3 Fig Associations of maternal pre-pregnancy BMI with the risk of childhood overweight/obesity assessed by 2-stage IPD meta-analysis. (PDF)

S4 Fig Associations of gestational weight gain with the risk of overweight/obesity assessed by 2-stage IPD meta-analysis. (PDF)

S1 STROBE Statement STROBE checklist. (PDF)

S1 Table Cohort-specific methods of data collection. (PDF)

S2 Table Cohort-specific description of available covariates. (PDF)

S3 Table Associations of maternal pre-pregnancy BMI clinical categories with the risk of childhood overweight/obesity, additionally adjusted for gestational diabetes. (PDF)

S4 Table Associations of maternal pre-pregnancy BMI clinical categories with the risk of childhood overweight/obesity, additionally adjusted for gestational hypertensive disorders. (PDF)

S5 Table Associations of maternal pre-pregnancy BMI clinical categories with the risk of childhood overweight/obesity, additionally adjusted for gestational-age-adjusted birth weight. (PDF)

S6 Table Associations of maternal pre-pregnancy BMI and gestational weight gain clinical categories with the risk of childhood underweight. (PDF)

S7 Table Associations of maternal pre-pregnancy BMI and gestational weight gain clinical categories with the risk of childhood overweight/obesity: Complete case analysis. (PDF)

S8 Table Associations of maternal pre-pregnancy BMI and gestational weight gain clinical categories with childhood BMI SDS: Complete case analysis. (PDF)

S9 Table Contact information for data requests per cohort. (PDF)

S1 Text Plan for analysis for invitation of cohorts. (PDF)

S2 Text Sources of funding/support per cohort. (PDF)

S3 Text Acknowledgments per cohort. (PDF)

## Abbreviations

BMI: body mass index
IOM: US Institute of Medicine
IPD: individual participant data
MOCO: Maternal Obesity and Childhood Outcomes
OR: odds ratio
PAR: population attributable risk fraction
SDS: standard deviation score
WHO: World Health Organization
