# Supplementary material for: Maternal body mass index, gestational weight gain, and the risk of overweight and obesity across childhood: An individual participant data meta-analysis
Source: PLoS Med. 2019 Feb 11;16(2):e1002744. doi: 10.1371/journal.pmed.1002744 (PMC6370184; doi:10.1371/journal.pmed.1002744)
Supplement: S1 Fig — (PDF) [file pmed.1002744.s001.pdf]

S1 Fig. Country-specific description of exposures and outcomes

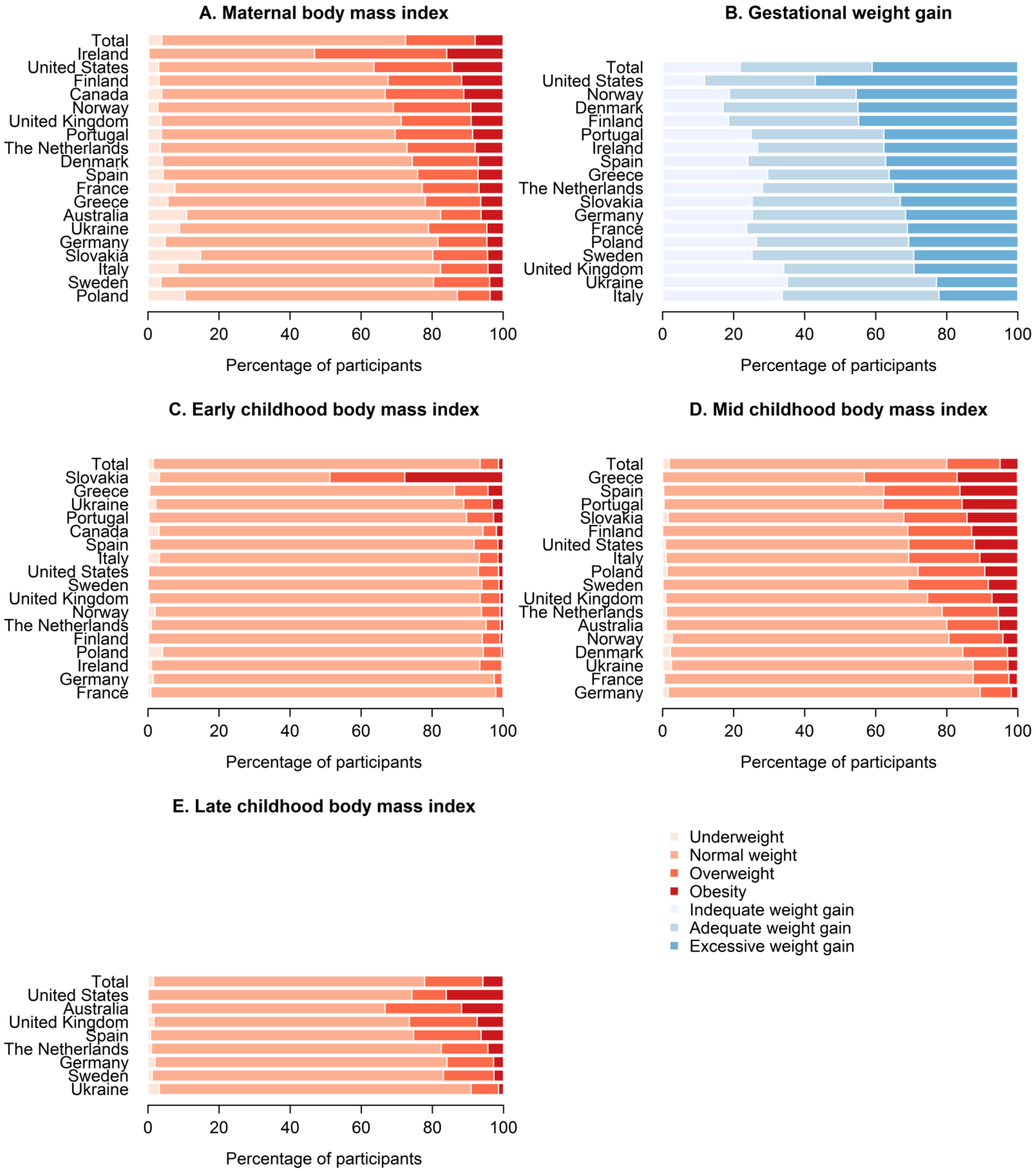

Values are valid percentages. The CHOP cohort was excluded from the country-specific analyses, as participants come from multiple countries.
