# Supplementary material for: Maternal body mass index, gestational weight gain, and the risk of overweight and obesity across childhood: An individual participant data meta-analysis
Source: PLoS Med. 2019 Feb 11;16(2):e1002744. doi: 10.1371/journal.pmed.1002744 (PMC6370184; doi:10.1371/journal.pmed.1002744)
Supplement: S2 Fig — (PDF) [file pmed.1002744.s002.pdf]

S2 Fig. Overall and country-specific population attributable risk fractions of maternal overweight, obesity and excessive gestational weight gain for mid childhood overweight/obesity

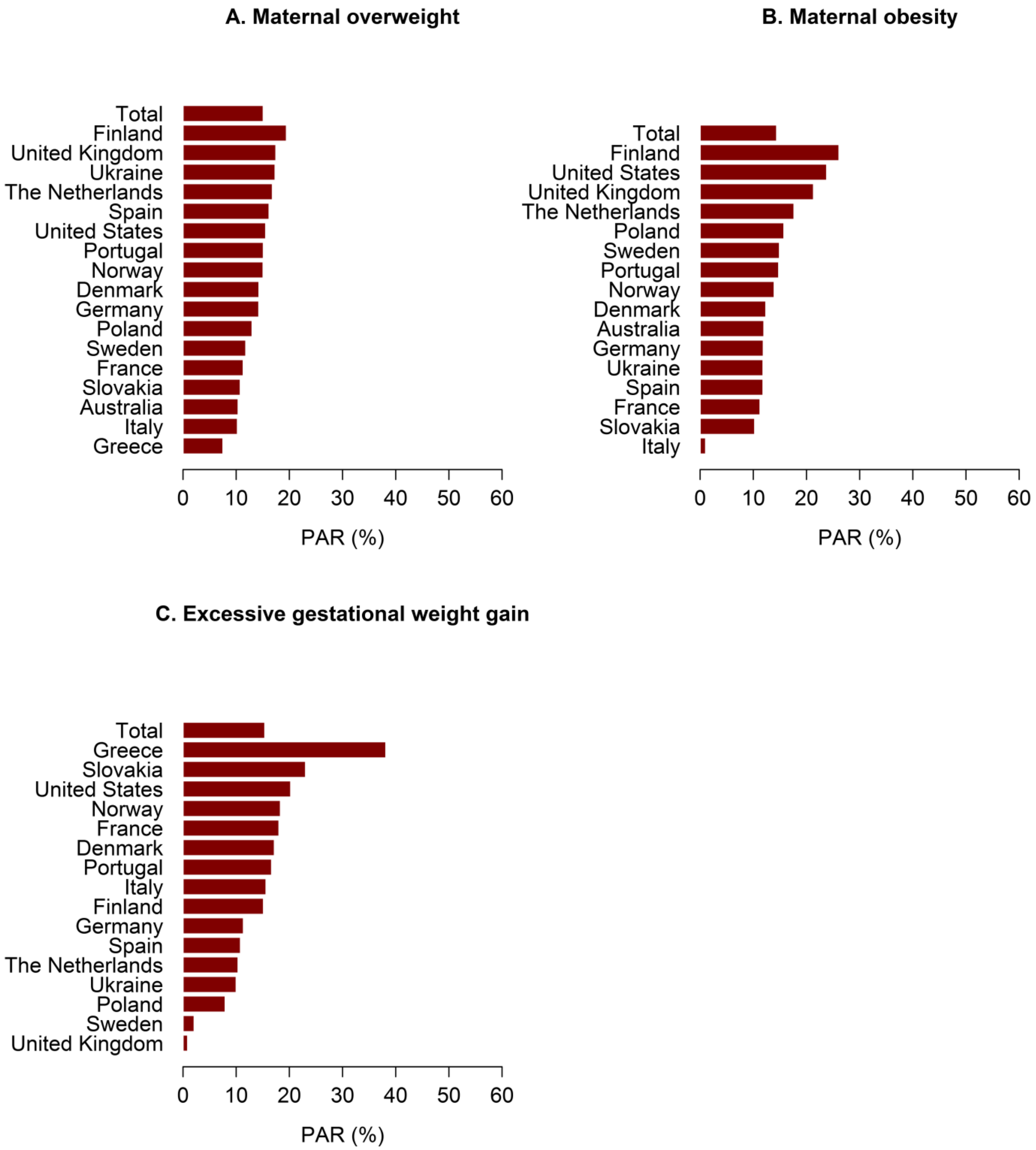

Values are population attributable risk fractions (PAR) indicating the proportion of mid childhood overweight/obesity cases attributable to (A) maternal overweight, (B) maternal obesity and (C) excessive gestational weight gain. The CHOP cohort was excluded from the country-specific analyses, as participants come from multiple countries.
