# Supplementary material for: Maternal body mass index, gestational weight gain, and the risk of overweight and obesity across childhood: An individual participant data meta-analysis
Source: PLoS Med. 2019 Feb 11;16(2):e1002744. doi: 10.1371/journal.pmed.1002744 (PMC6370184; doi:10.1371/journal.pmed.1002744)
Supplement: S3 Fig — (PDF) [file pmed.1002744.s003.pdf]

S3 Fig. Associations of maternal pre-pregnancy BMI with the risk of childhood overweight/obesity assessed by 2-stage IPD meta-analysis

A. Early childhood

Study or Subgroup	Weight	Odds Ratio IV, Random, 95% CI
ABCD (The Netherlands)	4.8%	1.12 [1.09, 1.15]
ALSPAC (United Kingdom)	3.4%	1.08 [1.03, 1.14]
AOB/F (Canada)	4.2%	1.06 [1.02, 1.09]
BAMSE (Sweden)	3.7%	1.15 [1.10, 1.20]
BIB (United Kingdom)	3.8%	1.09 [1.04, 1.13]
CHOP (Multiple)	1.7%	1.11 [1.02, 1.21]
Co.N.ER (Italy)	1.7%	1.09 [1.00, 1.18]
DNBC (Denmark)		Not estimable
EDEN (France)	2.1%	1.09 [1.01, 1.17]
FCOU (Ukraine)	3.2%	1.03 [0.98, 1.08]
GASPII (Italy)	1.8%	1.08 [1.00, 1.17]
GECKO Drenthe (The Netherlands)		Not estimable
GENERATION R (The Netherlands)	4.7%	1.12 [1.09, 1.15]
GENERATION XXI (Portugal)	5.4%	1.13 [1.11, 1.15]
GENESIS (Greece)	4.1%	1.06 [1.02, 1.10]
GINIplus (Germany)	2.6%	1.14 [1.07, 1.21]
HUMIS (Norway)	2.3%	1.02 [0.95, 1.09]
INMA (Spain)	4.4%	1.13 [1.09, 1.16]
KOALA (The Netherlands)	0.8%	1.02 [0.89, 1.18]
Krakow cohort (Poland)	0.4%	1.24 [1.02, 1.51]
LISAplus (Germany)	3.2%	1.12 [1.06, 1.18]
LUKAS (Finland)	2.0%	1.19 [1.10, 1.28]
MoBa (Norway)	6.0%	1.06 [1.06, 1.07]
NINFEA (Italy)	3.4%	1.09 [1.04, 1.15]
PIAMA (The Netherlands)	3.0%	1.11 [1.05, 1.17]
Piccolipiù (Italy)	1.9%	1.04 [0.96, 1.13]
Project Viva (United States)	4.1%	1.09 [1.05, 1.13]
PÉLAGIE (France)	0.7%	0.97 [0.83, 1.13]
Raine Study (Australia)		Not estimable
REPRO_PL (Poland)	1.1%	1.18 [1.05, 1.32]
RHEA (Greece)	3.8%	1.07 [1.02, 1.11]
ROLO (Ireland)	1.5%	1.11 [1.01, 1.21]
SCOPE BASELINE (Ireland)	2.6%	1.05 [0.99, 1.11]
SEATON (United Kingdom)	2.5%	1.10 [1.04, 1.18]
Slovak PCB study (Slovakia)	3.0%	1.01 [0.95, 1.06]
STEPS (Finland)	1.6%	1.04 [0.95, 1.13]
SWS (United Kingdom)	4.7%	1.08 [1.05, 1.11]
**Total (95% CI)**	**100.0%**	**1.09 [1.07, 1.10]**

Heterogeneity: Tau² = 0.00; Chi² = 98.43, df = 33 (P < 0.00001); I² = 66%
Test for overall effect: Z = 12.76 (P < 0.00001)

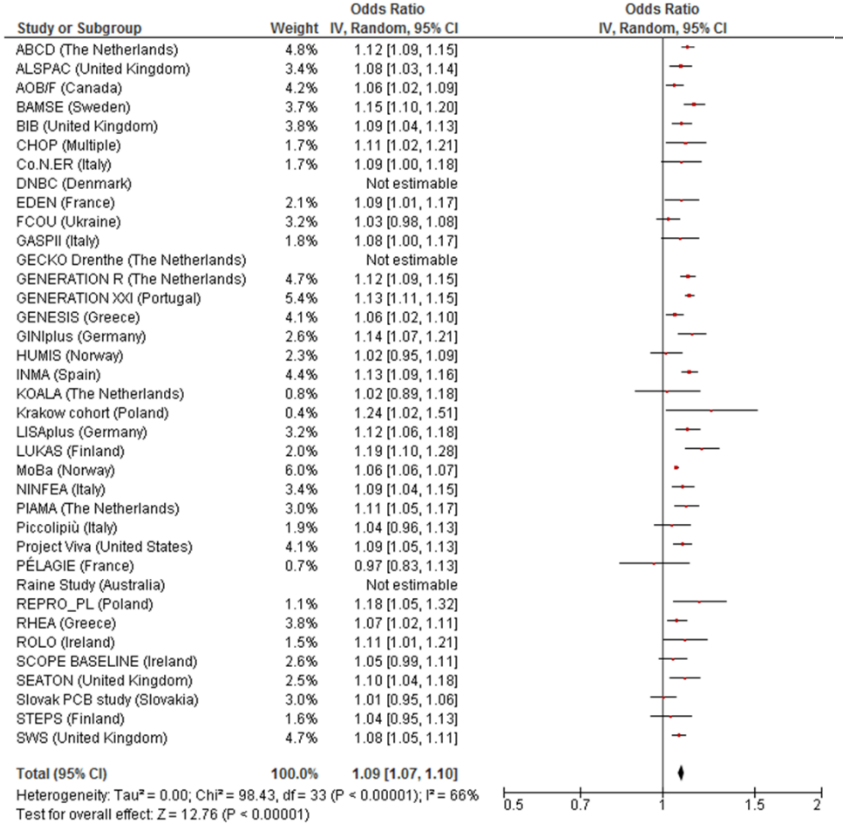

B. Mid childhood

Study or Subgroup	Weight	Odds Ratio IV, Random, 95% CI
ABCD (The Netherlands)	6.1%	1.11 [1.09, 1.13]
ALSPAC (United Kingdom)	7.1%	1.14 [1.12, 1.15]
AOB/F (Canada)		Not estimable
BAMSE (Sweden)	3.8%	1.18 [1.15, 1.22]
BIB (United Kingdom)		Not estimable
CHOP (Multiple)	2.6%	1.13 [1.09, 1.18]
Co.N.ER (Italy)	0.7%	1.14 [1.05, 1.24]
DNBC (Denmark)	8.9%	1.12 [1.11, 1.12]
EDEN (France)	2.8%	1.10 [1.06, 1.14]
FCOU (Ukraine)	1.7%	1.10 [1.05, 1.16]
GASPII (Italy)	1.3%	1.07 [1.01, 1.13]
GECKO Drenthe (The Netherlands)	5.2%	1.11 [1.08, 1.13]
GENERATION R (The Netherlands)	7.1%	1.13 [1.12, 1.15]
GENERATION XXI (Portugal)	7.1%	1.12 [1.11, 1.14]
GENESIS (Greece)	0.2%	1.08 [0.91, 1.29]
GINIplus (Germany)	3.0%	1.16 [1.12, 1.20]
HUMIS (Norway)	1.2%	1.09 [1.03, 1.16]
INMA (Spain)	3.8%	1.12 [1.09, 1.15]
KOALA (The Netherlands)	3.0%	1.12 [1.08, 1.16]
Krakow cohort (Poland)	0.6%	1.17 [1.06, 1.28]
LISAplus (Germany)	3.2%	1.11 [1.07, 1.15]
LUKAS (Finland)	1.6%	1.14 [1.08, 1.20]
MoBa (Norway)	8.9%	1.10 [1.10, 1.11]
NINFEA (Italy)	1.1%	1.14 [1.07, 1.22]
PIAMA (The Netherlands)	3.2%	1.19 [1.15, 1.23]
Piccolipiù (Italy)		Not estimable
Project Viva (United States)	4.1%	1.10 [1.07, 1.13]
PÉLAGIE (France)		Not estimable
Raine Study (Australia)	4.4%	1.11 [1.09, 1.14]
REPRO_PL (Poland)	0.1%	1.31 [1.02, 1.68]
RHEA (Greece)		Not estimable
ROLO (Ireland)		Not estimable
SCOPE BASELINE (Ireland)		Not estimable
SEATON (United Kingdom)	1.3%	1.11 [1.05, 1.18]
Slovak PCB study (Slovakia)	1.2%	1.12 [1.05, 1.19]
STEPS (Finland)		Not estimable
SWS (United Kingdom)	4.8%	1.13 [1.10, 1.15]
**Total (95% CI)**	**100.0%**	**1.12 [1.11, 1.13]**

Heterogeneity: Tau² = 0.00; Chi² = 70.58, df = 28 (P < 0.0001); I² = 60%
Test for overall effect: Z = 30.69 (P < 0.00001)

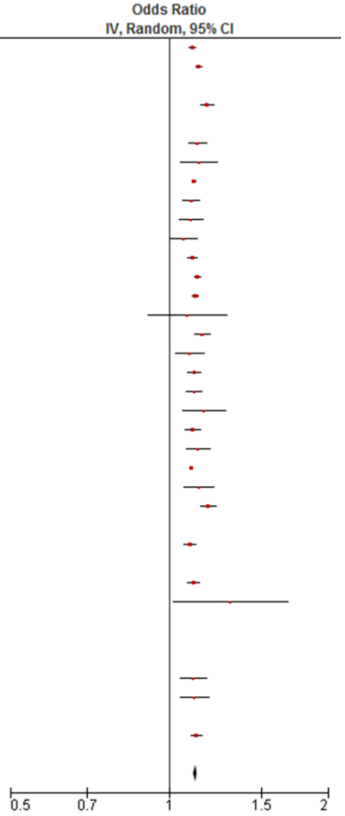

S3 Fig. Associations of maternal pre-pregnancy BMI with the risk of childhood overweight/obesity assessed by 2-stage IPD meta-analysis (continued)

C. Late childhood

Study or Subgroup	Weight	Odds Ratio IV, Random, 95% CI
ABCD (The Netherlands)		Not estimable
ALSPAC (United Kingdom)	23.3%	1.17 [1.15, 1.18]
AOB/F (Canada)		Not estimable
BAMSE (Sweden)	11.3%	1.18 [1.15, 1.22]
BIB (United Kingdom)		Not estimable
CHOP (Multiple)		Not estimable
Co.N.ER (Italy)		Not estimable
DNBC (Denmark)		Not estimable
EDEN (France)		Not estimable
FCOU (Ukraine)	4.1%	1.17 [1.10, 1.25]
GASPII (Italy)		Not estimable
GECKO Drenthe (The Netherlands)		Not estimable
GENERATION R (The Netherlands)	6.1%	1.13 [1.07, 1.19]
GENERATION XXI (Portugal)		Not estimable
GENESIS (Greece)		Not estimable
GINIplus (Germany)	12.1%	1.16 [1.12, 1.20]
HUMIS (Norway)		Not estimable
INMA (Spain)	2.3%	1.20 [1.10, 1.31]
KOALA (The Netherlands)	1.3%	1.18 [1.04, 1.33]
Krakow cohort (Poland)		Not estimable
LISAplus (Germany)	12.1%	1.17 [1.13, 1.20]
LUKAS (Finland)		Not estimable
MoBa (Norway)		Not estimable
NINFEA (Italy)		Not estimable
PIAMA (The Netherlands)	3.6%	1.23 [1.15, 1.32]
Piccolipiù (Italy)		Not estimable
Project Viva (United States)		Not estimable
PÉLAGIE (France)		Not estimable
Raine Study (Australia)	14.1%	1.13 [1.10, 1.16]
REPRO_PL (Poland)		Not estimable
RHEA (Greece)		Not estimable
ROLO (Ireland)		Not estimable
SCOPE BASELINE (Ireland)		Not estimable
SEATON (United Kingdom)	9.7%	1.11 [1.07, 1.15]
Slovak PCB study (Slovakia)		Not estimable
STEPS (Finland)		Not estimable
SWS (United Kingdom)		Not estimable
**Total (95% CI)**	**100.0%**	**1.16 [1.14, 1.17]**

Heterogeneity: $Tau^2 = 0.00$; $Chi^2 = 15.61$, df = 10 (P = 0.11); $I^2 = 36\%$
Test for overall effect: Z = 20.25 (P < 0.00001)

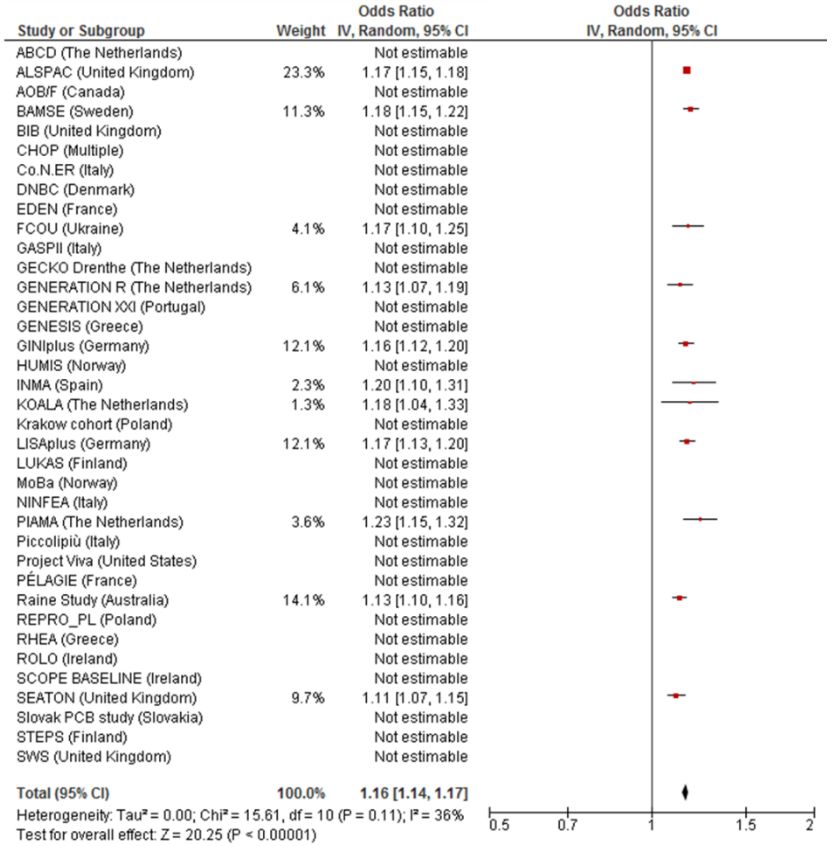

Values are pooled odds ratios (95% confidence intervals) that reflect the risk of childhood overweight/obesity in (A) early childhood (2.0-5.0 years), (B) mid childhood (5.0-10.0 years) and (C) late childhood (10.0-18.0 years) per kg/m^2 increase in maternal pre-pregnancy BMI. The cohorts for which no estimate was provided had no or not sufficient data available for that particular analysis. The models are adjusted for maternal age, education level, ethnicity, parity, and smoking during pregnancy.
