# Supplementary material for: Maternal body mass index, gestational weight gain, and the risk of overweight and obesity across childhood: An individual participant data meta-analysis
Source: PLoS Med. 2019 Feb 11;16(2):e1002744. doi: 10.1371/journal.pmed.1002744 (PMC6370184; doi:10.1371/journal.pmed.1002744)
Supplement: S4 Fig — (PDF) [file pmed.1002744.s004.pdf]

S4 Fig. Associations of gestational weight gain with the risk of overweight/obesity assessed by 2-stage IPD meta-analysis

A. Early childhood

Study or Subgroup	Weight	Odds Ratio IV, Random, 95% CI
ALSPAC (United Kingdom)	2.7%	1.28 [0.99, 1.66]
BAMSE (Sweden)	0.4%	1.73 [0.87, 3.48]
BIB (United Kingdom)	1.6%	0.90 [0.64, 1.28]
Co.N.ER (Italy)	1.6%	1.02 [0.72, 1.44]
DNBC (Denmark)		Not estimable
EDEN (France)	1.2%	0.97 [0.64, 1.47]
FCOU (Ukraine)	4.5%	1.10 [0.91, 1.33]
GASPII (Italy)	1.7%	1.63 [1.17, 2.27]
GECKO Drenthe (The Netherlands)		Not estimable
GENERATION R (The Netherlands)	6.9%	1.18 [1.03, 1.36]
GENERATION XXI (Portugal)	10.9%	1.20 [1.10, 1.31]
GENESIS (Greece)	9.8%	1.02 [0.92, 1.12]
GINIplus (Germany)	2.5%	1.18 [0.90, 1.55]
HUMIS (Norway)	2.5%	1.09 [0.83, 1.43]
INMA (Spain)	4.0%	1.34 [1.09, 1.64]
KOALA (The Netherlands)	0.7%	2.02 [1.18, 3.46]
Krakow cohort (Poland)	0.6%	1.08 [0.60, 1.95]
LISAplus (Germany)	2.4%	1.13 [0.86, 1.49]
LUKAS (Finland)	1.0%	1.36 [0.86, 2.13]
MoBa (Norway)	15.7%	1.14 [1.10, 1.18]
NINFEA (Italy)	3.5%	1.02 [0.82, 1.27]
PIAMA (The Netherlands)	4.3%	1.34 [1.10, 1.62]
Piccolipiù (Italy)	1.4%	1.16 [0.80, 1.70]
Project Viva (United States)	3.2%	1.35 [1.06, 1.70]
REPRO_PL (Poland)	0.7%	1.51 [0.87, 2.60]
RHEA (Greece)	3.6%	1.17 [0.94, 1.45]
ROLO (Ireland)	0.5%	2.29 [1.19, 4.39]
SCOPE BASELINE (Ireland)	1.2%	1.17 [0.78, 1.76]
Slovak PCB study (Slovakia)	4.2%	0.93 [0.77, 1.13]
STEPS (Finland)	1.0%	1.03 [0.66, 1.60]
SWS (United Kingdom)	5.8%	1.21 [1.03, 1.41]
**Total (95% CI)**	**100.0%**	**1.16 [1.11, 1.22]**

Heterogeneity: Tau² = 0.00; Chi² = 39.26, df = 28 (P = 0.08); I² = 29%
Test for overall effect: Z = 6.38 (P < 0.00001)

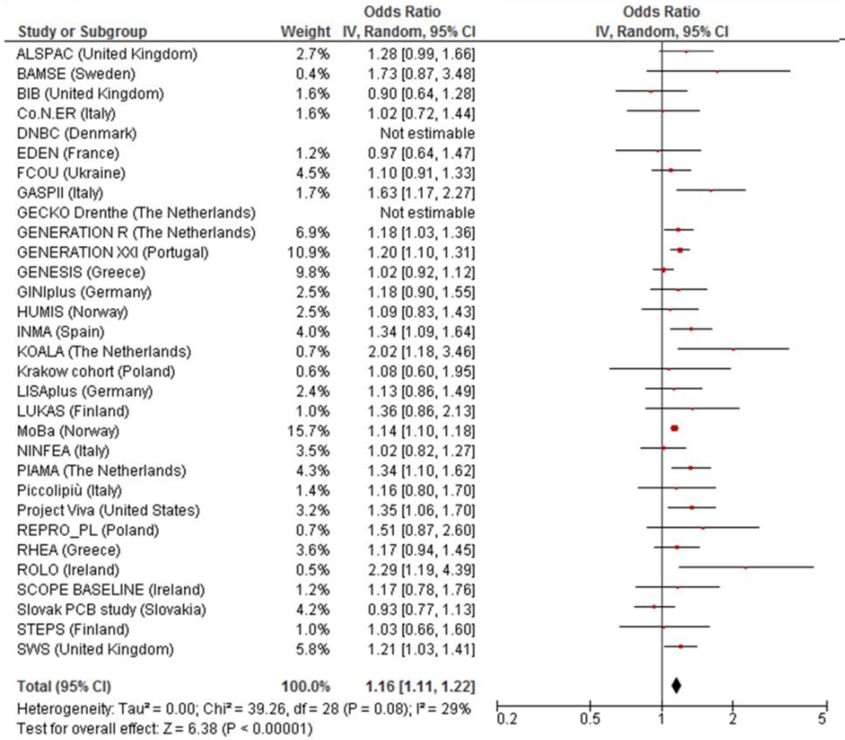

B. Mid childhood

Study or Subgroup	Weight	Odds Ratio IV, Random, 95% CI
ALSPAC (United Kingdom)	7.2%	1.15 [1.09, 1.22]
BAMSE (Sweden)	0.2%	1.37 [0.97, 1.94]
BIB (United Kingdom)		Not estimable
Co.N.ER (Italy)	0.3%	1.20 [0.88, 1.62]
DNBC (Denmark)	26.8%	1.16 [1.13, 1.20]
EDEN (France)	0.7%	1.24 [1.03, 1.50]
FCOU (Ukraine)	0.4%	1.13 [0.90, 1.43]
GASPII (Italy)	0.4%	1.14 [0.91, 1.44]
GECKO Drenthe (The Netherlands)	1.6%	1.30 [1.15, 1.46]
GENERATION R (The Netherlands)	7.2%	1.12 [1.06, 1.18]
GENERATION XXI (Portugal)	8.3%	1.20 [1.14, 1.27]
GENESIS (Greece)	0.2%	1.14 [0.79, 1.65]
GINIplus (Germany)	1.1%	1.07 [0.93, 1.24]
HUMIS (Norway)	0.3%	1.07 [0.80, 1.43]
INMA (Spain)	1.0%	1.30 [1.12, 1.51]
KOALA (The Netherlands)	0.9%	1.16 [0.99, 1.36]
Krakow cohort (Poland)	0.4%	1.18 [0.92, 1.50]
LISAplus (Germany)	1.0%	1.25 [1.07, 1.46]
LUKAS (Finland)	0.4%	1.15 [0.89, 1.48]
MoBa (Norway)	35.7%	1.17 [1.14, 1.20]
NINFEA (Italy)	0.4%	1.18 [0.93, 1.50]
PIAMA (The Netherlands)	2.1%	1.11 [0.99, 1.23]
Piccolipiù (Italy)		Not estimable
Project Viva (United States)	1.1%	1.13 [0.98, 1.31]
REPRO_PL (Poland)	0.0%	0.35 [0.12, 1.02]
RHEA (Greece)		Not estimable
ROLO (Ireland)		Not estimable
SCOPE BASELINE (Ireland)		Not estimable
Slovak PCB study (Slovakia)	0.4%	1.19 [0.93, 1.53]
STEPS (Finland)		Not estimable
SWS (United Kingdom)	2.1%	1.13 [1.01, 1.25]
**Total (95% CI)**	**100.0%**	**1.16 [1.15, 1.18]**

Heterogeneity: Tau² = 0.00; Chi² = 18.89, df = 24 (P = 0.76); I² = 0%
Test for overall effect: Z = 19.52 (P < 0.00001)

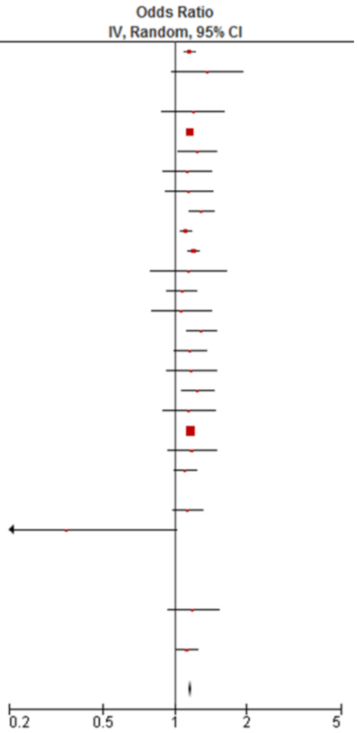

S4 Fig. Associations of maternal gestational weight gain with the risk of childhood overweight/obesity assessed by 2-stage IPD meta-analysis (continued)

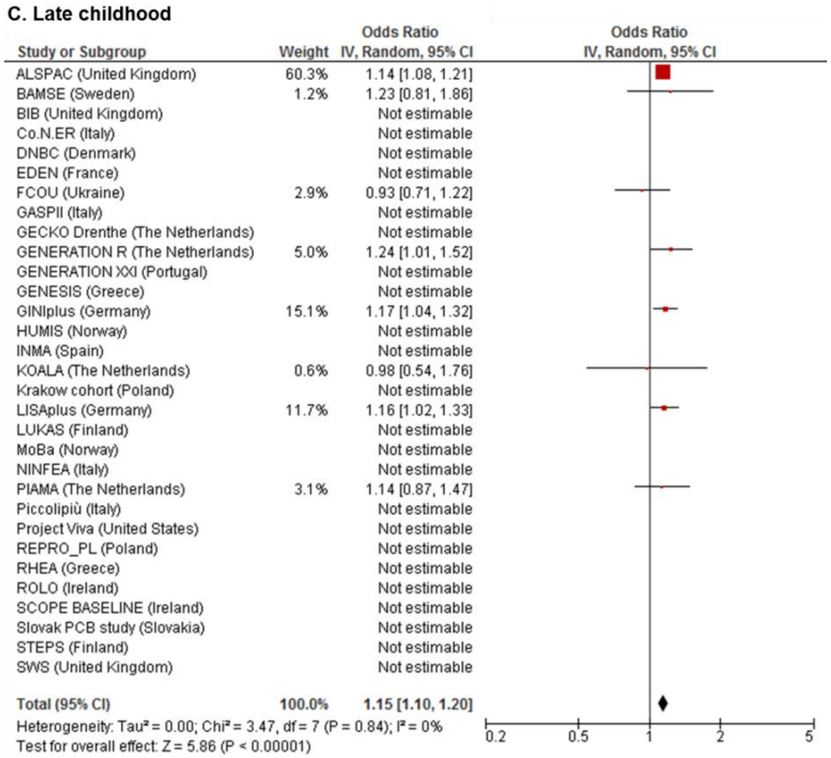

Values are pooled odds ratios (95% confidence intervals) that reflect the risk of childhood overweight/obesity in (A) early childhood (2.0-5.0 years), (B) mid childhood (5.0-10.0 years) and (C) late childhood (10.0-18.0 years) per SD increase in gestational weight gain. The cohorts for which no estimate was provided had no or not sufficient data available for that particular analysis. The models are adjusted for maternal age, education level, ethnicity, parity, and smoking during pregnancy.
