# Supplementary material for: Maternal body mass index, gestational weight gain, and the risk of overweight and obesity across childhood: An individual participant data meta-analysis
Source: PLoS Med. 2019 Feb 11;16(2):e1002744. doi: 10.1371/journal.pmed.1002744 (PMC6370184; doi:10.1371/journal.pmed.1002744)
Supplement: S1 STROBE Statement — (PDF) [file pmed.1002744.s005.pdf]

STROBE Statement—Checklist of items that should be included in reports of ***cohort studies***

	Item No	Recommendation	Section	Paragraph
**Title and abstract**	1	(*a*) Indicate the study's design with a commonly used term in the title or the abstract	Title page	NA
		(*b*) Provide in the abstract an informative and balanced summary of what was done and what was found	Abstract	NA
**Introduction**				
Background/rationale	2	Explain the scientific background and rationale for the investigation being reported	Introduction	- First paragraph
Objectives	3	State specific objectives, including any prespecified hypotheses	Introduction	- Second paragraph
**Methods**				
Study design	4	Present key elements of study design early in the paper	Methods	- Inclusion criteria and participating cohorts
Setting	5	Describe the setting, locations, and relevant dates, including periods of recruitment, exposure, follow-up, and data collection	Methods	- Inclusion criteria and participating cohorts
Participants	6	(*a*) Give the eligibility criteria, and the sources and methods of selection of participants. Describe methods of follow-up	Methods	- Inclusion criteria and participating cohorts
		(*b*) For matched studies, give matching criteria and number of exposed and unexposed	NA	NA
Variables	7	Clearly define all outcomes, exposures, predictors, potential confounders, and effect modifiers. Give diagnostic criteria, if applicable	Methods	- Maternal pre-pregnancy BMI and gestational weight gain<br>- Childhood overweight/obesity<br>- Covariates
Data sources/ measurement	8*	For each variable of interest, give sources of data and details of methods of assessment (measurement). Describe comparability of assessment methods if there is more than one group	Methods	- Maternal pre-pregnancy BMI and gestational weight gain<br>- Childhood overweight/obesity<br>- Covariates
Bias	9	Describe any efforts to address potential sources of bias	Methods	- Statistical analysis
Study size	10	Explain how the study size was arrived at	Methods	- Inclusion criteria and participating cohorts
Quantitative variables	11	Explain how quantitative variables were handled in the analyses. If applicable, describe which groupings were chosen and why	Methods	- Maternal pre-pregnancy BMI and gestational weight gain<br>- Childhood overweight/obesity

				- Covariates<br>- Statistical analysis
Statistical methods	12	(*a*) Describe all statistical methods, including those used to control for confounding	Methods	- Statistical analysis
		(*b*) Describe any methods used to examine subgroups and interactions	Methods	- Statistical analysis
		(*c*) Explain how missing data were addressed	Methods	- Statistical analysis
		(*d*) If applicable, explain how loss to follow-up was addressed	NA	NA
		(*e*) Describe any sensitivity analyses	Methods	- Statistical analysis
**Results**				
Participants	13*	(a) Report numbers of individuals at each stage of study—eg numbers potentially eligible, examined for eligibility, confirmed eligible, included in the study, completing follow-up, and analysed	Methods	- Inclusion criteria and participating cohorts
		(b) Give reasons for non-participation at each stage	Methods	- Inclusion criteria and participating cohorts
		(c) Consider use of a flow diagram	Methods	- Inclusion criteria and participating cohorts
Descriptive data	14*	(a) Give characteristics of study participants (eg demographic, clinical, social) and information on exposures and potential confounders	Results	- Participants' characteristics
		(b) Indicate number of participants with missing data for each variable of interest	Results	- Participants' characteristics
		(c) Summarise follow-up time (eg, average and total amount)	Results	- Table 1
Outcome data	15*	Report numbers of outcome events or summary measures over time	Results	- Participants' characteristics
Main results	16	(*a*) Give unadjusted estimates and, if applicable, confounder-adjusted estimates and their precision (eg, 95% confidence interval). Make clear which confounders were adjusted for and why they were included	Results	- Maternal pre-pregnancy BMI and gestational weight gain clinical categories<br>- Maternal pre-pregnancy BMI and gestational weight gain across the full range<br>- Combined effects of maternal pre-pregnancy BMI and gestational weight gain clinical categories
		(*b*) Report category boundaries when continuous variables were categorized	Methods	- Maternal pre-pregnancy BMI and gestational weight gain<br>- Childhood overweight/obesity<br>- Covariates

		(*c*) If relevant, consider translating estimates of relative risk into absolute risk for a meaningful time period	NA	NA
Other analyses	17	Report other analyses done—eg analyses of subgroups and interactions, and sensitivity analyses	Results	- Maternal pre-pregnancy BMI and gestational weight gain clinical categories<br>- Maternal pre-pregnancy BMI and gestational weight gain across the full range<br>- Combined effects of maternal pre-pregnancy BMI and gestational weight gain clinical categories
**Discussion**				
Key results	18	Summarise key results with reference to study objectives	Discussion	- First paragraph
Limitations	19	Discuss limitations of the study, taking into account sources of potential bias or imprecision. Discuss both direction and magnitude of any potential bias	Discussion	- Strengths and limitations
Interpretation	20	Give a cautious overall interpretation of results considering objectives, limitations, multiplicity of analyses, results from similar studies, and other relevant evidence	Discussion	- Interpretation of main findings
Generalisability	21	Discuss the generalisability (external validity) of the study results	Discussion	- Strengths and limitations
**Other information**				
Funding	22	Give the source of funding and the role of the funders for the present study and, if applicable, for the original study on which the present article is based	Funding/support (per cohort)	NA

*Give information separately for exposed and unexposed groups.

Note: An Explanation and Elaboration article discusses each checklist item and gives methodological background and published examples of transparent reporting. The STROBE checklist is best used in conjunction with this article (freely available on the Web sites of PLoS Medicine at http://www.plosmedicine.org/, Annals of Internal Medicine at http://www.annals.org/, and Epidemiology at http://www.epidem.com/). Information on the STROBE Initiative is available at http://www.strobe-statement.org.
