# Supplementary material for: Maternal body mass index, gestational weight gain, and the risk of overweight and obesity across childhood: An individual participant data meta-analysis
Source: PLoS Med. 2019 Feb 11;16(2):e1002744. doi: 10.1371/journal.pmed.1002744 (PMC6370184; doi:10.1371/journal.pmed.1002744)
Supplement: S1 Table — (PDF) [file pmed.1002744.s006.pdf]

S1 Table. Cohort-specific methods of data collection

Cohort name (country)	Maternal height	Maternal pre-/early pregnancy weight	Maternal latest weight before delivery or gestational weight gain	Childhood weight and height
ABCD (The Netherlands)	Self-reported	Self-reported	NA	Measured
ALSPAC (United Kingdom)	Self-reported	Self-reported	Clinical records	Measured
AOB/F (Canada)	Self-reported	Self-reported	NA	Reported
BAMSE (Sweden)	Medical Birth Registry	Medical Birth Registry	Medical Birth Registry	Measured
BIB (United Kingdom)	Measured	Measured	Clinical records	Measured
CHOP (Multiple)	Measured	Self-reported	NA	Measured
Co.N.ER (Italy)	Self-reported	Self-reported	Self-reported	Reported
DNBC (Denmark)	Self-reported	Self-reported	Self-reported	Reported or measured
EDEN (France)	Measured	Self-reported	Clinical records	Measured or clinical records
FCOU (Ukraine)	Clinical records	Clinical records	Clinical records	Clinical records
GASPII (Italy)	Self-reported	Self-reported	Self-reported	Measured
GECKO Drenthe (The Netherlands)	Self-reported	Self-reported	Self-reported	Measured
GENERATION R (The Netherlands)	Measured	Self-reported	Self-reported	Measured
GENERATION XXI (Portugal)	Measured or ID card	Self-reported	Self-reported	Measured
GENESIS (Greece)	Self-reported	Self-reported	Self-reported	Measured
GINIplus (Germany)	Self-reported	Self-reported	Self-reported	Clinical records at 4y, measured and reported at 10 and 15y
HUMIS (Norway)	Self-reported	Self-reported	Self-reported	Reported
INMA (Spain)	Measured or self-reported	Self-reported	Clinical records	Measured
KOALA (The Netherlands)	Self-reported	Self-reported	Self-reported	Reported

S1 Table. Cohort-specific methods of data collection (continued)

**Cohort name (country)**	**Maternal height**	**Maternal pre-/early pregnancy weight**	**Maternal latest weight before delivery or gestational weight gain**	**Childhood height and weight**
Krakow Cohort (Poland)	Self-reported	Self-reported	Self-reported	Measured
LISAplus (Germany)	Self-reported	Self-reported	Self-reported	Clinical records at 4y, measured and reported at 10 and 15y
LUKAS (Finland)	Self-reported	Self-reported	Self-reported or clinical records	Reported
MoBa (Norway)	Self-reported	Self-reported	Self-reported	Reported
NINFEA (Italy)	Self-reported	Self-reported	Self-reported	Reported
PÉLAGIE (France)	Self-reported	Self-reported	NA	Reported
PIAMA (The Netherlands)	Self-reported	Self-reported	Self-reported	Reported and measured (4 and 8y)
Piccolipiù (Italy)	Self-reported	Self-reported	Self-reported	Measured
Project Viva (United States)	Self-reported	Self-reported	Clinical records	Measured
Raine Study (Australia)	Measured	Self-reported	NA	Measured
REPRO_PL (Poland)	Measured	Self-reported	Measured	Measured
RHEA (Greece)	Measured	Self-reported	Measured	Clinical records or measured
ROLO (Ireland)	Measured	Measured	Measured	Measured
SCOPE BASELINE (Ireland)	Measured	Measured	Measured	Measured
SEATON (United Kingdom)	Measured	Measured	NA	Measured
Slovak PCB study (Slovakia)	Self-reported	Self-reported	Self-reported	Measured
STEPS (Finland)	Self-reported	Self-reported	Self-reported	Measured
SWS (United Kingdom)	Measured	Measured	Measured	Measured

NA: Not available or not applicable.
