# Supplementary material for: Maternal body mass index, gestational weight gain, and the risk of overweight and obesity across childhood: An individual participant data meta-analysis
Source: PLoS Med. 2019 Feb 11;16(2):e1002744. doi: 10.1371/journal.pmed.1002744 (PMC6370184; doi:10.1371/journal.pmed.1002744)
Supplement: S2 Table — (PDF) [file pmed.1002744.s007.pdf]

S2 Table. Cohort-specific description of available covariates

Cohort name, number of participants, (country)	Maternal age, (years)	Parity, n (%)		Maternal education level, n (%)				Ethnicity, n (%)		Smoking during pregnancy, n (%)		Child's sex, n (%)	
	Median (95% range)	Nulliparous	Missings	Low	Medium	High	Missings	European/ White	Missings	Yes	Missings	Male	Missings
ABCD , n=5,494, 2003-2004 (The Netherlands)	32.0 (20.0, 40.0)	3,030 (55.2)	−	1,144 (20.8)	2,018 (36.7)	2,291 (41.7)	41 (0.7)	4,083 (74.3)	10 (0.2)	602 (11.0)	269 (4.9)	2,727 (49.6)	−
ALSPAC, n=8,435, 1991-1992 (United Kingdom)	29.0 (20.0, 38.0)	3,761 (44.6)	217 (2.6)	4,853 (57.5)	2,101 (24.9)	1,239 (14.7)	242 (2.9)	8,018 (95.1)	262 (3.1)	1,701 (20.2)	99 (1.2)	4,266 (50.6)	−
AOB/F, n=1,653, 2008-2010 (Canada)	31.0 (22.0, 40.0)	832 (50.3)	−	131 (7.9)	1,248 (75.5)	271 (16.4)	3 (0.2)	1,356 (82.0)	2 (0.1)	143 (8.7)	89 (5.4)	867 (53.0)	−
BAMSE, n=2,930, 1994-1996 (Sweden)	30.0 (22.0, 40.0)	1,641 (56.0)	−	981 (33.5)	747 (25.5)	1,185 (40.4)	17 (0.6)	2,549 (87.0)	212 (7.2)	372 (12.7)	−	1,477 (50.4)	−
BIB, n=887, 2007-2010 (United Kingdom)	27.0 (18.0, 39.0)	349 (39.3)	15 (1.7)	211 (23.8)	340 (38.3)	334 (37.7)	2 (0.2)	368 (41.5)	-	130 (14.7)	1 (0.1)	413 (46.6)	−
CHOP, n=905, 2002-2004 (Multiple)	30.7 (20.3, 39.9)	451 (49.8)	2 (0.2)	192 (21.2)	460 (50.8)	251 (27.7)	2 (0.2)	NA	−	208 (23.0)	0 (0.2)	429 (47.4)	−
Co.N.ER, n=522, 2004-2005 (Italy)	33.9 (25.5, 42.1)	234 (44.8)	1 (0.2)	79 (15.1)	237 (45.5)	205 (39.3)	1 (0.2)	518 (99.2)	−	66 (12.6)	−	264 (50.6)	−
DNBC, n=39,637, 1996-2002 (Denmark)	30.4 (22.9, 39.4)	19,601 (49.5)	22 (0.1)	3,039 (7.7)	14,410 (36.4)	22,071 (55.7)	117 (0.3)	NA	−	9,432 (23.8)	14 (0)	20,396 (51.5)	−
EDEN, n=1,331, 2003-2005 (France)	29.9 (21.2, 39.7)	709 (53.3)	2 (0.2)	305 (22.9)	246 (18.5)	777 (58.4)	3 (0.2)	NA	−	302 (22.7)	6 (0.5)	705 (53.0)	−
FCOU, n=2,107, 1993-1996 (Ukraine)	23.0 (17.0, 36.0)	1,401 (66.5)	75 (3.6)	107 (5.1)	1,443 (68.5)	518 (24.6)	39 (1.9)	2,107 (100.0)	−	167 (7.9)	128 (6.1)	1,103 (52.3)	−
GASPII, n=568, 2003-2004 (Italy)	33.0 (24.0, 41.0)	326 (57.4)	−	77 (13.6)	283 (49.8)	208 (36.6)	−	563 (99.1)	1 (0.2)	64 (11.3)	−	294 (51.8)	−
GECKO Drenthe, n=1,963, 2006-2007 (The Netherlands)	31.0 (23.0, 39.0)	730 (37.2)	128 (6.5)	1,213 (61.8)	734 (37.4)	0 (0)	16 (0.8)	1,919 (97.8)	1 (0.1)	280 (14.3)	1 (0.1)	991 (50.5)	−
GENERATION R, n=6,716, 2002-2006 (The Netherlands)	30.8 (19.8, 39.3)	3,760 (56.0)	37 (0.6)	616 (9.2)	2,823 (42.0)	2,887 (43.0)	390 (5.8)	3,978 (59.2)	147 (2.2)	1,575 (23.5)	232 (3.5)	3,358 (50.0)	−

S2 Table. Cohort-specific description of available covariates (continued)

Cohort name, number of participants, (country)	Maternal age, (years)	Parity, n (%)		Maternal education level, n (%)				Ethnicity, n (%)		Smoking during pregnancy, n (%)		Child's sex, n (%)	
	Median (95% range)	Nulliparous	Missings	Low	Medium	High	Missings	European/ White	Missings	Yes	Missings	Male	Missings
GENERATION XXI, n=5,940, 2005-2006 (Portugal)	30.0 (18.0, 40.0)	3,407 (57.4)	89 (1.5)	1,773 (29.8)	2,596 (43.7)	1,542 (26.0)	29 (0.5)	NA	−	1,293 (21.8)	58 (1.0)	3,038 (51.1)	−
GENESIS, n=1,898, 2003-2004 (Greece)	30.2 (21.0, 39.0)	975 (51.4)	−	89 (4.7)	961 (50.6)	791 (41.7)	57 (3.0)	NA	−	343 (18.1)	1 (0.1)	981 (51.7)	−
GINIplus, n=2,326, 1995-1998 (Germany)	31.0 (24.0, 40.0)	NA	−	273 (11.7)	982 (42.2)	1,065 (45.8)	6 (0.3)	NA	−	257 (11.0)	29 (1.2)	1,140 (49.0)	−
HUMIS, n=945, 2003-2008 (Norway)	30.0 (22.0, 39.0)	414 (43.8)	−	93 (9.8)	150 (15.9)	559 (59.2)	143 (15.1)	710 (75.1)	149 (15.8)	92 (9.7)	40 (4.2)	473 (50.1)	−
INMA, n=1,916, 1997-2008 (Spain)	30.0 (22.0, 39.0)	1,052 (54,9)	2 (0.1)	570 (29.7)	732 (38.2)	595 (31.1)	19 (1.0)	1,830 (95.5)	3 (0.2)	338 (17.6)	18 (0.9)	983 (51.3)	−
KOALA, n=2,051, 2000-2002 (The Netherlands)	32.0 (25.0, 40.0)	896 (43.7)	43 (2.1)	177 (8.6)	755 (36.8)	1,024 (49.9)	95 (4.6)	1,983 (96.7)	6 (0.3)	122 (5.9)	4 (0.2)	1,058 (51.6)	−
Krakow Cohort, n=422, 2000-2003 (Poland)	28.0 (20.0, 34.0)	269 (63.7)	−	39 (9.2)	160 (37.9)	223 (52.8)	−	422 (100.0)	−	NA	−	215 (50.9)	−
LISAplus, n=2,334, 1997-1999 (Germany)	31.0 (23.0, 40.0)	1,020 (43.7)	8 (0.3)	172 (7.4)	867 (37.1)	1,267 (54.3)	28 (2.1)	NA	−	337 (14.4)	11 (0.5)	1,214 (52.0)	−
LUKAS, n=379, 2002-2005 (Finland)	31.0 (21.2, 42.1)	132 (34.8)	−	13 (3.4)	283 (74.7)	83 (21.9)	−	379 (100.0)	−	60 (15.8)	−	189 (49.9)	−
MoBa, n=54,910, 1999-2009 (Norway)	30.0 (22.0, 39.0)	25,455 (46.4)	−	14,895 (27.1)	24,242 (44.1)	14,725 (26.8)	1,048 (1.9)	NA	−	3,947 (7.2)	5,435 (9.9)	28,153 (51.3)	−
NINFEA, n=1,753, 2005-2010 (Italy)[a]	33.0 (25.0, 41.0)	1,183 (67.5)	-	64 (3.7)	585 (33.4)	1,100 (62.7)	4 (0.2)	1,728 (98.6)[b]	−	143 (8.2)	9 (0.5)	905 (51.6)	−
PÉLAGIE, n=738, 2002-2005 (France)	30.1 (22.8, 39.5)	325 (44.0)	−	104 (14.1)	127 (17.2)	506 (68.6)	1 (0.1)	NA	−	197 (26.7)	1 (0.1)	380 (51.5)	−
PIAMA, n=2,324, 1996-1997 (The Netherlands)	31.0 (23.0, 38.0)	1,156 (49.7)	−	457 (19.7)	967 (41.6)	900 (38.7)	-	2,213 (95.2)	45 (1.9)	361 (15.5)	16 (0.7)	1,173 (50.5)	−
Piccolipiù, n=687, 2011-2015 (Italy)	34.0 (24.0, 43.0)	430 (62.6)	-	68 (9.9)	265 (38.6)	353 (51.4)	1 (0.1)	678 (98.7)	1 (0.1)	156 (22.7)	-	360 (52.4)	−
Project Viva, n=1,382, 1999-2002 (United States)	32.4 (18.9, 41.2)	661 (47.8)	−	431 (31.2)	489 (35.4)	457 (33.1)	5 (0.4)	952 (68.9)	5 (0.4)	144 (10.4)	30 (2.2)	706 (51.1)	−
Raine Study, n=2,092, 1989-1992 (Australia)	29.0 (18.0, 40.1)	989 (47.3)	−	524 (25.0)	885 (42.3)	379 (18.1)	304 (14.5)	1,886 (90.2)	−	634 (30.3)	144 (6.9)	1,085 (51.9)	−

S2 Table. Cohort-specific description of available covariates (continued)

Cohort name, number of participants, (country)	Maternal age, (years)	Parity, n (%)		Maternal education level, n (%)				Ethnicity, n (%)		Smoking during pregnancy, n (%)		Child's sex, n (%)	
	Median (95% range)	Nulliparous	Missings	Low	Medium	High	Missings	European/ White	Missings	Yes	Missings	Male	Missings
REPRO_PL, n=283, 2007-2011 (Poland)	28.0 (20.0, 37.0)	162 (57.2)	-	32 (11.3)	98 (34.6)	153 (54.1)	-	283 (100.0)	−	33 (11.7)	−	134 (47.3)	−
RHEA, n=748, 2007-2008 (Greece)	30.0 (20.0, 40.0)	NA	−	108 (14.4)	390 (52.1)	248 (33.2)	2 (0.3)	747 (99.9	1 (0.1)	255 (34.1)	1 (0.1)	397 (53.1)	−
ROLO, n=290, 2007-2011 (Ireland)	33.3 (24.6, 40.3)	0 (0)	−	0 (0)	51 (17.6)	207 (71.4)	32 (11.0)	287 (99.0)	−	6 (2.1)	−	132 (45.5)	−
SCOPE BASELINE, n=1,045, 2008-2011 (Ireland)	31.0 (22.0, 39.0)	1,045 (100.0)	−	0 (0)	121 (11.6)	921 (88.1)	3 (0.3)	1,032 (98.8)	−	229 (21.9)	−	531 (50.8)	−
SEATON, n=933, 1998-1999 (United Kingdom)	30.5 (19.5, 40.2))	344 (36.9)	−	196 (21.0)	273 (29.3)	362 (38.8)	102 (10.9)	NA	−	342 (36.7)	-	469 (50.3)	−
Slovak PCB study, n=480, 2002-2004 (Slovakia)	26.0 (19.0, 39.0)	199 (41.5)	1 (0.2)	200 (41.7)	246 (51.3)	31 (6.5)	3 (0.6)	411 (85.6)	−	72 (15.0)	12 (2.5)	236 (49.2)	−
STEPS, n=484, 2008-2010 (Finland)	31.2 (23.4, 40.7)	297 (61.4)	−	31 (6.4)	128 (26.4)	311 (64.3)	14 (2.9)	NA	−	12 (2.5)	182 (37.6)	255 (52.7)	−
SWS, n=2,621, 1998-2007 (United Kingdom)	30.3 (22.8, 36.5)	1,385 (52.8)	2 (0.1)	290 (11.1)	1564 (59.7)	761 (29.0)	6 (0.2)	2,522 (96.2)	−	329 (12.6)	281 (10.7)	1354 (51.7)	−
**Total group**	30.2 (21.0, 39.3)	78,621 (48.9)	3,720 (2.3)	33,547 (20.7)	65,007 (40.1)	6,800 (37.5)	2,775 (1.7)	43,522 (26.8)	112,281 (69.3)	24,744 (15.3)	7535 (4.6)	82,860 (51.1)	-

Values are expressed as medians (95% range) or numbers of participants (%).[a]Subset of participants with 4-years follow-up completed. [b] Distinguishes between those born in Italy and those born outside Italy.
