# Supplementary material for: Maternal body mass index, gestational weight gain, and the risk of overweight and obesity across childhood: An individual participant data meta-analysis
Source: PLoS Med. 2019 Feb 11;16(2):e1002744. doi: 10.1371/journal.pmed.1002744 (PMC6370184; doi:10.1371/journal.pmed.1002744)
Supplement: S3 Table — (PDF) [file pmed.1002744.s008.pdf]

S3 Table. Associations of maternal pre-pregnancy BMI clinical categories with the risk of childhood overweight/obesity, additionally adjusted for gestational diabetes

	**Early childhood 2.0-5.0 years**	**Mid childhood 5.0-10.0 years**	**Late childhood 10-18.0 years**
	**Overweight/obesity OR (95% CI)**	**Overweight/obesity OR (95% CI)**	**Overweight/obesity OR (95% CI)**
**Maternal pre-pregnancy BMI**			
**Underweight** (<18.5 kg/m^2)	0.57 (0.47, 0.69)	0.44 (0.40, 0.49)	0.44 (0.35, 0.55)
	$n_{cases/total}$ =126/3,162	$n_{cases/total}$ =401/4,485	$n_{cases/total}$ =93/877
**Normal weight** (18.5-24.9 kg/m^2)	Reference	Reference	Reference
	$n_{cases/total}$ =3,092/57,293	$n_{cases/total}$ =13,870/82,438	$n_{cases/total}$ =2,505/13,497
**Overweight** (25.0-29.9 kg/m^2)	1.66 (1.55, 1.77)	1.91 (1.84, 1.98)	2.28 (2.08, 2.50)
	$n_{cases/total}$ =1,476/17,013	$n_{cases/total}$ =6,556/23,359	$n_{cases/total}$ =968/2,799
**Obesity** (≥30.0 kg/m^2)	2.40 (2.21, 2.61)	3.11 (2.97, 3.26)	4.57 (3.99, 5.24)
	$n_{cases/total}$ =864/7,058	$n_{cases/total}$ =3,612/9,248	$n_{cases/total}$ =528/1,000
**Obesity class I** (30.0-34.9 kg/m^2)	2.33 (2.12, 2.56)	2.88 (2.73, 3.04)	4.17 (3.56, 4.87)
	$n_{cases/total}$ =613/5,142	$n_{cases/total}$ =2,552/6,874	$n_{cases/total}$ =363/726
**Obesity class II** (35.0-39.9 kg/m^2)	2.53 (2.16, 2.97)	3.55 (3.23, 3.91)	5.98 (4.50, 7.95)
	$n_{cases/total}$ =190/1,489	$n_{cases/total}$ =782/1,836	$n_{cases/total}$ =129/215
**Obesity class III** (≥40.0 kg/m^2)	2.85 (2.16, 3.77)	5.14 (4.32, 6.12)	5.54 (3.25, 9.45)
	$n_{cases/total}$ =61/427	$n_{cases/total}$ =278/538	$n_{cases/total}$ =36/59

Values are odds ratios (95% confidence intervals) from multilevel binary logistic regression models that reflect the risk of childhood overweight in early childhood (2.0-5.0 years), mid childhood (5.0-10.0 years) and late childhood (10.0-18.0 years) in children of mothers in the different pre-pregnancy BMI groups, as compared with the reference group (normal weight). The models are adjusted for maternal age, education level, ethnicity, parity, smoking during pregnancy, and gestational diabetes.
