# Supplementary material for: Maternal body mass index, gestational weight gain, and the risk of overweight and obesity across childhood: An individual participant data meta-analysis
Source: PLoS Med. 2019 Feb 11;16(2):e1002744. doi: 10.1371/journal.pmed.1002744 (PMC6370184; doi:10.1371/journal.pmed.1002744)
Supplement: S4 Table — (PDF) [file pmed.1002744.s009.pdf]

S4 Table. Associations of maternal pre-pregnancy BMI clinical categories with the risk of childhood overweight/obesity, additionally adjusted for gestational hypertensive disorders

	**Early childhood 2.0-5.0 years**	**Mid childhood 5.0-10.0 years**	**Late childhood 10-18.0 years**
	**Overweight/obesity OR (95% CI)**	**Overweight/obesity OR (95% CI)**	**Overweight/obesity OR (95% CI)**
**Maternal pre-pregnancy BMI**			
**Underweight** (<18.5 kg/m^2)	0.57 (0.47, 0.69)	0.44 (0.40, 0.49)	0.44 (0.35, 0.55)
	$n_{cases/total}$ =126/3,162	$n_{cases/total}$ =401/4,485	$n_{cases/total}$ =93/877
**Normal weight** (18.5-24.9 kg/m^2)	Reference	Reference	Reference
	$n_{cases/total}$ =3,092/57,293	$n_{cases/total}$ =13,870/82,438	$n_{cases/total}$ =2,505/13,497
**Overweight** (25.0-29.9 kg/m^2)	1.66 (1.56, 1.78)	1.91 (1.84, 1.98)	2.26 (2.06, 2.48)
	$n_{cases/total}$ =1,476/17,013	$n_{cases/total}$ =6,556/23,359	$n_{cases/total}$ =968/2,799
**Obesity** (≥30.0 kg/m^2)	2.45 (2.25, 2.66)	3.12 (2.98, 3.26)	4.46 (3.89, 5.11)
	$n_{cases/total}$ =864/7,058	$n_{cases/total}$ =3,612/9,248	$n_{cases/total}$ =528/1,000
**Obesity class I** (30.0-34.9 kg/m^2)	2.37 (2.15, 2.60)	2.89 (2.74, 3.05)	4.08 (3.49, 4.77)
	$n_{cases/total}$ =613/5,142	$n_{cases/total}$ =2,552/6,874	$n_{cases/total}$ =363/726
**Obesity class II** (35.0-39.9 kg/m^2)	2.59 (2.21, 3.04)	3.56 (3.24, 3.93)	5.82 (4.37, 7.73)
	$n_{cases/total}$ =190/1,489	$n_{cases/total}$ =782/1,836	$n_{cases/total}$ =129/215
**Obesity class III** (≥40.0 kg/m^2)	2.96 (2.24, 3.91)	5.16 (4.34, 6.14)	5.27 (3.09, 9.00)
	$n_{cases/total}$ =61/427	$n_{cases/total}$ =278/538	$n_{cases/total}$ =36/59

Values are odds ratios (95% confidence intervals) from multilevel binary logistic regression models that reflect the risk of childhood overweight in early childhood (2.0-5.0 years), mid childhood (5.0-10.0 years) and late childhood (10.0-18.0 years) in children of mothers in the different pre-pregnancy BMI groups, as compared with the reference group (normal weight). The models are adjusted for maternal age, education level, ethnicity, parity, smoking during pregnancy, and gestational hypertensive disorders (gestational hypertension and pre-eclampsia).
