# Supplementary material for: Maternal body mass index, gestational weight gain, and the risk of overweight and obesity across childhood: An individual participant data meta-analysis
Source: PLoS Med. 2019 Feb 11;16(2):e1002744. doi: 10.1371/journal.pmed.1002744 (PMC6370184; doi:10.1371/journal.pmed.1002744)
Supplement: S5 Table — (PDF) [file pmed.1002744.s010.pdf]

S5 Table. Associations of maternal pre-pregnancy BMI clinical categories with the risk of childhood overweight/obesity, additionally adjusted for gestational-age-adjusted birth weight

	**Early childhood 2.0-5.0 years**	**Mid childhood 5.0-10.0 years**	**Late childhood 10-18.0 years**
	**Overweight/obesity OR (95% CI)**	**Overweight/obesity OR (95% CI)**	**Overweight/obesity OR (95% CI)**
**Maternal pre-pregnancy BMI**			
**Underweight** (<18.5 kg/m^2)	0.60 (0.49, 0.72)	0.46 (0.42, 0.51)	0.45 (0.36, 0.56)
	$n_{cases/total}$ =126/3,162	$n_{cases/total}$ =401/4,485	$n_{cases/total}$ =93/877
**Normal weight** (18.5-24.9 kg/m^2)	Reference	Reference	Reference
	$n_{cases/total}$ =3,092/57,293	$n_{cases/total}$ =13,870/82,438	$n_{cases/total}$ =2,505/13,497
**Overweight** (25.0-29.9 kg/m^2)	1.61 (1.50, 1.72)	1.86 (1.80, 1.93)	2.25 (2.05, 2.46)
	$n_{cases/total}$ =1,476/17,013	$n_{cases/total}$ =6,556/23,359	$n_{cases/total}$ =968/2,799
**Obesity** (≥30.0 kg/m^2)	2.29 (2.11, 2.49)	2.98 (2.84, 3.12)	4.42 (3.86, 5.07)
	$n_{cases/total}$ =864/7,058	$n_{cases/total}$ =3,612/9,248	$n_{cases/total}$ =528/1,000
**Obesity class I** (30.0-34.9 kg/m^2)	2.23 (2.03, 2.45)	2.77 (2.62, 2.92)	4.02 (3.44, 4.71)
	$n_{cases/total}$ =613/5,142	$n_{cases/total}$ =2,552/6,874	$n_{cases/total}$ =363/726
**Obesity class II** (35.0-39.9 kg/m^2)	2.40 (2.05, 2.82)	3.37 (3.06, 3.72)	5.82 (4.38, 7.74)
	$n_{cases/total}$ =190/1,489	$n_{cases/total}$ =782/1,836	$n_{cases/total}$ =129/215
**Obesity class III** (≥40.0 kg/m^2)	2.69 (2.04, 3.55)	4.85 (4.07, 5.77)	5.35 (3.13, 9.13)
	$n_{cases/total}$ =61/427	$n_{cases/total}$ =278/538	$n_{cases/total}$ =36/59

Values are odds ratios (95% confidence intervals) from multilevel binary logistic regression models that reflect the risk of childhood overweight in early childhood (2.0-5.0 years), mid childhood (5.0-10.0 years) and late childhood (10.0-18.0 years) in children of mothers in the different pre-pregnancy BMI groups, as compared with the reference group (normal weight). The models are adjusted for maternal age, education level, ethnicity, parity, smoking during pregnancy, and gestational-age-adjusted birth weight.
