# Supplementary material for: Maternal body mass index, gestational weight gain, and the risk of overweight and obesity across childhood: An individual participant data meta-analysis
Source: PLoS Med. 2019 Feb 11;16(2):e1002744. doi: 10.1371/journal.pmed.1002744 (PMC6370184; doi:10.1371/journal.pmed.1002744)
Supplement: S6 Table — (PDF) [file pmed.1002744.s011.pdf]

S6 Table. Associations of maternal pre-pregnancy BMI and gestational weight gain clinical categories with the risk of childhood underweight

	Early childhood 2.0-5.0 years		Mid childhood 5.0-10.0 years		Late childhood 10-18.0 years	
	**Underweight OR (95% CI)**	**PAR (%)**	**Underweight OR (95% CI)**	**PAR (%)**	**Underweight OR (95% CI)**	**PAR (%)**
**Maternal pre-pregnancy BMI**						
**Underweight** (<18.5 kg/m^2)	1.46 (1.15, 1.86)	1.8	1.94 (1.66, 2.27)	3.5	2.40 (1.69, 3.39)	5.2
	$n_{cases/total}$ =78/3,114		$n_{cases/total}$ =188/4,272		$n_{cases/total}$ =40/824	
**Normal weight** (18.5-24.9 kg/m^2)	Reference		Reference		Reference	
	$n_{cases/total}$ =884/55,085		$n_{cases/total}$ =1,681/70,249		$n_{cases/total}$ =240/11,232	
**Overweight** (25.0-29.9 kg/m^2)	0.93 (0.81, 1.08)	NA	0.71 (0.63, 0.81)	NA	0.49 (0.31, 0.78)	NA
	$n_{cases/total}$ =245/15,782		$n_{cases/total}$ =6,556/23,359		$n_{cases/total}$ =20/1,851	
**Obesity** (≥30.0 kg/m^2)	0.74 (0.58. 0.93)	NA	0.60 (0.49, 0.75)	NA	0.38 (0.14, 1.03)	NA
	$n_{cases/total}$ =80/6,274		$n_{cases/total}$ =312/17,115		$n_{cases/total}$ =4/367	
**Obesity class I** (30.0-34.9 kg/m^2)	0.69 (0.53, 0.91)	NA	0.60 (0.47, 0.76)	NA	0.49 (0.18, 1.34)	NA
	$n_{cases/total}$ =55/4,584		$n_{cases/total}$ =70/4,392		$n_{cases/total}$ =4/476	
**Obesity class II** (35.0-39.9 kg/m^2)	0.88 (0.56, 1.38)	NA	0.64 (0.40, 1.03)	NA	NA	NA
	$n_{cases/total}$ =20/1,319		$n_{cases/total}$ =18/1,072		$n_{cases/total}$ =0/86	
**Obesity class III** (≥40.0 kg/m^2)	0.77 (0.32, 1.88)	NA	0.55 (0.20, 1.47)	NA	NA	NA
	$n_{cases/total}$ =5/371		$n_{cases/total}$ =4/264		$n_{cases/total}$ =0/23	
**Gestational weight gain**						
**Inadequate weight gain**	1.24 (1.06, 1.44)	4.1	1.25 (1.11, 1.40)	4.2	1.15 (0.92, 1.43)	2.6
	$n_{cases/total}$ =298/14,929		$n_{cases/total}$=489/17,579		$n_{cases/total}$=101/3,293	
**Adequate weight gain**	Reference		Reference		Reference	
	$n_{cases/total}$ =411/24,287		$n_{cases/total}$=751/30,738		$n_{cases/total}$=95/4,303	
**Excessive weight gain**	0.84 (0.73, 0.97)	NA	0.83 (0.74, 0.92)	NA	0.88 (0.69, 1.14)	NA
	$n_{cases/total}$=375/25,700		$n_{cases/total}$=678/30,995		$n_{cases/total}$=39/2,681	

Values are odds ratios (95% confidence intervals) from multilevel binary logistic regression models that reflect the risk of childhood underweight in early childhood (2.0-5.0 years), mid childhood (5.0-10.0 years) and late childhood (10.0-18.0 years) in children of mothers in the different pre-pregnancy BMI groups or gestational weight gain groups, as compared with the reference group (normal weight for pre-pregnancy BMI and adequate weight gain for gestational weight gain) or population attributable risk fractions (PAR), indicating the proportion of childhood underweight cases attributable to each maternal BMI or gestational weight gain category. The models are adjusted for maternal age, education level, ethnicity, parity, and smoking during pregnancy. NA: not applicable.
