# Supplementary material for: Maternal body mass index, gestational weight gain, and the risk of overweight and obesity across childhood: An individual participant data meta-analysis
Source: PLoS Med. 2019 Feb 11;16(2):e1002744. doi: 10.1371/journal.pmed.1002744 (PMC6370184; doi:10.1371/journal.pmed.1002744)
Supplement: S7 Table — (PDF) [file pmed.1002744.s012.pdf]

S7 Table. Associations of maternal pre-pregnancy BMI and gestational weight gain clinical categories with the risk of childhood overweight/obesity, complete case analysis

	**Early childhood 2.0-5.0 years**	**Mid childhood 5.0-10.0 years**	**Late childhood 10-18.0 years**
	**overweight/obesity OR (95% CI)**	**overweight/obesity OR (95% CI)**	**overweight/obesity OR (95% CI)**
**Maternal pre-pregnancy BMI**			
**Underweight** (<18.5 kg/m^2)	0.65 (0.49, 0.85)	0.42 (0.35, 0.51)	0.48 (0.37, 0.61)
	$n_{cases/total}$ =68/1,266	$n_{cases/total}$ =146/1,356	$n_{cases/total}$ =73/601
**Normal weight** (18.5-24.9 kg/m^2)	Reference	Reference	Reference
	$n_{cases/total}$ =1,055/19,057	$n_{cases/total}$ =4,625/22,872	$n_{cases/total}$ =1,791/9,176
**Overweight** (25.0-29.9 kg/m^2)	1.73 (1.54, 1.95)	2.07 (1.94, 2.21)	2.36 (2.12, 2.64)
	$n_{cases/total}$ =469/5,289	$n_{cases/total}$ =1,997/5,832	$n_{cases/total}$ = 691/1,908
**Obesity** (≥30.0 kg/m^2)	2.82 (2.43, 3.26)	3.72 (3.40, 4.08)	4.84 (4.09, 5.72)
	$n_{cases/total}$ =295/2,197	$n_{cases/total}$ =1,115/2,325	$n_{cases/total}$ =359/649
**Obesity class I** (30.0-34.9 kg/m^2)	2.66 (2.27, 3.18)	3.53 (3.18, 3.92)	4.47 (3.69, 5.42)
	$n_{cases/total}$ =204/1,568	$n_{cases/total}$ =785/1,689	$n_{cases/total}$ =252/476
**Obesity class II** (35.0-39.9 kg/m^2)	3.29 (2.52, 4.30)	3.92 (3.26, 4.72)	5.50 (3.85, 7.87)
	$n_{cases/total}$ =71/477	$n_{cases/total}$ =243/491	$n_{cases/total}$ =79/134
**Obesity class III** (≥40.0 kg/m^2)	2.77 (1.71, 4.48)	5.88 (4.18, 8.28)	8.56 (4.22, 17.36)
	$n_{cases/total}$ =20/152	$n_{cases/total}$ =87/145	$n_{cases/total}$ =28/39
**Gestational weight gain**			
**Inadequate weight gain**	'0.84 (0.72, 0.98)	0.84 (0.78, 0.91)	0.92 (0.80, 1.05)
	$n_{cases/total}$ = 303/4,974	$n_{cases/total}$= 1,315/6,355	$n_{cases/total}$= 511/2,614
**Adequate weight gain**	Reference	Reference	Reference
	$n_{cases/total}$ = 475/6,676	$n_{cases/total}$= 1,938/8,425	$n_{cases/total}$= 646/3,207
**Excessive weight gain**	1.49 (1.30, 1.70)	1.53 (1.42, 1.64)	1.75 (1.55, 1.99)
	$n_{cases/total}$= 567/5,939	$n_{cases/total}$= 2,341/7,471	$n_{cases/total}$= 731/2,321

Values are odds ratios (95% confidence intervals) from multilevel binary logistic regression models with complete cases that reflect the risk of childhood overweight/obesity in early childhood (2.0-5.0 years), mid childhood (5.0-10.0 years) and late childhood (10.0-18.0 years) in children of mothers in the different pre-pregnancy BMI groups or gestational weight gain groups, as compared with the reference group (normal weight for pre-pregnancy BMI and adequate weight gain for gestational weight gain). The models are adjusted for maternal age, education level, ethnicity, parity, and smoking during pregnancy.
