# Supplementary material for: Maternal body mass index, gestational weight gain, and the risk of overweight and obesity across childhood: An individual participant data meta-analysis
Source: PLoS Med. 2019 Feb 11;16(2):e1002744. doi: 10.1371/journal.pmed.1002744 (PMC6370184; doi:10.1371/journal.pmed.1002744)
Supplement: S8 Table — (PDF) [file pmed.1002744.s013.pdf]

S8 Table. Associations of maternal pre-pregnancy BMI and gestational weight gain clinical categories with childhood BMI SDS, complete case analysis

	Childhood BMI (SDS)		
	**Early childhood** **2.0-5.0 years**	**Mid childhood** **5.0-10.0 years**	**Late childhood** **10.0-18.0 years**
**Maternal pre-pregnancy BMI**			
**Underweight** (<18.5 kg/m^2)	-0.29 (-0.34, -0.23)	-0.43 (-0.34, -0.23)	-0.45 (-0.53, -0.37)
	n =1,272	n =1,370	n=619
**Normal weight** (18.5-24.9 kg/m^2)	Reference	Reference	Reference
	n =19,127	n =23,007	n=9,280
**Overweight** (25.0-29.9 kg/m^2)	0.22 (0.19, 0.25)	0.39 (0.36, 0.42)	0.47 (0.42, 0.52)
	n=5,303	n =5,852	n=1,918
**Obesity** (≥30.0 kg/m^2)	0.34 (0.36, 0.45)	0.76 (0.72, 0.80)	0.94 (0.86, 1.02)
	n =2,200	n =2,330	n=653
**Obesity class I** (30.0-34.9 kg/m^2)	0.38 (0.33, 0.43)	0.71 (0.66, 0.76)	0.88 (0.78, 0.97)
	n =1,570	n =1,693	n=480
**Obesity class II** (35.0-39.9 kg/m^2)	0.45 (0.36, 0.54)	0.81 (0.72, 0.90)	1.05 (0.88, 1.23)
	n =478	n =492	n =134
**Obesity class III** (≥40.0 kg/m^2)	0.453 (0.38, 0.69)	1.15 (0.98, 1.31)	1.38 (1.06, 1.70)
	n =152	n =145	n =39
**Gestational weight gain**			
**Inadequate weight gain**	-0.10 (-0.14, -0.06)	-0.12 (-0.16, -0.09)	-0.10 (-0.15, -0.04)
	n =5,064	n =6,447	n=2,676
**Adequate weight gain**	Reference	Reference	Reference
	n =6,768	n =8,515	n=3,264
**Excessive weight gain**	0.18 (0.14, 0.21)	0.24 (0.21, 0.27)	0.30 (0.25, 0.36)
	n=5,981	n =7,526	n=2,345

Values are regression coefficients (95% confidence intervals) from multilevel linear regression models with complete cases that reflect differences in early childhood (2.0-5.0 years), mid childhood (5.0-10.0 years) and late childhood (10.0-18.0 years) in children of mothers in the different pre-pregnancy BMI groups or gestational weight gain groups, as compared with the reference group (normal weight for pre-pregnancy BMI and adequate weight gain for gestational weight gain). The models are adjusted for maternal age, education level, ethnicity, parity, and smoking during pregnancy.
