# Supplementary material for: Maternal body mass index, gestational weight gain, and the risk of overweight and obesity across childhood: An individual participant data meta-analysis
Source: PLoS Med. 2019 Feb 11;16(2):e1002744. doi: 10.1371/journal.pmed.1002744 (PMC6370184; doi:10.1371/journal.pmed.1002744)
Supplement: S9 Table — (PDF) [file pmed.1002744.s014.pdf]

S9 Table. Contact information for data requests per cohort

Cohort name (country)	Contact for data requests	Local institutional ethical review boards
ABCD (The Netherlands)	abcd@amc.nl	Central Committee on Research Involving Human Subjects in The Netherlands, the medical ethics review committees of the participating hospitals and the Registration Committee of the Municipality of Amsterdam
ALSPAC (United Kingdom)	http://www.bristol.ac.uk/alspac/researchers/	ALSPAC Ethics and Law Committee and Local Research Ethics Committees
AOB/F (Canada)	https://policywise.com/sage/	Child Health Research Office and the Conjoint Health Research Ethics Board of the Faculties of Medicine, Nursing, and Kinesiology, University of Calgary, and the Affiliated Teaching Institutions (Ethics ID 20821 and 22821)
BAMSE (Sweden)	bamse-projekt@imm.ki.se	Regional ethical review board in Stockholm (application numbers 2010/1474- 31/3 and 2013/1879- 32).
BIB (United Kingdom)	borninbradford@bthft.nhs.uk	Bradford Research Ethics Comittee
CHOP (Multiple)	annina.herrmann@med.uni-muenchen.de	Belgium (Comitè d'Ethique de L'Hopital Universitaire des Enfants Reine Fabiola; no. CEH 14/02), Germany (Bayerische Landesärztekammer Ethik-Kommission; no. 02070), Italy (Azienda Ospedaliera San Paolo Comitato Etico; no. 14/2002), Poland (Instytut Pomnik–Centrum Zdrowia Dziecka Komitet Etyczny; no 243/KE/2001), and Spain (Comité ético de investigación clinica del Hospital Universitario de Tarragona Joan XXIII).
Co.N.ER (Italy)	simona.rosa@unibo.it	Ethics Committee of the S. Orsola-Malpighi Teaching Hospital of Bologna (Italy) (052/2004/U/Tess)
DNBC (Denmark)	dnbc-research@ssi.dk	The Scientific Ethic Committee in Denmark, the Danish Data Protection Agency, and the DNBC Steering Committee
EDEN (France)	etude.eden@inserm.fr	Ethics Committee of the Bicêtre Hospital
FCOU (Ukraine)	zoreslava7@ukr.net	Institutional Review Boards at the University of Illinois at Chicago and the Ukrainian Institute for Pediatrics, Obstetrics, and Gynecology
GASPII (Italy)	p.lorusso@deplazio.it	Ethical Committee of the Università Cattolica del Sacro Cuore, Rome
GECKO Drenthe (The Netherlands)	gecko@tcc.umcg.nl	Medical Ethics Committee of the University Medical Center Groningen (UMCG)
Generation R (The Netherlands)	generationr@erasmusmc.nl	Medical Ethical Committee of the Erasmus Medical Center, Rotterdam
Generation XXI (Portugal)	gxxi@med.up.pt	Ethics Committee of Hospital de S. João
GENESIS (Greece)	oandrou@hua.gr	Ethical Committee of Harokopio University of Athens and all municipalities invited to participate in the study
GINIplus (Germany)	maike.ferland@helmholtz-muenchen.de	Bavarian General Medical Council, University of Leipzig, Medical Council of North-Rhine-Westphalia
HUMIS (Norway)	ingunn.brandt@fhi.no	Regional Ethics Committee for Medical Research in Norway (reference S-02122) and Norwegian Data Inspectorate
INMA (Spain)	inma@proyectoimna.org	The Municipal Institute of Sanitary Assistance of Barcelona, La Fe University Hospital of Valencia, The Donostia Hospital, and Ib-salut
KOALA (The Netherlands)	datahub@maastrichtuniversity.nl	Medical ethics committee of the Maastricht University/University Hospital of Maastricht

Krakow Cohort (Poland)	epikohorta@cm-uj.krakow.pl	The Bioethical Committee of Jagiellonian University
LISAplus (Germany)	maike.ferland@helmholtz-muenchen.de	Bavarian General Medical Council, University of Leipzig, Medical Council of North-Rhine-Westphalia
LUKAS (Finland)	pirkka.kirjavainen@thl.fi	Research Ethics Committee, Hospital District of Northern Savo, Kuopio, Finland
MoBa (Norway)	datatilgang@fhi.no	Norwegian Data Inspectorate and the Regional Ethics Committee for Medical Research
NINFEA (Italy)	info@progettoninfea.it	Ethical Committee of the San Giovanni Battista Hospital and CTO/CRF/Maria Adelaide Hospital of Turin (approval N.0048362 and following amendments)
PÉLAGIE (France)	pelagie.rennes@inserm.fr	French Consulting Committee for the Treatment of Information in Medical Research (no. 09.485) and the French National Commission for the Confidentiality of Computerised Data (no. 909347)
PIAMA (The Netherlands)	http://piama.iras.uu.nl/index-en.php	Rotterdam, MEC (Medisch Ethische Commisie Erasmus Universiteit Rotterdam/Academische Ziekenhuizen Rotterdam), Groningen, MEC (Medisch Ethische Commisie Academisch ziekenhuis Groningen) and Utrecht/Bilthoven, MEC-TNO (Medisch Ethische Commisie -Toegepast Natuurwetenschappelijk Onderzoek)
Piccolipiù (Italy)	piccolipiuroma@deplazio.it	Ethics committees of the Local Health Unit Roma E (management centre), of the Istituto Superiore di Sanità (National Institute of Public Health) and of each local centre
Project Viva (United States)	project_viva@hphc.org	Institutional Review Board of Harvard Pilgrim Health Care
Raine Study (Australia)	rainestudy@uwa.edu.au	The University of Western Australia Human Research Ethics Committee
REPRO_PL (Poland)	impx@imp.lodz.pl	Ethical Committee of the Nofer Institute of Occupational Medicine, Łódź, Poland (Decision No. 7/2007)
RHEA (Greece)	kliniki.diatrofis@med.uoc.gr	Ethical Committee of the University Hospital, Scientific Council, Heraklion, Crete, Greece
ROLO (Ireland)	rolostudy@gmail.com	National Maternity Hospital, Dublin, Ireland.
SCOPE BASELINE (Ireland)	epvchls@liverpool.ac.uk<br>baseline@ucc.ie	Clinical Research Ethics Committee<br>of the Cork Teaching Hospitals, [ref ECM5(9) 01/07/<br>2008]
SEATON (United Kingdom)	seaton@abdn.ac.uk	Grampian Research Ethics Committee
Slovak PCB study (Slovakia)	todd_jusko@urmc.rochester.edu	Institutional review boards at the University of California, Davis, and the Slovak Medical University
STEPS (Finland)	eliisa.loyttyniemi@utu.fi	The Ministry of Social Affairs and Health and the Ethics Committee of the Hospital District of Southwest Finland
SWS (United Kingdom)	sws@mrc.soton.ac.uk	Southampton and South West Hampshire Local Research Ethics Committee (06/Q1702/104)
