# Supplementary material for: Maternal body mass index, gestational weight gain, and the risk of overweight and obesity across childhood: An individual participant data meta-analysis
Source: PLoS Med. 2019 Feb 11;16(2):e1002744. doi: 10.1371/journal.pmed.1002744 (PMC6370184; doi:10.1371/journal.pmed.1002744)
Supplement: S1 Text — (PDF) [file pmed.1002744.s015.pdf]

Maternal obesity and offspring consequences
Individual participant analysis

Plan for analysis

Participating cohorts

To be determined

Romy Gaillard, Claudia Kruithof, Vincent W.V. Jaddoe

January 2015

Background

Obesity is common in both western and non-western countries, and is a major public health concern. Worldwide, the prevalence of obesity has nearly doubled in the last 20 years. The strong increase in obesity prevalence is also present among women of reproductive age. In western countries, the obesity prevalence rate in pregnant women goes up to 30%[1]. Also, based on the US Institute of Medicine guidelines, up to 40% of women gain an excessive amount of weight during pregnancy in western countries. The Institute of Medicine guidelines define optimal ranges of maternal weight gain during pregnancy according to a mother's prepregnancy body mass index, and have been established based on evidence from observational studies that relate gestational weight gain to various maternal and offspring outcomes[2]. An accumulating body of evidence suggests that maternal obesity and excessive weight gain during pregnancy also have long-term health effects for the offspring.

Maternal obesity during pregnancy is associated with an increased risk of obesity in childhood and adulthood[3]. A meta-analyses based on published results from 4 studies showed that maternal prepregnancy obesity is associated with a 3-fold higher risk of offspring obesity.[4] Also, studies using more detailed body composition measures reported that a higher maternal prepregnancy body mass index is associated with a higher offspring total body mass fat and abdominal fat mass measures[5-7]. The mechanisms underlying these associations are not known but might include increased placental transfer of nutrients during fetal development, which may cause permanent adaptations in appetite, energy metabolism, neuro-endocrine function and growth patterns in offspring. These adaptations may subsequently predispose individuals to a greater risk of obesity and adverse body fat profile in later life[3].

However, the observed associations of maternal obesity with childhood outcomes might also reflect shared family-based and lifestyle-related characteristics or genetic factors. Comparing the strength of associations of prepregnancy body mass index from both mother and father with childhood outcomes may help in disentangling underlying mechanisms[8, 9]. Stronger associations for maternal body mass index suggest direct intra-uterine mechanisms, whereas similar or stronger associations for paternal body mass index suggest a role for shared family-based, lifestyle-related characteristics or genetic factors. Previous studies examining the strengths of associations of both maternal and paternal prepregnancy body mass index with childhood outcomes have mainly been focused on childhood body mass index and have reported inconsistent results.[5, 7, 10-16] Most studies reported no differences in magnitude of parental associations with offspring body mass index [10, 11, 14, 15] During childhood, body mass index might not be an appropriate measure of fat mass. A study among 4,091 UK parent-offspring trios reported that maternal prepregnancy body mass index was more strongly associated with childhood fat mass, whereas in the same sample similar effect estimates for the associations of maternal and paternal body mass index with childhood body mass index were reported.[7, 11] A study among 4,871 mothers, fathers and their children showed that both a higher maternal and paternal prepregnancy body mass index were associated with increased adiposity levels and an adverse cardio-metabolic profile in offspring, with stronger associations present for maternal prepregnancy body mass index[5].

Next to maternal prepregnancy obesity, increased maternal gestational weight gain may also influence the risk of obesity in the offspring[6, 17-21]. A meta-analysis based on published results from 12 studies showed that excessive gestational weight gain was associated with a 33% increased risk of offspring obesity[20]. The effects may be trimester specific. A study performed among 5,154 UK mother-offspring pairs showed that especially gestational weight gain in the first 14 weeks of pregnancy was positively associated with offspring BMI, waist circumference and fat mass at 9 years [6]. A study among 5,908 Dutch mother-offspring pairs showed that independent from maternal prepregnancy weight and weight gain in later pregnancy, early-pregnancy weight gain was associated with an adverse cardio-metabolic profile in childhood[21]. In early pregnancy, maternal gestational weight gain largely reflects maternal fat deposition, whereas gestational weight gain in mid- and late-pregnancy largely reflects maternal and amniotic fluid expansion and growth of the fetus, placenta and uterus[2].

As both maternal prepregnancy body mass index and gestational weight gain may be important modifiable factors for improving maternal health and health of offspring, obtaining a better understanding of the associations with chilhdood outcomes and the underlying mechanisms is of great importance for development of preventive strategies.

General objectives

The main objective is to assess the associations of maternal prepregnancy body mass index and gestational weight gain with growth and adiposity outcomes in the offspring. Data from different cohorts will be used to assess **the strength, consistency and independence** of these associations. We will perform a meta-analysis of individual participant data among mothers and their children participating in birth cohort studies.

- **Main exposures** of interest are maternal pre/early-pregnancy body mass index, paternal body mass index, and gestational weight gain in different periods of pregnancy;
- **Main outcomes** of interest are birth weight, body mass index, waist circumference, waist-to-hip ratio, total body fat mass and android/gynoid fat mass ratio;

We will specifically explore any sex or geographical differences in the associations and whether the associations are explained by pregnancy, birth or infant characteristics. Specific details are given in the analysis plan.

Methods

General design

We identified Western cohorts from Europe, North America and Oceania selected from existing collaborations on childhood health such as EarlyNutrition Project, www.chicosproject.eu; www.birthcohorts.net (assessed until July 2014).

Further eligibility criteria:

- Mothers with singleton live-born children;
- Children born from 1989 onwards;

- Information available about maternal pre-/early pregnancy body mass index;
- At least one offspring measurement available (birth weight, body mass index);
- Ethical approval of the study by local institutional review boards and written informed consent;

In total, we identified n=49 eligible studies.

- Studies willing to participate: n=40:

  ABC, ABCD, ALSPAC, ALL OUR BABIES cohort, BAMSE, Born in Bradfield, Chop, Co.N.ER, DNBC, EDEN, FCOU, GASPII, GECKO, GenG3, Generation R, Genesis, Generation XXI, Gini/Lisa, HUMIS, INMA, KOALA, Krakow cohort, Life Child, LUKAS, MOBA, NINFEA, Pelagie, PIAMA, Piccolipiù, Project Viva, Raine study, Rhea, REPRO_PL cohort, ROLO, Slovak PCB study, STEPS, SWS, Scope Baseline, Seaton, Whistler.
- Studies waiting for response: n=5

  BARWON INFANT STUDY, CHEF, HHF2, LINA, PRIDE
- Unable to participate: n=4

  ELFE, Growing up in New Zealand study, ODENSE CHILD COHORT, UPBEAT (not able to share data)

Exposures

- Maternal pre/early pregnancy body mass index. Early pregnancy <20 weeks of gestation;
- Paternal body mass index before or during pregnancy;
- Total pregnancy weight gain (difference between maximum pregnancy weight and prepregnancy weight/ exact number of weeks between those two measurements used continuously and in categories according to IOM criteria (inadequate /adequate/excessive)
- Trimester specific weight gain:
    - Early-pregnancy weight gain (difference between early-pregnancy weight and prepregnancy weight/ exact number of weeks between those two measurements).
    - Mid-pregnancy weight gain (difference between mid-pregnancy weight and early-pregnancy weight/ the exact number of weeks between those two measurements).
    - Late-pregnancy weight gain (difference between late-pregnancy weight and mid-pregnancy weight/ exact number of weeks between those two measurements).

All anthropometric measures may be measured, derived from medical records or self-reported. If necessary sensitivity analyses based on methods of data collection (self-reported and measured) will be performed. Gestational weight gain is derived from weight measures: maternal prepregnancy weight; early-pregnancy weight (closest measurement to 13 weeks of gestation, range 6-20 weeks of gestation); mid-pregnancy weight (closest measurement to 26 weeks of gestation, range 20-32 weeks of gestation); late-pregnancy weight (closest measurement to 40 weeks of gestation, range 32- 42 weeks of gestation). Ranges may be narrowed depending on the data availability.

Outcomes

- Primary outcomes

    - Birth weight;
    - Body mass index during 3 different age periods: 2 – 5 years; >5 - 10 years; and >10 years (oldest age at body mass index measurement available). Childhood underweight, normal weight, overweight and obesity will be defined by the International Obesity Task Force cut offs. For both birth weight and body mass index, we will construct sex and age-adjusted SDS-scores based on reference charts;
- Secondary outcomes
    - Waist circumference;
    - Waist-to-hip ratio;
    - Total body fat mass measured by DXA;
    - Android/gynoid fat mass ratio measured by DXA;

    If cohorts have repeatedly collected data on these offspring adiposity measures, we will use data collected at the oldest age within the age categories.

Covariates

Maternal characteristics:

- Maternal age (years)
- Maternal educational level (low, medium, high)
- Maternal ethnicity (European/White, non-European/non-White)
- Maternal parity (nulliparous, multiparous)
- Maternal smoking during pregnancy in each trimester (no, yes, number of cigarettes)
- Maternal alcohol consumption during pregnancy (no, yes)
- Maternal total calorie intake (Kcal)
- Gestational diabetes (no, yes)
- Gestational hypertension (no, yes)
- Preeclampsia (no, yes)
- Mode of delivery (vaginal, caesarean delivery)

Paternal characteristics:

- Paternal age (years)
- Paternal educational level (low, medium, high)
- Paternal ethnicity (European/White, non-European/non-White)
- Paternal smoking during pregnancy (no, yes, number of cigarettes)
- Paternal alcohol consumption during (no, yes)

Childhood characteristics:

- Gestational age at birth (weeks)
- Gender
- Weight (kg) and height (cm) at 24 months (range 18 – 30 months)
- Height at each adiposity measure (cm)
- Breastfeeding (yes/no, duration in months)
- Age at introduction of solid foods (months)

- Determinants of childhood adiposity in each age category: 2 - 5 years; >5 - 10 years; and >10 years:
- Television watching (hours/day)
- Physical activity (hours/week)
- Total calorie intake (kcal)
- Sleep (hours/day)

Covariates will be considered as confounders or intermediates based on the conceptual framework for the analysis (**Figure 1).**

Confounders:

- For all analyses: maternal and offspring socio-demographic and lifestyle related characteristics (maternal age, educational level, ethnicity, parity, smoking and alcohol consumption during pregnancy, total calorie intake, mode of delivery, infant breastfeeding, timing of introduction of solid foods, offspring television watching, physical activity, total calorie intake, sleep). A similar approach will be used for the models focused on paternal prepregnancy BMI, using paternal instead of maternal covariates.
- For the analyses focused on maternal gestational weight gain, maternal prepregnancy BMI will be considered as a confounding factor.

*Mediators***:**

- For the analyses focused on the associations of maternal prepregnancy BMI and all childhood outcomes, the mediating role of gestational weight gain and maternal pregnancy complications will be explored.
- For the analyses focused on maternal prepregnancy BMI and childhood adiposity outcomes the mediating role of gestational age adjusted birth weight and infant weight gain will additionally be explored.
- For the analyses focused on the associations of maternal weight gain with all childhood outcomes the mediating role of maternal pregnancy complications will be explored.
- For the analyses focused on maternal weight gain and childhood adiposity outcomes the mediating role of gestational age adjusted birth weight and infant weight gain will additionally be explored.

Effect modification: For all analyses, we will assess potential interactions between maternal determinants and childhood sex.

Figure 1. Conceptual framework for the analysis[1]

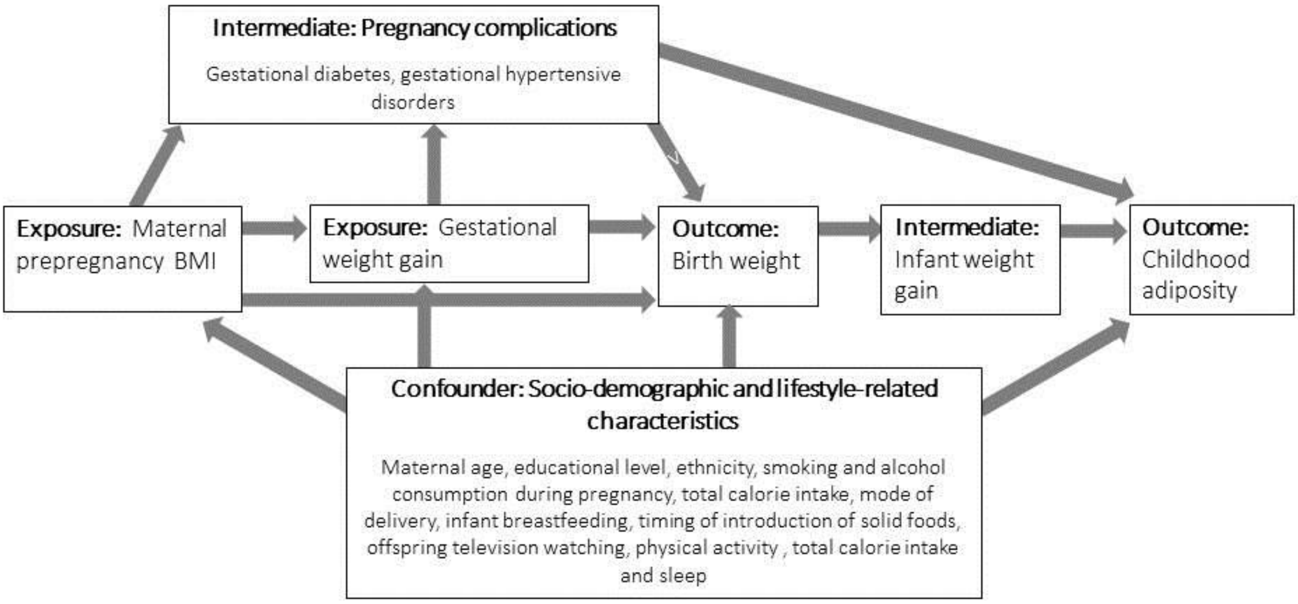

[1]More detailed information on exposures, outcomes, intermediates and confounders per analysis is given in the analysis plan.

General analysis approach

All studies will be asked to send their anonymized datasets of individual participant data to the research team at the Generation R Study Group, Erasmus University Medical Center, Rotterdam, The Netherlands, where the statistical analyses will be carried out. All analyses will be shadowed by a second analyst.

To study the associations of parental prepregnancy body mass index and maternal weight gain variables with each childhood outcome, we will primarily conduct 1-stage random-effect meta-analyses based on linear and logistic regression models. For these analyses, individual participant data from all cohorts will be combined in one analysis and will be modeled simultaneously taking into account clustering of participants within studies. The first model will be adjusted for sex and age of the child (crude model), the second model will additionally be adjusted for potential confounders (confounder model), the third model will additionally be adjusted for each potential mediator (mediator models) and the fourth model will be a fully adjusted model, containing all potential confounders and mediators. The number of studies included in the meta-analyses will differ due to variance in data availability on determinants and outcomes between cohorts. Mediation analysis will primarily be executed by exploring differences in effect estimates in the confounders models with and without the mediator of interest.

If a 1-stage approach is not possible because of the large differences in availability on covariates, we will conduct a 2-stage random-effect meta-analysis. For this, we first use linear and logistic regression models to calculate effect estimates per cohort, and second calculate pooled effect estimates from the per cohort effect estimates. We will test for heterogeneity between effect estimates using the Q test (Cochran 1954) and I^2. If the result of the Q test is statistically significant ($p < 0.05$),

indicating heterogeneity across the populations, we will use random effects analyses to take into account the potential between-study variation next to the within-study variation.

All statistical analyses will be performed using SAS 9·2 (SAS institute, Cary, NC, USA), SPSS and Comprehensive Meta-Analysis (Biostat, US).

Specific analyses will be focused on:

1. Associations of maternal prepregnancy/early pregnancy BMI with childhood outcomes. We will conduct a sensitivity analyses among studies with prepregnancy BMI only;
2. Associations of maternal prepregnancy/ early pregnancy BMI and paternal BMI with childhood outcomes and the comparison of the strength of the maternal-offspring and paternal-offspring associations. We will construct SDS-values ((observed value-mean)/SD) for parental BMI and childhood outcomes to enable comparison of effect estimates. We will examine the associations of maternal and paternal BMI singularly and simultaneously with childhood outcomes in different regression models. We will test whether the maternal associations differ significantly from the paternal associations by assessing the heterogeneity between effect estimates;
3. Associations of maternal total gestational weight gain with childhood outcomes. For total gestational weight gain we will perform 2 different analyses. We will assess the associations of maternal gestational weight defined according to the IOM criteria (inadequate, adequate, excessive gestational weight gain) among those studies that have prepregnancy BMI and total gestational weight gain available. We will also assess the associations of maternal total gestational weight continuously (kg/wk) among all studies that have a measure of gestational weight gain into third trimester (at least after 32 weeks of gestation). We will perform a sensitivity analysis among only those studies that have total gestational weight gain available;
4. Associations of maternal trimester-specific weight gain with childhood outcomes. We will use conditional change models to assess the independent associations of maternal weight gain in each trimester with childhood outcomes. For this, we will construct maternal weight gain variables for each period, which are statistically independent from each other, by using standardized residuals obtained from regression of maternal weight at a specific time point on all prior maternal weight measurements. As these conditional maternal weight gain measures are statistically independent of each other, this approach allows inclusion of all maternal weight gain measures simultaneously in one regression model.[22,23] For interpretation aims, we will also assess the associations of maternal trimester-specific weight gain with offspring outcomes using regular regression analyses. For these analyses, we will only include those studies that have maternal gestational weight gain in early, mid and late-pregnancy available.

Authors and publications

All cohorts have been invited to participate in the core analyst and writing team. Each principal investigator of a participating cohort will assign 1-3 researchers to join the case-study working group. The cohort specific researchers will extract and format the set of key variables of their own cohort according to protocol. Depending on the maximum number of authors allowed by the journal of

choice, all working group members will be a co-author (maximum 3 per cohort). If the maximum number of authors is exceeded, there will be discussion with all cohorts to form a writing group (maximum authors depending on the journal) and a collaborators group, but this option is not preferred. The results from this meta-analysis may lead to several manuscripts.

Time schedule

The project will run from August 2014 to December 2015.

August/September 2014	*First contact cohorts (send protocol, data inventory, plan for analysis)*
November/December 2014	*Discuss analysis protocol by TC, start collecting data*
February 2015	Send data transfer agreement, Formatting dataset
March 2015	Start analyses
May 2015	Results preliminary data analyses
July 2015	Results final analyses and interpretation of findings
August 2015	Submission of first manuscript

References

1. Huda SS, Brodie LE, Sattar N. Obesity in pregnancy: Prevalence and metabolic consequences. *Seminars in fetal & neonatal medicine*. 2010;15:70-76
2. Institute of Medicine (US) and National Research Council (US) Committee to Reexamine IOM Pregnancy Weight Guidelines; Rasmussen KM YA, editors. Weight Gain During Pregnancy: Reexamining the Guidelines. Washington (DC): National Academies Press (US); 2009.
3. Poston L. Maternal obesity, gestational weight gain and diet as determinants of offspring long term health. *Best Pract Res Clin Endocrinol Metab*. 2012;26:627-639
4. Yu Z, Han S, Zhu J, Sun X, Ji C, Guo X. Pre-pregnancy body mass index in relation to infant birth weight and offspring overweight/obesity: A systematic review and meta-analysis. *PLoS One*. 2013;8:e61627
5. Gaillard R, Steegers EA, Duijts L, Felix JF, Hofman A, Franco OH, Jaddoe VW. Childhood cardiometabolic outcomes of maternal obesity during pregnancy: the Generation R Study. *Hypertension*. 2014;63(4):683-91.
6. Fraser A, Tilling K, Macdonald-Wallis C, Sattar N, Brion MJ, Benfield L, Ness A, Deanfield J, Hingorani A, Nelson SM, Smith GD, Lawlor DA. Association of maternal weight gain in pregnancy with offspring obesity and metabolic and vascular traits in childhood. *Circulation*. 2010;121:2557-2564
7. Lawlor DA, Timpson NJ, Harbord RM, Leary S, Ness A, McCarthy MI, Frayling TM, Hattersley AT, Smith GD. Exploring the developmental overnutrition hypothesis using parental-offspring associations and fto as an instrumental variable. *PLoS Med*. 2008;5:e33
8. Brion MJ. Commentary: Can maternal-paternal comparisons contribute to our understanding of maternal pre-pregnancy obesity and its association with offspring cognitive outcomes? *Int J Epidemiol*. 2013;42:518-519
9. Lawlor DA. The society for social medicine john pemberton lecture 2011. Developmental overnutrition--an old hypothesis with new importance? *Int J Epidemiol*. 2013;42:7-29
10. Patel R, Martin RM, Kramer MS, Oken E, Bogdanovich N, Matush L, Smith GD, Lawlor DA. Familial associations of adiposity: Findings from a cross-sectional study of 12,181 parental-offspring trios from belarus. *PLoS One*. 2011;6:e14607
11. Davey Smith G, Steer C, Leary S, Ness A. Is there an intrauterine influence on obesity? Evidence from parent child associations in the avon longitudinal study of parents and children (alspac). *Arch Dis Child*. 2007;92:876-880
12. Lawlor DA, Smith GD, O'Callaghan M, Alati R, Mamun AA, Williams GM, Najman JM. Epidemiologic evidence for the fetal overnutrition hypothesis: Findings from the mater-university study of pregnancy and its outcomes. *Am J Epidemiol*. 2007;165:418-424

13. Murrin CM, Kelly GE, Tremblay RE, Kelleher CC. Body mass index and height over three generations: Evidence from the lifeways cross-generational cohort study. *BMC Public Health*. 2012;12:81

14. Fleten C, Nystad W, Stigum H, Skjaerven R, Lawlor DA, Davey Smith G, Naess O. Parent-offspring body mass index associations in the norwegian mother and child cohort study: A family-based approach to studying the role of the intrauterine environment in childhood adiposity. *Am J Epidemiol*. 2012;176:83-92

15. Kivimaki M, Lawlor DA, Smith GD, Elovainio M, Jokela M, Keltikangas-Jarvinen L, Viikari JS, Raitakari OT. Substantial intergenerational increases in body mass index are not explained by the fetal overnutrition hypothesis: The cardiovascular risk in young finns study. *The American journal of clinical nutrition*. 2007;86:1509-1514

16. Labayen I, Ruiz JR, Ortega FB, Loit HM, Harro J, Veidebaum T, Sjostrom M. Intergenerational cardiovascular disease risk factors involve both maternal and paternal bmi. *Diabetes care*. 2010;33:894-900

17. Crozier SR, Inskip HM, Godfrey KM, Cooper C, Harvey NC, Cole ZA, Robinson SM. Weight gain in pregnancy and childhood body composition: Findings from the southampton women's survey. *The American journal of clinical nutrition*. 2010;91:1745-1751

18. Hochner H, Friedlander Y, Calderon-Margalit R, Meiner V, Sagy Y, Avgil-Tsadok M, Burger A, Savitsky B, Siscovick DS, Manor O. Associations of maternal prepregnancy body mass index and gestational weight gain with adult offspring cardiometabolic risk factors: The jerusalem perinatal family follow-up study. *Circulation*. 2012;125:1381-1389

19. Oken E, Rifas-Shiman SL, Field AE, Frazier AL, Gillman MW. Maternal gestational weight gain and offspring weight in adolescence. *Obstet Gynecol*. 2008;112:999-1006

20. Tie HT, Xia YY, Zeng YS, Zhang Y, Dai CL, Guo JJ, Zhao Y. Risk of childhood overweight or obesity associated with excessive weight gain during pregnancy: A meta-analysis. *Arch Gynecol Obstet*. 2014;289:247-257

21. Gaillard R, Steegers EA, Franco OH, Hofman A, Jaddoe VW. Maternal weight gain in different periods of pregnancy and childhood cardio-metabolic outcomes. The generation r study. *International journal of obesity*. 2014

22. Keijzer-Veen MG, Euser AM, van Montfoort N, Dekker FW, Vandenbroucke JP, Van Houwelingen HC. A regression model with unexplained residuals was preferred in the analysis of the fetal origins of adult diseases hypothesis. *Journal of clinical epidemiology*. 2005;58:1320-1324

23. Jones A, Charakida M, Falaschetti E, Hingorani AD, Finer N, Masi S, et al. Adipose and height growth through childhood and blood pressure status in a large prospective cohort study. *Hypertension*. 2012;59(5):919-25.
