# Supplementary material for: Maternal body mass index, gestational weight gain, and the risk of overweight and obesity across childhood: An individual participant data meta-analysis
Source: PLoS Med. 2019 Feb 11;16(2):e1002744. doi: 10.1371/journal.pmed.1002744 (PMC6370184; doi:10.1371/journal.pmed.1002744)
Supplement: S2 Text — (PDF) [file pmed.1002744.s016.pdf]

S2 Text. Sources of funding/support per cohort

ABCD
This work was supported by the Netherlands Organization for Health Research and Development (ZonMw) (TOP grant, 40-00812-98-11010).

ALSPAC
The UK Medical Research Council and Wellcome (Grant ref: 102215/2/13/2) and the University of Bristol provide core support for ALSPAC. This study has received support from the US National Institute of Health (R01 DK10324) and European Research Council under the European Union's Seventh Framework Programme (FP7/2007-2013) / ERC grant agreement no 669545.

AOB/F
All Our Families is funded through Alberta Innovates Interdisciplinary Team Grant #200700595, the Alberta Children's Hospital Foundation, and the Max Bell Foundation.

BAMSE
This work was supported by the Swedish Research Council, The Swedish Heart and Lung Foundation, The Swedish Research Council for Working Life and Social Welfare, the Swedish Asthma and Allergy Association Research Foundation, The Swedish Research Council Formas, Stockholm County Council, and the European Commission's Seventh Framework 29 Program MeDALL under grant agreement No. 261357.

BIB
BiB receives core infrastructure funding from the Wellcome Trust (WT101597MA) and a joint grant from the UK Medical Research Council (MRC) and Economic and Social Science Research Council (ESRC) (MR/N024397/1). This study has received support from the British Heart Foundation (CS/16/4/32482), US National Institute of Health (R01 DK10324) and European Research Council under the European Union's Seventh Framework Programme (FP7/2007-2013) / ERC grant agreement no 669545. The research was funded by the NIHR CLAHRC Yorkshire and Humber. www.clahrc-yh.nihr.ac.uk and received support from the NIHR Clinical Research Network. The views expressed are those of the author(s), and not necessarily those of the NHS, the NIHR or the Department of Health and Social Care.

DNBC
The Danish National Research Foundation has established the Danish Epidemiology Science Centre that initiated and created the Danish National Birth Cohort. The cohort is furthermore a result of a major grant from this foundation. Additional support for the Danish National Birth Cohort is obtained from the Pharmacy Foundation, the Egmont Foundation, the March of Dimes Birth Defects Foundation, the Augustinus Foundation, and the Health Foundation. The DNBC 7-year follow-up is supported by the Lundbeck Foundation (195/04) and the Danish Medical Research Council (SSVF 0646).

EDEN
The EDEN study was supported by Foundation for medical research (FRM), National Agency for Research (ANR), National Institute for Research in Public health (IRESP: TGIR cohorte santé 2008 program), French Ministry of Health (DGS), French Ministry of Research, INSERM Bone and Joint Diseases National Research (PRO-A) and Human Nutrition National Research Programs, Paris-Sud University, Nestlé, French National Institute for Population Health Surveillance (InVS), French National Institute for Health Education (INPES), the European Union FP7 programmes (FP7/2007-2013, HELIX, ESCAPE, ENRIECO, Medall projects), Diabetes National Research Program (through a collaboration with the French Association of Diabetic Patients (AFD)), French Agency for

Environmental Health Safety (now ANSES), Mutuelle Générale de l'Education Nationale a complementary health insurance (MGEN), French national agency for food security, French speaking association for the study of diabetes and metabolism (ALFEDIAM).

FCOU
FCOU study is supported by the U.S. National Institutes of Health Fogarty International Center, US NIEHS, US CDC, US EPA, and National Academy of Medical Sciences of Ukraine.

GASPII
This work was supported by the Ministry of Health.

GECKO Drenthe
The GECKO Drenthe birth cohort was funded by an unrestricted grant of Hutchison Whampoa Ld, Hong Kong and supported by the University of Groningen , Well Baby Clinic Foundation Icare, Noordlease, Paediatric Association Of The Netherlands and Youth Health Care Drenthe.

Generation R
The general design of the Generation R Study is made possible by financial support from the Erasmus MC, University Medical Center, Rotterdam, Erasmus University Rotterdam, Netherlands Organization for Health Research and Development (ZonMw), Netherlands Organisation for Scientific Research (NWO), Ministry of Health, Welfare and Sport and Ministry of Youth and Families. Research leading to these results has received funding from the European Union's Seventh Framework Programme (FP7/2007-2013), project EarlyNutrition under grant agreement n°289346, the European Union's Horizon 2020 research and innovation programme under grant agreement No 633595 (DynaHEALTH) and the European Union's Horizon 2020 research and innovation programme under grant agreement 733206 (LifeCycle Project).

Generation XXI
Generation XXI was funded by Programa Operacional de Saúde – Saúde XXI, Quadro Comunitário de Apoio III and Administração Regional de Saúde Norte (Regional Department of Ministry of Health). This study was funded by FEDER through the Operational Programme Competitiveness and Internationalization and national funding from the Foundation for Science and Technology – FCT (Portuguese Ministry of Science, Technology and Higher Education) (POCI-01- 0145-FEDER-016837), under the project "PathMOB.: Risco cardiometabólico na infância: desde o início da vida ao fim da infância" (Ref. FCT PTDC/DTP-EPI/3306/2014) and the Unidade de Investigação em Epidemiologia - Instituto de Saúde Pública da Universidade do Porto (EPIUnit) (POCI-01-0145-FEDER-006862; Ref. UID/DTP/04750/2013)..

GENESIS
The study was supported by a research grant from Friesland Foods Hellas.

GINIplus
The GINIplus study was mainly supported for the first 3 years of the Federal Ministry for Education, Science, Research and Technology (interventional arm) and Helmholtz Zentrum Munich (former GSF) (observational arm). The 4 year, 6 year, 10 year and 15 year follow-up examinations of the GINIplus study were covered from the respective budgets of the 5 study centres (Helmholtz Zentrum Munich (former GSF), Research Institute at Marien-Hospital Wesel, LMU Munich, TU Munich and from 6 years onwards also from IUF - Leibniz Research-Institute for Environmental Medicine at the University of Düsseldorf) and a grant from the Federal Ministry for Environment (IUF Düsseldorf, FKZ 20462296). Further, the 15 year follow-up examination of the GINIplus study was supported by the Commission of the European Communities, the 7th Framework Program: MeDALL project, and as well by the companies Mead Johnson and Nestlé.

HUMIS
This work was supported by the European Community's Seventh Framework Programme (FP7/2007-2013) under grant agreements Early Nutrition n° 289346 and by funds from the Norwegian Research Council's MILPAAHEL programme, project No.213148.

INMA-Sabadell
This study was funded by grants from the Instituto de Salud Carlos III (Red INMA G03/176) and the Generalitat de Catalunya-CIRIT (1999SGR 00241).

INMA-Valencia
This study was funded by Grants from UE (FP7-ENV-2011 cod 282957 and HEALTH.2010.2.4.5-1), Spain: ISCIII (G03/176; FIS-FEDER: PI09/02647, PI11/01007, PI11/02591, PI11/02038, PI13/1944, PI13/2032, PI14/00891, PI14/01687, and PI16/1288; Miguel Servet-FEDER CP11/00178, CP15/00025, and CPII16/00051), and Generalitat Valenciana: FISABIO (UGP 15-230, UGP-15-244, and UGP-15-249).

INMA-Gipuzkoa
This study was funded by grants from the Instituto de Salud Carlos III (FISFIS PI06/0867, FIS-PS09/0009) 0867,Red INMA G03/176) and the Departamento de Salud del Gobierno Vasco (2005111093 and 2009111069) and the Provincial Government of Guipúzcoa (DFG06/004 and FG08/001).

INMA-Menorca
This study was funded by grants from the Instituto de Salud Carlos III (Red INMA G03/176).

KOALA
Data collection for the KOALA study from pregnancy up to age 1y was financially supported by grants from Royal Friesland Foods (Leeuwarden); Triodos Foundation (Zeist); Phoenix Foundation; Raphaël Foundation; Iona Foundation; Foundation for the Advancement of Heilpedagogie; Netherlands Organisation for Health Research and Development (ZonMw no. 2100.0090); Netherlands Asthma Foundation (grants no. 3.2.03.48 and 3.2.07.022); Netherlands Heart Foundation (grant no. 2008B112).The funders had no influence on the design, analysis and reporting of the current study.

Krakow Cohort
The study received funding from a NIEHS R01 grants entitled: “Vulnerability of the Fetus/Infant to PAH, PM2.5 and ETS” and “Developmental effects of early-life exposure to airborne PAH” (R01ES010165 and R01ES015282) and from The Lundin Foundation, The John and Wendy Neu Family Foundation, The Gladys and Roland Harriman Foundation and an Anonymous Foundation.

LISAplus
The LISAplus study was mainly supported by grants from the Federal Ministry for Education, Science, Research and Technology and in addition from Helmholtz Zentrum Munich (former GSF), Helmholtz Centre for Environmental Research - UFZ, Leipzig, Research Institute at Marien-Hospital Wesel, Pediatric Practice, Bad Honnef for the first 2 years. The 4 year, 6 year, 10 year and 15 year follow-up examinations of the LISAplus study were covered from the respective budgets of the involved partners (Helmholtz Zentrum Munich (former GSF), Helmholtz Centre for Environmental Research - UFZ, Leipzig, Research Institute at Marien-Hospital Wesel, Pediatric Practice, Bad Honnef, IUF – Leibniz-Research Institute for Environmental Medicine at the University of Düsseldorf) and in addition by a grant from the Federal Ministry for Environment (IUF Düsseldorf, FKZ 20462296). Further, the 15 year follow-up examination of the LISAplus study was supported by the Commission of the European Communities, the 7th Framework Program: MeDALL project.

LUKAS
This work was supported by grants from the Academy of Finland (grants 139021;287675); the Juho Vainio Foundation; the Foundation for Pediatric Research; EVO/VTR-funding; Päivikki and Sakari Sohlberg Foundation; The Finnish Cultural Foundation; European Union QLK4-CT-2001-00250; and by the National Institute for Health and Welfare, Finland.

MoBa
The Norwegian Mother and Child Cohort Study is supported by the Norwegian Ministry of Health and Care Services and the Ministry of Education and Research, NIH/NIEHS (contract no N01-ES-75558), NIH/NINDS (grant no.1 UO1 NS 047537-01 and grant no.2 UO1 NS 047537-06A1).

NINFEA
The NINFEA cohort was partially funded by the Compagnia San Paolo Fundation and by the Piedmont Region.

PÉLAGIE
The Pélagie cohort was supported by the French National Research Agency (ANR-2010-PRSP-007), and the French Research Institute for Public Health (AMC11004NSA-DGS).

PIAMA
The PIAMA study was supported by the Netherlands Organization for Health Research and Development; The Netherlands Organization for Scientific Research; The Netherlands Asthma Fund; The Netherlands Ministry of Spatial Planning, Housing, and the Environment; and The Netherlands Ministry of Health, Welfare, and Sport.

Piccolipiù
The Piccolipiù project was financially supported by the Italian National Center for Disease Prevention and Control (CCM grants years 2010 and 2014) and by the Italian Ministry of Health (art 12 and 12 bis D.lgs 502/92).

Project Viva
National Institutes of Health (R01 HD034568, UG3OD023286).

RAINE Study
The Western Australian Pregnancy Cohort (Raine Study) has been funded by program and project grants from the Australian National Health and Medical Research Council, the Commonwealth Scientific and Industrial Research Organisation, Healthway and the Lions Eye Institute in Western Australia. The University of Western Australia (UWA), Curtin University, the Raine Medical Research Foundation, the Telethon Kids Institute, the Women's and Infant's Research Foundation (KEMH), Murdoch University, The University of Notre Dame Australia and Edith Cowan University provide funding for the Core Management of the Raine Study.

REPRO_PL
This work was supported by the National Science Centre, Poland, under the grant DEC-2014/15/B/NZ7/00998, FP7 HEALS Grant N° 603946 and the Ministry of Science and Higher Education under grant agreement no. 3068/7.PR/2014/2.

RHEA
The "Rhea" project was financially supported by European projects (EU FP6-2003-Food-3-NewGeneris, EU FP6. STREP Hiwate, EU FP7 ENV.2007.1.2.2.2. Project No 211250 Escape, EU FP7-2008-ENV-1.2.1.4 Envirogenomarkers, EU FP7-HEALTH-2009- single stage CHICOS, EU FP7 ENV.2008.1.2.1.6. Proposal No 226285 ENRIECO, EU- FP7- HEALTH-2012 Proposal No 308333 HELIX) and the Greek Ministry of Health (Program of Prevention of obesity and neurodevelopmental disorders in preschool children, in Heraklion district, Crete, Greece: 2011-2014; "Rhea Plus": Primary

Prevention Program of Environmental Risk Factors for Reproductive Health, and Child Health: 2012-15).

ROLO
ROLO is supported by the Health Research Board Ireland, the Health Research Centre for Health and Diet Research, and the European Union's Seventh Framework Programme (FP7/2007-2013), project EarlyNutrition under grant agreement no. 289346.

SCOPE BASELINE
The SCOPE Ireland study was funded by the Irish Health Research Board. The Cork BASELINE Birth Cohort Study was funded by the National Children's Research Centre, Dublin, Ireland. SCOPE Ireland was funded by the Health Research Board, Ireland (CSA 2007/2). The BASELINE cohort was supported by the National Children's Research Centre, Dublin, Ireland, and the Food Standards Agency of the United Kingdom (grant no. TO7060). SCOPE and BASELINE are supported by INFANT, an SFI funded Research Centre (grant no 12/RC/2272).

SEATON
The ten year follow up of this cohort was funded by the Medical Research Council (grant 80219), the recruitment and follow up at one, two and five years was funded by Asthma UK (grants 00/011, 02/017).

Slovak PCB study
Support was provided by U.S. National Institutes of Health grants R01 CA096525, R03 TW007152, P30 ES001247, and K12 ES019852.

STEPS
This study was supported by the University of Turku, Abo Akademi University, the Turku University Hospital, and the City of Turku, as well as by the Academy of Finland (grants 121569 and 123571), the Juho Vainio Foundation, the Yrjö Jahnsson Foundation, the Turku.

SWS
The SWS is supported by grants from the Medical Research Council, National Institute for Health Research Southampton Biomedical Research Centre, University of Southampton and University Hospital Southampton National Health Service Foundation Trust, and the European Union’s Seventh Framework Programme (FP7/2007-2013), project EarlyNutrition (grant 289346). Study participants were drawn from a cohort study funded by the Medical Research Council and the Dunhill Medical Trust.

ROLE OF THE FUNDER/SPONSOR

FCOU
Investigators from the U.S. National Institute of Environmental Health Scientists (NIEHS) were involved in the design of the birth outcomes phase of the FCOU study.

The other cohorts declared that the funding agencies had no role in the design and conduct of the study; collection, management, analysis and interpretation of data; preparation, review, approval of manuscript; or decision to submit manuscript for publication.
