# Supplementary material for: Maternal body mass index, gestational weight gain, and the risk of overweight and obesity across childhood: An individual participant data meta-analysis
Source: PLoS Med. 2019 Feb 11;16(2):e1002744. doi: 10.1371/journal.pmed.1002744 (PMC6370184; doi:10.1371/journal.pmed.1002744)
Supplement: S3 Text — (PDF) [file pmed.1002744.s017.pdf]

S3 Text. Acknowledgements per cohort

ABCD

The authors especially thank all participating mothers and their children, and are grateful to all obstetric care providers in Amsterdam for their contribution to the data collection of the ABCD-study.

ALSPAC

The authors are extremely grateful to all the families who took part in this study, the midwives for their help in recruiting them, and the whole ALSPAC team, which includes interviewers, computer and laboratory technicians, clerical workers, research scientists, volunteers, managers, receptionists and nurses.

AOB/F

The authors acknowledge the contribution and support of All Our Families participants and team.

BAMSE

The authors thank all the children and their parents for participating in the BAMSE cohort and the nurses and other staff members working in the BAMSE project.

BIB

The authors acknowledge that Born in Bradford is only possible because of the enthusiasm and commitment of the children and parents in Born in Bradford. We are grateful to all participants, health professionals and researchers who have made Born in Bradford happen.

DNBC

The authors thank all the families for participating in the Danish National Birth Cohort.

EDEN

The authors thank the EDEN mother-child cohort study group, whose members are: I. Annesi-Maesano, J.Y. Bernard, J. Botton, M.A. Charles, P. Dargent-Molina, B. de Lauzon-Guillain, P. Ducimetière, M. de Agostini, B. Foliguet, A. Forhan, X. Fritel, A. Germa, V. Goua, R. Hankard, B. Heude, M. Kaminski, B. Larroque†, N. Lelong, J. Lepeule, G. Magnin, L. Marchand, C. Nabet, F Pierre, R. Slama, M.J. Saurel-Cubizolles, M. Schweitzer, O. Thiebaugeorges.

FCOU

The authors wish to acknowledge the University of Illinois at Chicago School of Public Health's Louise Hamilton Kyiv Data Management Center for their assistance in the data management for FCOU study.

GASPII

The authors acknowledge the families involved in the study.

GECKO Drenthe

The authors are grateful to the families who took part in the GECKO Drenthe study, the midwives, gyneacologists, nurses and GPs for their help for recruitment and measurement of participants, and the whole team from the GECKO Drenthe study.

Generation R

The authors gratefully acknowledge the contribution of general practitioners, hospitals, midwives, and pharmacies in Rotterdam.

Generation XXI

The authors gratefully acknowledge the families enrolled in Generation XXI for their kindness, all members of the research team for their enthusiasm and perseverance and the participating hospitals and their staff for their help and support.

GENESIS

The authors thank the Genesis research group which was comprised from Evdokia Oikonomou, Vivian Detopoulou, Christine Kortsalioudaki, Margarita Bartsota, Thodoris Liarigkovinos and Christos Vassilopoulos.

GINIplus

The authors thank all the families for their participation in the GINIplus study. Furthermore, the authors thank all members of the GINIplus Study Group for their excellent work. The GINIplus Study group consists of the following: Institute of Epidemiology I, Helmholtz Zentrum München, German Research Center for Environmental Health, Neuherberg (Heinrich J, Brüske I, Schulz H, Flexeder C, Zeller C, Standl M, Schnappinger M, Ferland M, Thiering E, Tiesler C); Department of Pediatrics, Marien-Hospital, Wesel (Berdel D, von Berg A); Ludwig-Maximilians-University of Munich, Dr von Hauner Children's Hospital (Koletzko S); Child and Adolescent Medicine, University Hospital rechts

der Isar of the Technical University Munich (Bauer CP, Hoffmann U); IUF- Environmental Health Research Institute, Düsseldorf (Schikowski T, Link E, Klümper C, Krämer U, Sugiri D).

HUMIS

The authors thank the mothers who participated in the study and the Norwegian Research Council for their continuous support through several grants.

INMA-Valencia

The authors would particularly like to thank all the participants for their generous collaboration.

INMA-Gipuzkoa

The authors thank the children and parents who participated to the INMA-Gipuzkoa study.

INMA-Menorca

The authors thank all the participants for their generous collaboration. The authors are grateful to Mireia Garcia, Maria Victoria Estraña, Maria Victoria Iturriaga, Cristina Capo and Josep LLuch for their assistance in contacting the families and administering the questionnaires.

KOALA

The authors thank the children and parents who participated to the KOALA study.

Krakow Cohort

The authors acknowledge Jagiellnonian University Medical College in Krakow and Columbia University in New York. Principal investigator: Prof. FP Perera; co-investigator: Prof. W Jedrychowski.

LISAplus

The authors thank all the families for their participation in the LISAplus study. Furthermore, the authors thank all members of the LISAplus Study Group for their excellent work. The LISAplus Study group consists of the following: Helmholtz Zentrum München, German Research Center for Environmental Health, Institute of Epidemiology I, Munich (Heinrich J, Schnappinger M, Brüske I, Ferland M, Schulz H, Zeller C, Standl M, Thiering E, Tiesler C, Flexeder C); Department of Pediatrics, Municipal Hospital "St. Georg", Leipzig (Borte M, Diez U, Dorn C, Braun E); Marien Hospital Wesel, Department of Pediatrics, Wesel (von Berg A, Berdel D, Stiers G, Maas B); Pediatric Practice, Bad Honnef (Schaaf B); Helmholtz Centre of Environmental Research – UFZ, Department

of Environmental Immunology/Core Facility Studies, Leipzig (Lehmann I, Bauer M, Röder S, Schilde M, Nowak M, Herberth G , Müller J); Technical University Munich, Department of Pediatrics, Munich (Hoffmann U, Paschke M, Marra S); Clinical Research Group Molecular Dermatology, Department of Dermatology and Allergy, Technische Universität München (TUM), Munich (Ollert M, J. Grosch).

LUKAS

The authors thank all the families for their participation in the study. The authors are grateful to Raija Juntunen, Asko Vepsäläinen, Pekka Tiittanen, and Timo Kauppila for their contribution to the data collection and data management.

MoBa

The authors are grateful to all the participating families in Norway who take part in this on-going cohort study.

NINFEA

The authors thank all families participating in the NINFEA cohort.

PÉLAGIE

The authors thank the gynecologists, obstetricians, ultrasonographers, midwives, pediatricians, and families who participated in the study.

PIAMA

The authors thank the PIAMA participants for their ongoing collaboration.

Piccolipiù

The authors acknowledge the Piccolipiù Working Group and the families involved in the study.

Project Viva

The authors thank the Project Viva mothers, children and families for their ongoing participation.

RAINE Study

The authors would like to acknowledge the Raine Study participants and their families. The authors would also like to acknowledge the Raine Study Team for study co-ordination and data collection, and the NH&MRC for their long term contribution to funding the study over the last 29 years.

REPRO_PL

The authors would particularly like to thank all the cohort participants for their collaboration.

RHEA

The authors would particularly like to thank all the cohort participants for their generous collaboration.

ROLO

The authors are grateful to the families and healthcare staff who partake in ROLO and ROLO kids.

SCOPE BASELINE

The authors are grateful to the families and healthcare staff who kindly gave their time to the study.

Slovak PCB study

The authors thank the Slovak PCB study participants for their ongoing cooperation.

STEPS

The authors are grateful to all the families who took part in STEPS study.

SWS

The authors are grateful to the women of Southampton who gave their time to take part in the Southampton Women's Survey and to the research nurses and other staff who collected and processed the data.
